# Prospective active transfer learning on the formal coupling of amines and carboxylic acids to form secondary alkyl bonds

**DOI:** 10.1039/d5dd00309a

**Published:** 2025-11-07

**Authors:** Eunjae Shim, Ambuj Tewari, Paul M. Zimmerman, Tim Cernak

**Affiliations:** a Department of Chemistry, University of Michigan Ann Arbor MI USA paulzim@umich.edu tcernak@umich.edu; b Department of Statistics, University of Michigan Ann Arbor MI USA; c Department of Electrical Engineering and Computer Science, University of Michigan Ann Arbor MI USA; d Department of Medicinal Chemistry, University of Michigan Ann Arbor MI USA

## Abstract

Tailoring a reaction condition to suit new substrates can be labor-intensive. While machine learning can aid this endeavor, conventional strategies require large datasets to make useful predictions. Active transfer learning (ATL) tackles this problem by leveraging previously collected reaction data and adaptively selecting reagent combinations. Here, ATL is prospectively applied to find improved reagent combinations for C(sp^3^)–C(sp^3^) cross-couplings between activated amines and carboxylic acids. The formation of carbon–carbon bonds from amines and acids is a powerful complement to the classic amide coupling, but the formation of sterically congested secondary alkyl groups studied here represents a challenge for catalysis. Our results demonstrate ATL consistently improved yields within three batches of experiments, making the method of practical utility for chemical space exploration studies, such as drug discovery.

## Abstract 2

Active transfer learning (ATL) uses prior information, iterative data collection and model update to find improved conditions. Here, ATL is experimentally demonstrated on challenging amine-acid cross-coupling reactions, quickly improving yields.

## Graphical abstract

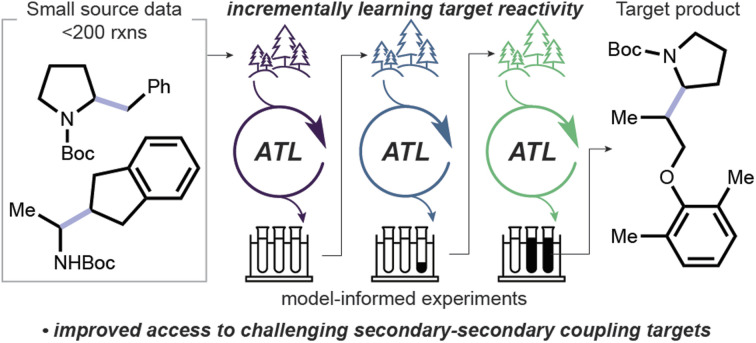

## Introduction

**Fig. 1:**
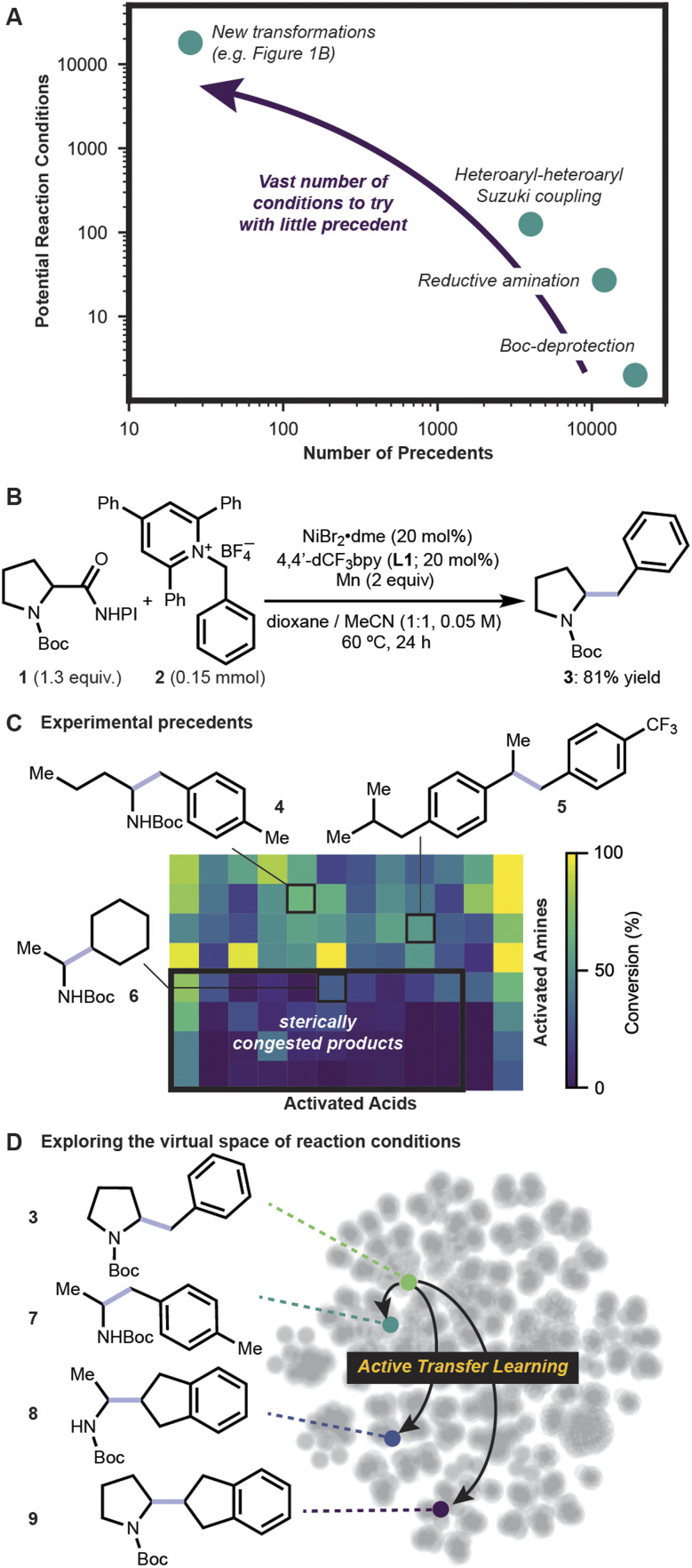
(A) Representative challenges of reaction condition selection. (B) Reductive amine-acid coupling that forms C(sp^3^)–C(sp^3^) bond.^31^ (C) Previous substrate scope screen reveals sterically congested products as a challenge. (D) ATL was used to explore the reaction condition space, identifying modifications for increasingly challenging substrate pairs. NHPI: *N*-hydroxyphthalimide.

Reaction condition selection is a fundamental task in experimental chemical synthesis. For simple, robust reactions with multiple precedents (*e.g.*, Boc deprotection), one of a few well-known reaction conditions will often give satisfactory results (Fig. 1A). Reactions with more complex recipes, like Suzuki coupling, make it more difficult to prioritize reaction conditions, as many conditions exist and no single condition is ideal for every substrate pair.^1–3^ For new reaction methods that have not yet been generalized, all combinations of analogous reagents are potentially viable candidates to promote coupling of distinct substrate pairs. Navigating such a vast space of reaction conditions requires substantial time and resources, so methods that streamline this exploration are needed.^4,5^

Reaction condition exploration with challenging substrates could be constructively guided by machine learning, which has recently shown promise in quantitative predictions of chemical reaction outcomes.^6–19^ Conventional algorithms, however, fall short in regions with sparse data. Therefore, adaptive learning methods which refine predictivity through iterative experimentation have emerged.^20–24^ For instance, Bayesian optimization (BO) has become popular for optimizing the reaction of specific substrate pairs (see SI Section 2 for a survey of recent efforts in this area). However, currently, BO methods that leverage previously collected datasets, which may be informative, remain rare,^25^ presenting a need to develop new strategies.

Accordingly, we recently proposed active transfer learning (ATL), which merges active learning and transfer learning.^26^ ATL starts by transferring^27,28^ a source model trained on prior data to identify promising regions in the target space for exploration. The active learning^29^ step of ATL refines this model by iteratively evaluating reactions it deems most important to improving yield, enhancing the understanding of the reactivity landscape beyond the source model. Our previous application of ATL investigated a dataset of Pd-catalyzed cross-couplings with various classes of nitrogen nucleophiles. The combination of relevant knowledge transferred from a nearby reactivity domain through the source model and its iterative refinement was shown to identify viable reaction conditions for target nucleophiles faster than either learning strategy—active or transfer—alone. Key to ATL's efficiency was exploration within a focused region in the reaction condition space where the most impactful reagent, the catalyst, was prioritized in the source dataset. Based on this finding, the current work prospectively applies ATL on a recently developed amine-acid coupling, which includes a large unexplored space of plausible reaction conditions, to couple challenging substrate pairs.

Cross-coupling reactions between amines and acids,^30–35^ which are the two commercially available building blocks with the broadest diversity,^36–39^ are a promising class of transformations to complement the classic amide coupling.^40–42^ One example is the nickel-catalyzed C(sp^3^)–C(sp^3^) cross-coupling between activated amines and carboxylic acids (Fig. 1B) which provides access to the most prevalent bond among organic compounds: the C–C bond.^31^ Under our previous best reaction condition – which used NiBr_2_·dme as precatalyst and 4,4′-bis(trifluoromethyl)-2,2′-bipyridine (L1) as ligand with manganese as the reductant – diverse pairings of 8 activated amines and 12 activated acids showed >30% conversion for half of the 96 desired products,^31^ yet challenging sterically congested substrates often gave little or no product (Fig. 1C). To identify improved reaction conditions for challenging substrate pairs where at least one coupling site is a secondary sp^3^-carbon atom, ATL was prospectively applied, building on previous optimization data for formation of 3 (Fig. 1D).

## Results

**Fig. 2:**
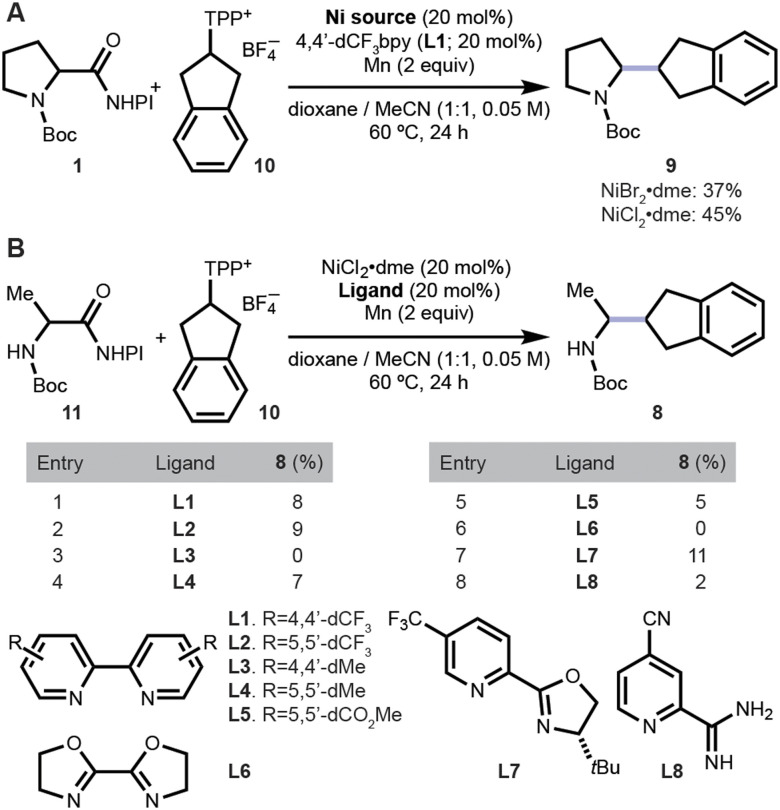
Initial efforts to use activated 2-aminoindane 10 as a coupling partner. (A) Isolated yields of reactions conducted at 0.15 mmol scale. (B) Assay yields determined by ultrahigh performance liquid chromatography-mass spectrometry (UPLC-MS) of 0.01 mmol scale reactions. NHPI: *N*-hydroxyphthalimide. TPP^+^: 2,4,6-triphenylpyridinium.

Before turning to ATL, preliminary experiments were conducted to understand the reactivity of sterically hindered amines. Initially, we attempted to forge a secondary–secondary alkyl bond by coupling 1 with an indan-2-yl pyridinium salt (10) under 12 reaction conditions rationally selected (no ATL) by varying ligands and precatalysts. The reaction condition identified with the highest assay yield was repeated at 0.15 mmol scale, confirming that a switch in nickel precatalyst counterion from Br to Cl while using previously optimal ligand L1, improved the isolated yield from 37% to 45% yield (Fig. 2A).

Subsequent experiments with NiCl_2_·dme that surveyed eight ligands to couple *N*-Boc-alanine-derived substrate 11 with 10 were low yielding (Fig. 2B). The low yields were hypothesized to be caused by differences in the rate of formation or the stability of resulting radicals following deamination of 2 and 10 and decarboxylation of 1 and 11. Therefore, additives and new solvents were also considered necessary to further improve the outcome.

**Fig. 3:**
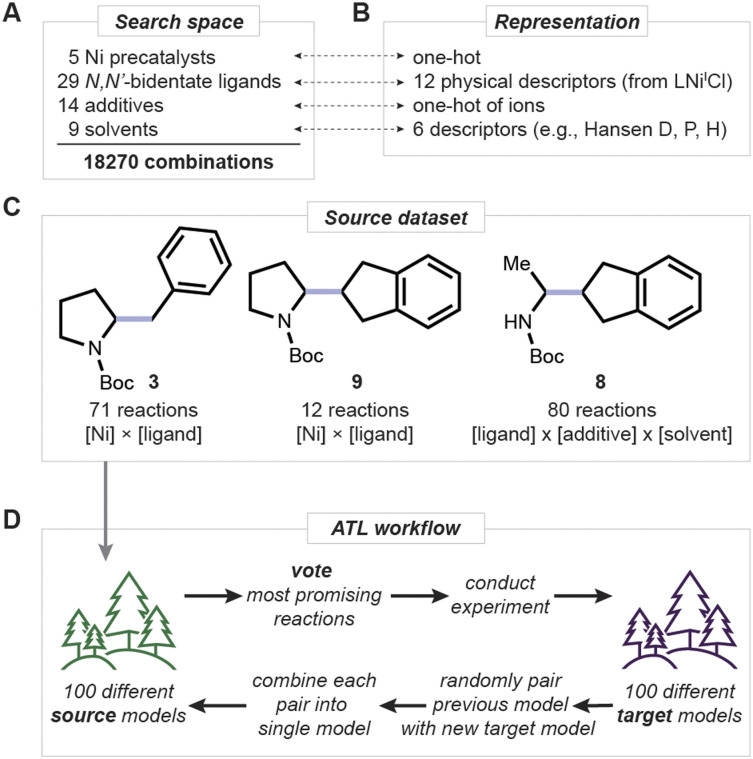
Overview of ATL. (A) Reaction condition search space. (B) Representation of each reagent class. (C) Source dataset structure uses 3, 9, and 8 as products. (D) Schematic description of one ATL iteration.

Accordingly, a list of additives known to impact decarboxylation,^43–45^ [*e.g.*, trimethylsilyl chloride (TMSCl) and NaI] and deamination^46^ (MgCl_2_, Zn, Li and tetrabutylammonium salts) in nickel-catalyzed cross-electrophile couplings^30,47^ was curated. The salt cations and anions were treated independently and mix-and-matched to finalize the list of 14 additives to consider. Additionally, nine solvents that are either commonly used (DMA, NMP) for relevant transformations or ethereal solvents were selected as candidates. Along with five precatalysts and 29 *N*,*N*′-bidentate ligands available in our laboratory, a combined search space of 18 270 reagent combinations was defined (Fig. 3A). To winnow these possibilities to those most likely to improve yields, ATL was applied. Particularly, a practical scenario with a short timeline was adopted, targeting just three iterations of ATL. This was viewed as a reasonable benchmark timeframe that could meaningfully impact a chemistry discovery program.

Before applying ATL, 72 additional reactions of form 11 + 10 → 8 were performed experimentally, sampling ligand, additive and solvent combinations since the newly introduced variables (additives and solvents) were not systematically explored in the previous study. These reactions were carried out using three sets of high-throughput experiments, designed using simple rules. Ligand-additive pairs that worked well in one set were kept for the next round, while we continued to test different solvents and additives. In the later stages of this initial testing, we aimed to both improve reaction performance and understand patterns of reactivity. Reactions conducted up to this point with variations in all reagent classes make the combined dataset suitable as a source for ATL (Fig. 3C).

To initiate ATL, source reactions were represented by concatenating vectors of physical descriptors for substrates, ligands, and solvents, with one-hot encoded nickel precatalysts and additive ions (Fig. 3B, see SI Section 5 for details). The source model, based on random forest classifiers, was then trained to be simple (depth one) for initial transferability and better adaptability, to select initial experiments in the target space (Fig. 3D; see pages S19, S20 and S46 for further details on model hyperparameters). Although using regressors is a viable alternative, predicting whether a reaction condition improves outcome (a classification) is preferrable for a small number of experimental iterations. As such, we continue to use classifiers as we did in our previous study.^26^

However, experiments recommended can vary across different models due to the randomness involved in random forests. To reduce the uncertainty involved in experiment selection, 100 different source models were trained. Then, each model voted for *N* reactions (with an ensemble of 100 source models, the set of reaction conditions suggested by using different *N* values does not vary significantly, see Tables S5 and S15; the voting scheme does not necessarily lead to better model performance) with highest predicted probabilities to improve reaction outcome (*i.e.*, greedy selection; which we previously found to be more efficient than other strategies that involve uncertainty^26^). The reactions with the most votes were conducted as the next batch of experiments. Subsequently, 100 new models were trained on the newly collected reaction data and combined into the previous models, updating the overall knowledge of the target reactivity. The process of ranking, experiment, and model update corresponds to one iteration of the ATL protocol.

**Fig. 4:**
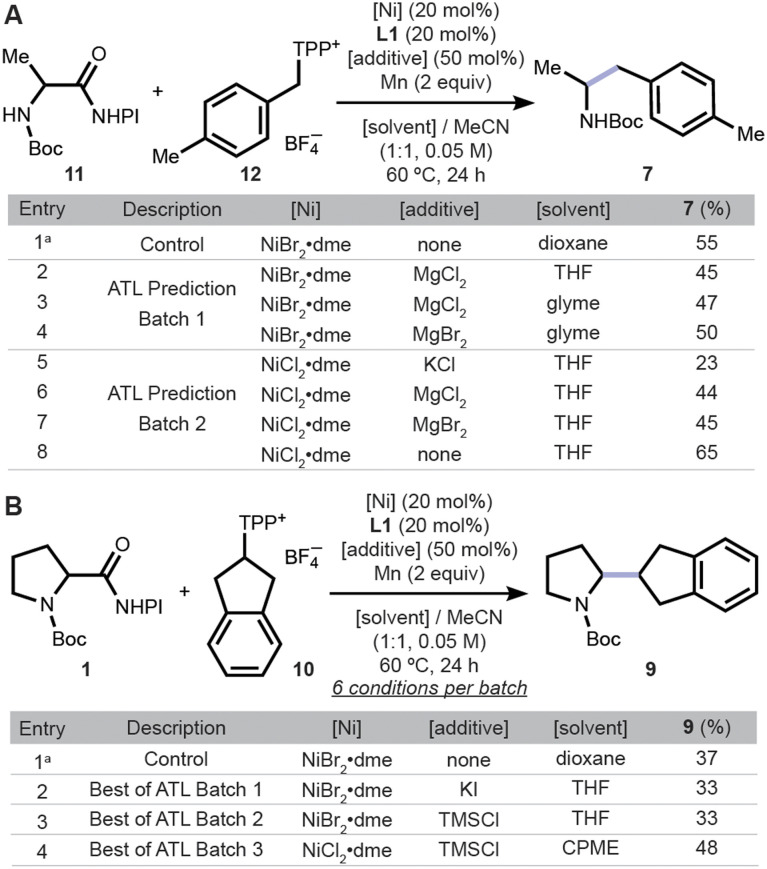
Application of ATL for (A) coupling 11 with 12 and (B) coupling 1 with 10. Reactions were conducted at 0.05 mmol scale and their assay yields are shown, except those in entry 1. ^*a*^Isolated yield from reaction conducted at 0.15 mmol scale. NHPI: *N*-hydroxyphthalimide. TPP^+^: 2,4,6-triphenylpyridinium.

For an initial case study, 11 and 4-methylbenzyl pyridinium salt 12 were selected as target substrates. In principle, the source model's knowledge of reaction conditions for 1 and 2 will be useful due to the structural and electronic similarity to 11 and 12, respectively. Under the previously reported condition, 11 and 12 react to give 8 in 55% yield (Fig. 4A entry 1), supporting the hypothesis that the reactions are similar. The transferred source model recommended experiments that all included the ligand L1, perhaps due to this ligand being used in the largest number of successful reactions in the source dataset (Fig. S6). For the remaining conditions, magnesium additives paired with two different solvents were suggested, possibly due to their positive impact on forming 8 in the source dataset (entries 2–4). However, none of the three reactions in the first iteration gave higher assay yield. Therefore, trees that suggested those conditions were removed from the source random forests to refine its predictive ability. The updated model chose three additives (entries 5–7), and a control with no additives was also conducted (entry 8). The latter gave a higher assay yield (65%) than the initial yield of 55% (entry 1). The efficacy of using no additive may be due to the high chemical similarity of the target substrates to 1 and 2: our previous report showed that the latter substrates did not benefit from additives under the optimized condition.^31^

Next, the coupling of cyclic substrates 1 and 10 (Fig. 4B) was revisited in an attempt to form C–C bonds between two secondary alkyl carbons.^48–50^ Few such cross-coupling reactions are known, due to the challenging sterics, yet would generate complex and highly desirable sp^3^-rich products. The first batch of six reaction conditions was determined using the source model, which suggested conditions with varying additive anions and solvents. In the second iteration of ATL the models suggested varying the additives while using the previous best solvent, THF. The best assay yield observed across the two iterations was 33% (entries 2–3). The third iteration of ATL fixed the additive to TMSCl, the best from the second round, and queried combinations of nickel precatalysts and solvent. As a result, three reaction conditions returned higher assay yields than the previously reported optimized condition (48% being the highest, entry 4). With the help of the source dataset, ATL suggested changes to three reaction components, which increased the yield from 37% to 48% within three iterations.

**Fig. 5:**
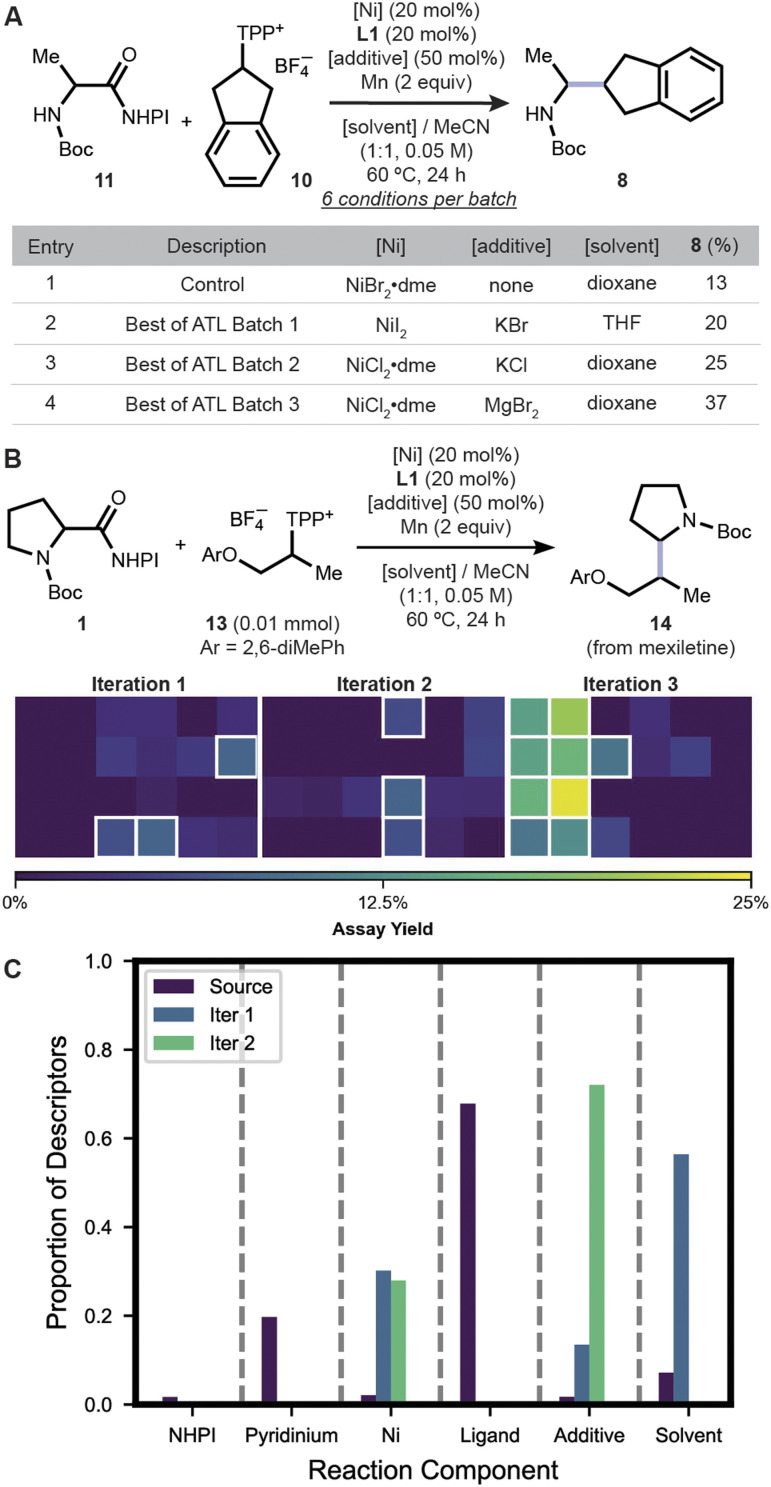
(A) Best results from each of the three batches of six reactions suggested by ATL. Assay yields of reactions conducted at 0.05 mmol scale are shown. (B) Assay yields of 14 from each ATL iteration performed using HTE. (C) Portion of descriptors used by models after each iteration to make a prediction. NHPI: *N*-hydroxyphthalimide. TPP^+^: 2,4,6-triphenylpyridinium.

Substrate pairs with one cyclic and one acyclic secondary moiety were considered next. The previous condition gave a 13% assay yield of 8 from 11 and 10 (Fig. 5A, entry 1; *c.f.*, Fig. 2B where NiCl_2_·dme and L1 gave an 8% assay yield) clearly shows that this is a challenging substrate pair. To improve the reaction, the ATL model first suggested coupling of 11 with 10 using different nickel precatalysts and solvents. With NiI_2_ and NiCl_2_·dme providing assay yields higher than 13%, the next two batches of reactions proposed by ATL continued to examine the two precatalysts with various additives, mostly using dioxane as co-solvent (Tables S21–S22). Enhanced yields were observed at all iterations, arriving at a 37% assay yield when NiCl_2_·dme was used with MgBr_2_ as additive in a mixture of acetonitrile and dioxane co-solvents. To provide a comparison to baseline methods, the total 98 reactions for this substrate pair were retrospectively analyzed, showing ATL to be the best model (see Fig. S20).

As a final test case for ATL was examined using 1-(2,6-dimethylphenoxy)propyl-2-pyridinium salt (13, derived from the drug mexiletine), which has a vicinal ethereal oxygen that can impact radical stability, act as a chelator, and has higher steric bulk than other pyridinium salts studied. These factors make the coupling with 1 particularly challenging – the previous reaction condition returned 7% assay yield. High-throughput experimentation (HTE)^51–55^ studies were conducted in 24-well reactor blocks to survey the reaction space. While other numbers of reactions could reasonably be considered from a theoretical standpoint, the practicality of running experimental studies in standard HTE labware makes batches of 6 or 24 reactions highly practical.^56^ Initially, all three reagent components except the ligand were surveyed but returned minimal improvements over the 7% benchmark (Fig. 5B, iteration 1). Subsequently, using precatalysts that were successful in the first iteration, ATL focused on identifying suitable additives. In fact, the ATL models queried nearly all additive candidates (Table S11). This illustrates the distinctive reactivity of 1 with the drug-derived substrate 13, since it implies the difficulty of prioritizing a few additives among the additives investigated in the source dataset. More sophisticated featurization of additives may be useful for similar situations in future applications.^57^ Nonetheless, no significant improvement was observed (Fig. 5B, iteration 2). In the last iteration, the left half of the plate surveyed combinations of nickel sources and solvents using KBr as additive. Among them, nine entries returned greater than 10% assay yields, including yields greater than 20% in three wells (Fig. 5B, iteration 3). Further tuning of stoichiometries at 0.15 mmol scale improved the yield to 43% (Table S14), demonstrating ATL's utility in the early stages of reaction development when partnered with downstream optimization.

Lastly, to understand the learning behaviors of ATL, the models in Fig. 5B were analyzed in terms of their use of chemical descriptors (Fig. 5C; see SI Fig. S19–S21 for Shapley value analyses). The source model makes predictions primarily based on ligand features (purple bars) which suggests their importance to reactivity and also explains why L1 was recommended in all experiments. The target models were therefore relied upon to delineate other reagent components. The first target models learned how the solvent and nickel source affect reactivity (blue bars). Similarly, the next round of target models supplemented understanding of how additives impact reaction outcome (green bars). The three models, when combined, make predictions based on descriptors spanning all reagent classes (similar learning behavior is observed for case studies 11 + 10 and 1 + 10; see Fig. S16 and S18). This is a possible explanation for the consistent observation of highest yields in the third iteration. Accordingly, the ‘(number of variables – 1)-th’ iteration could be a reasonable rule of thumb to estimate when meaningful enhancements may appear using ATL.

## Discussion

Reaction optimization involves iterative experimentation that leverages prior knowledge as well as knowledge gained from each batch of experiments. Our formulation of ATL is a new technique that can be used for this purpose, operates on small data, and iteratively refines the models' knowledge. In this sense, ATL is related to BO methods (see SI Section 2 for examples of their prospective applications). However, ATL has unique distinctions that contribute to the field of machine learning for reaction improvement and thus merits further evaluation.

One significant advantage of ATL is its incorporation of source model transfer to use previously collected reactions from a different domain that are relevant to the target reactions of interest. The transfer does not simply borrow the source model and use it in the target domain because the two domains are not similar enough for the optimal source model to effectively prioritize reaction conditions for the target. To transfer only the most relevant information across this reactivity gap, simplified source models are used (the importance of model simplification has been studied in ref. 26). This connection between reactivity domains allows iterative application of ATL to new targets, where accumulated reaction data helps to expand reaction scope beyond what could be accomplished without support from statistical models. More importantly, in our case studies, the source model narrowed down the ligand search space from 29 to one, or from 18 270 to 630 potential reaction conditions, saving considerable experimental effort.

Another useful feature of ATL is the observed consistency in the iteration in which the reaction condition with highest improvement is identified. The consistency of predictions arises from the model's adaption to the target reactivity domain, where improved reaction conditions are more likely to be suggested once all reagent types have been studied. This provides the practitioner a sense of how long the campaign would take for one target substrate. Simultaneously, the consistency acts as a stopping criterion that prevents superfluous experiments.

Nonetheless, ATL possesses inherent limitations. In order for the transferred source model to be useful, the source reactions need to be ‘relevant’ to the target.^4^ Failure to secure effective transfer may result in decreased model performance, leading to wasted resources. Although there currently is no quantitative method for judging their relevance,^58^ an expert chemist's intuition on the plausibility of applying source reactivity information to the target can be an effective qualitative measure. The other limitation roots from ATL's current use of classifiers. As the model is trained to classify whether a new reaction condition will have higher yield than the previously optimized condition, distinguishing those with significant yield benefit from those that give minimal increment is difficult. Dynamically increasing the classification threshold or incorporating regressors are plausible strategies that may address this issue.

## Conclusion

In conclusion, ATL was prospectively applied to expand the applicability of the nickel-catalyzed amine-acid C(sp^3^)–C(sp^3^) coupling of challenging sterically-congested secondary substrates.^31^ Improved results were consistently obtained within three rounds of experiments for four substrate pairs where one or both coupling sites included a secondary carbon, accessing complex sp^3^-rich products. These amine-acid couplings complement the amide coupling of amines and acids. An ether-containing substrate derived from the drug mexiletine (13) initially coupled in 7% yield was obtained in 43% yield following three rounds of ATL, with subsequent optimization of stoichiometries. In discovery settings where chemical knowledge is limited and the full combinatorial set of discrete reagents is inaccessible, ATL is a powerful tool for initial optimization for challenging substate pairs, particularly when relevant prior data is available.

## Conflicts of interest

The authors declare the following competing financial interest(s): the Cernak Lab has received research funding or in-kind donations from MilliporeSigma, Relay Therapeutics, Janssen Therapeutics, SPT Labtech, Iambic Therapeutics and Merck & Co., Inc. T. C. is a co-Founder and equity holder of Iambic Therapeutics.

## Supplementary Material

DD-004-D5DD00309A-s001

## Data Availability

Code and reaction data used in this study is available at https://github.com/cernak-lab/ATL_EXP which is associated with https://doi.org/10.5281/zenodo.17314341. Supplementary information: descriptions of the dataset, modeling procedure and additional data that support results of this work. See DOI: https://doi.org/10.1039/d5dd00309a.
