# Supplementary material for: Prospective active transfer learning on the formal coupling of amines and carboxylic acids to form secondary alkyl bonds
Source: Digit Discov. 2025 Nov 7;4(12):3693–700. doi: 10.1039/d5dd00309a (PMC12593407; doi:10.1039/d5dd00309a)
Supplement: DD-004-D5DD00309A-s001 [file DD-004-D5DD00309A-s001.pdf]

Supplementary Information (SI) for Digital Discovery.
This journal is © The Royal Society of Chemistry 2025

Supporting Information for

Prospective Active Transfer Learning on the Formal Coupling of Amines and Carboxylic Acids to Form Secondary Alkyl Bonds

Eunjae Shim[1], Ambuj Tewari[2,3], Paul M. Zimmerman[1,*], Tim Cernak[1,4,*]

1. Department of Chemistry, University of Michigan, Ann Arbor, MI
2. Department of Statistics, University of Michigan, Ann Arbor, MI
3. Department of Electrical Engineering and Computer Science, University of Michigan, Ann Arbor, MI
4. Department of Medicinal Chemistry, University of Michigan, Ann Arbor, MI

Email: paulzim@umich.edu, tcernak@med.umich.edu

Table of Contents

1) Data Sources for Figure 1A S-01
2) Prospective active learning / Bayesian optimization precedents S-02
3) General Information S-05
4) General Procedure for HTE screens S-07
5) Source Dataset
 5-1) Curation S-08
 5-2) Structure S-16
 5-3) Featurization S-17
 5-4) Reaction condition candidates S-19
 5-5) Preliminary Modeling S-19
6) Case Study 1 – **11** + **12** → **7** S-21
7) Case Study 2 – **1** + **13** → **14** S-24
8) Case Study 3 – **1** + **10** → **9** S-35
9) Case Study 4 – **11** + **10** → **8** S-41
10) Retrospective analysis S-46
11) Model interpretation through Shapley value analysis S-48
12) Synthetic Procedures S-51
13) References S-60
14) Spectra S-62

1) Data Sources for Figure 1A

Figure 1A describes how combining a few reagents that are most popularly used for well-established reactions such as Suzuki coupling, reductive amination and Boc-deprotection covers thousands of precedents. This is in stark contrast to new transformations where over tens of thousands of viable reaction conditions, analogous to the reported condition, can be conceived. The plot was generated from the data of the three reactions, collected from the Reaxys database (reaxys.com).

Boc-deprotection reactions were accessed by drawing the query R–NHBoc → R-NH_2. This initial search resulted in over 170K precedents.[1] Limiting the results with the most popular reaction condition (TFA in DCM) along with the “yield>65%” and “single step reactions only” filters, 19115 reactions remained.

Precedents of reductive amination were accessed using the query, R–NH_2 + R–CHO → R–NH–CH_2–R where the hydrogens on the amine and aldehyde were explicitly drawn out. This returned approximately 48K results.[2] The reactions were further filtered to those that employ three most popular reductants {$NaBH_4$, $NaBH_3CN$, $NaB(OAc)_3H$} and solvents {MeOH, DCM, THF}, along with the “single step reactions only” filter. 23692 reactions remained after this process. The reactions were further filtered to include only the top three most popular reductants and solvents, finally returning 12070 reactions.

Suzuki couplings involving two heteroaryl groups were searched. In particular, using the group generics HAR, the following reactant query was specified: HAR–Br + HAR–BOH_2.[3] The product was not specified as Reaxys does not allow two generics connected by a bond as product. Then, base entries (e.g., different names for the same chemical) were manually processed and Pd source was split from the ligand. As a result, {$Pd(PPh_3)_4$, $Pd(dppf)Cl_2$, $Pd(PPh_3)_2Cl_2$, $Pd(OAc)_2$, $Pd_2(dba)_3$}, {PPh_3, dppf, SPhos, P*t*Bu_3, P(*o*-tol)$_3$} and {K_2CO_3, Na_2CO_3, Cs_2CO_3, K_3PO_4, $NaHCO_3$} were shown to be the five most popular Pd sources, ligands and bases, respectively. The number of reactions that employ the reagents from these three sets was 4022.

2) Prospective active learning / Bayesian optimization precedents

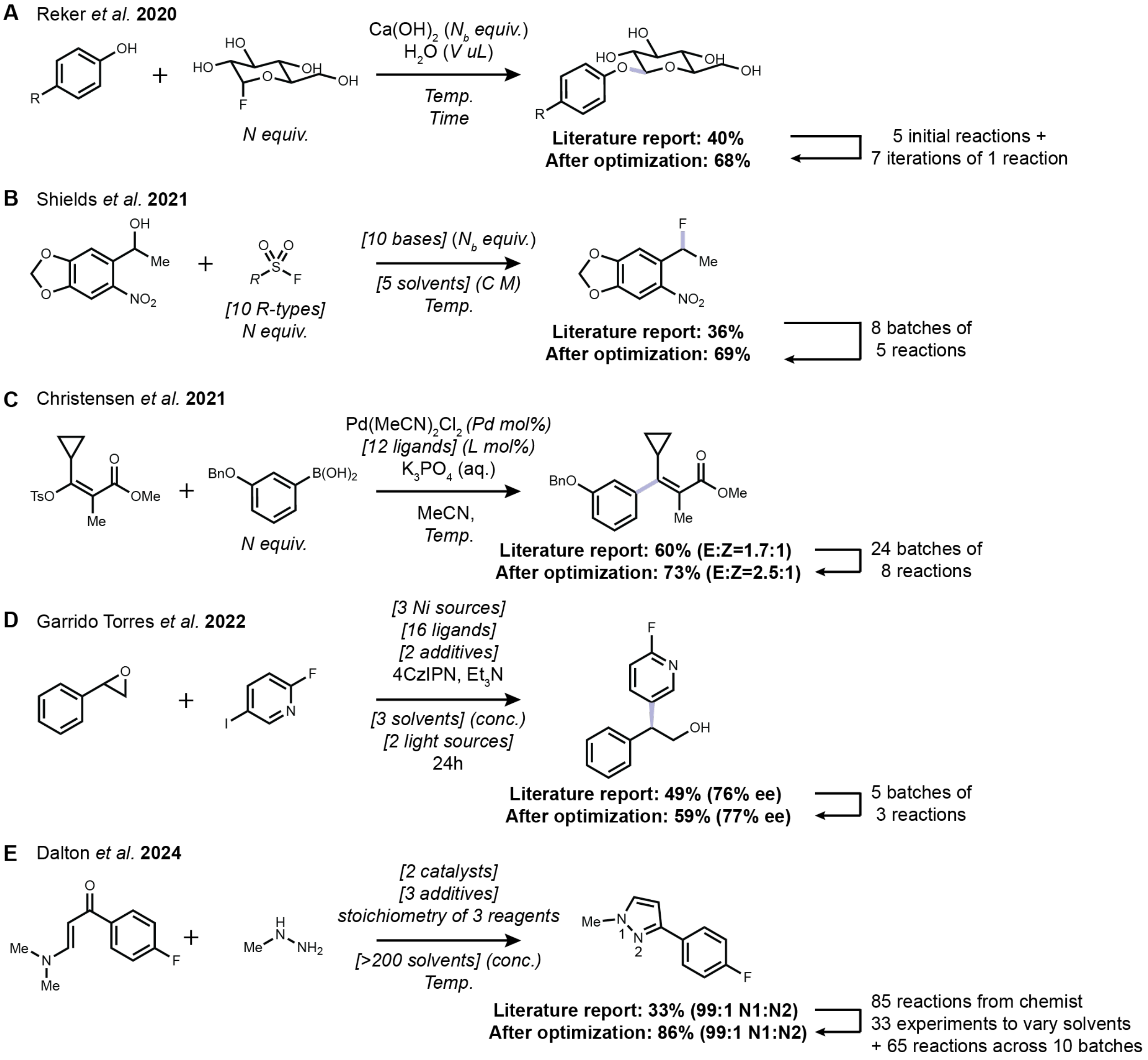

Figure S1. Representative prospective applications of various active learning or Bayesian optimization models on organic transformations. Categorical variables and the number of candidates considered are marked in brackets. Examples with the lowest yield obtained with the previously optimized condition, as reported in the literature, are shown. These values, prior to 'tailored' optimization, present a challenging case since the reported reaction conditions worked well on different substrates, but not as well on the target being optimized.

Recent years have seen the development of active learning and Bayesian optimization (BO) algorithms for improving reaction conditions of known transformations. While these tools are typically developed and tested using existing experimental results, they have been evaluated in a prospective manner for a handful of reaction types. Figure S1 shows

the results of prospective active learning or BO on yield and selectivity from five works. This section of the SI discusses these cases in more detail, in order to set a broader context for the ATL studies of the main text.

In Ref 1 (Figure S1A), numerous types of reactions were subject to a random forest regressor active learning platform.[1] Mostly continuous variables were optimized, finding combinations of stoichiometry, temperature, and reaction time from a search space on the order of magnitude of 10^4. In a couple cases, categorical variables were considered as well by encoding them as one-hot vectors. As shown in Figure S1A, the most challenging case-study was an *O*-glycosylation reaction that returned 40% in the literature report. With 7 iterations, a reaction condition that could achieve 68% yield was identified.

The prospective evaluation of a BO algorithm in Ref 2 (Figure S1B) sought to optimize a deoxyfluorination reaction.[2] Although the search space altogether contained >300,000 possibilities, the number of combinations of reagents and solvents was 500 (i.e., the search space was dominated by continuous variables). The previously reported reaction condition gave 36% yield. After 8 batches of 5 reactions, the best optimized condition gave 69% yield. Two more batches of 5 reactions were conducted, each including a distinct reaction condition that achieved 69% and 68%, respectively.

By combining optimization algorithms that act upon categorical and continuous variables separately, ref 3 aimed to optimize the yield and E/Z ratio of a stereoselective Suzuki coupling (Figure S1C).[3] Like Figure S1A and S1B, the search space was mostly determined by four continuous variables with only one categorical variable (12 ligand choices). The reaction that was optimized was previously shown to give 60% yield with 1.7:1 E/Z ratio. With their algorithm, after running 8 batches of 24 reactions, a condition that achieves 73% yield with 2.5:1 E/Z ratio was identified.

In another example, a BO framework was extended to perform multi-objective optimization in Ref 4 (Figure S1D).[4] Unlike the above examples, the search space for the nickel-catalyzed ring-opening of epoxides with aryl iodides was mostly characterized by categorical variables (i.e., reagent identities) spanning >500 possibilities. One test study used a substrate pair with an initial 49% yield and 76% ee. At the fifth iteration (including the initializing three reactions) of three experiments, a condition that returned 59% yield

and 77% ee was identified. The exploration was conducted for three more rounds although higher ee values were not observed.

Finally, Knorr pyrazole condensation was optimized using BO methods (Figure S1E).[5] An expert chemist began this study by hand selecting 118 reactions for an initial dataset before turning to BO. Then using different BO strategies, a total of 65 reactions were conducted across 10 batches (5 batches of 5 reactions followed by 5 batches of 8 reactions). As a result, incremental enhancements led to a change from 33% conversion to 86% conversion while retaining 99:1 N1:N2 regioselectivity.

In summary, reaction optimization can be achieved (with varying degree of yield and selectivity enhancements) by conducting a range of 12 to 192 experiments spread across 5 to 24 iterations. In a couple cases, the exploration was continued a few iterations after the best results have been identified as there is no stopping criteria for these algorithms. Moreover, the improvements were typically a result of modifying continuous variables, with the largest number of possible reagent combinations being below 1,000.

In contrast, ATL, unlike BO, is not primarily designed for obtaining the highest yield possible (i.e., optimization). By using a classifier that predicts whether reaction outcome would be improved over the previous best condition, combined with the leverage of previously collected data, ATL's goal is to identify better sets of reagents quickly. Here, ATL focuses on exploring through a total of >18,000 reagent combination candidates efficiently, within 3 iterations (after 'previously collected data' is included) below a total of 100 reactions. These features posit ATL as a potential complementary tool for further optimization by significantly narrowing the search space of reagent combinations to explore. On the other hand, this study tackles particularly challenging test cases (products **8** and **14**) which give approximately 10% yields using the previously reported reaction condition. In all, this ATL study expands the current body of prospective work, differing in both problem setting and difficulty of the reactions attempted.

3) General Information

All reactions were conducted in oven- or flame-dried glassware under an atmosphere of nitrogen unless stated otherwise. Reactions were set up in an MBraun LABmaster Pro Glove Box (H_2O level <0.1 ppm, O_2 level <0.1 ppm), or using standard Schlenk technique with a glass vacuum manifold connected to an inlet of dry nitrogen gas. Tetrahydrofuran, acetonitrile and dichloromethane were purified using an MBraun SPS solvent purification system by purging with nitrogen, and then passing the solvent through a column of activated alumina. 1,4-Dioxane, cyclopentyl methyl ether (CPME) and other solvents were purchased as the anhydrous solvents and used as received. Reagents were purchased from Sigma Aldrich, Thermo Fischer Scientific, Alfa Aesar, Oakwood Chemical, or TCI Chemical. All chemicals were used as received. Glass 1-dram (Fisherbrand^{TM} parts No. 03-330-21B) 2-dram vials (Fisherbrand^{TM} parts No. 03-339-21D) were used as reaction vessels, fitted with standard screwcaps (#03-452-225 or #03-452-300) or with Teflon-coated silicone septa (#CG-4910-02), and magnetic stir bars (Fisher Scientific #14-513-93 or #14-513-65; stirbars.com #SBM-0803-MIC or #SBM-1003-MIC).

^{1}H NMR spectra were recorded on a Varian MR-500 MHz, Varian MR-400 MHz or Bruker Avance Neo 500 MHz spectrometer. Chemical shifts are reported in parts per million (ppm) and the spectra are calibrated to the resonance resulting from incomplete deuteration of the solvent ($CDCl_3$: 7.26 ppm). ^{13}C NMR spectra were recorded on the same spectrometers with complete proton decoupling. Chemical shifts are reported in ppm with the solvent resonance as the internal standard ($^{13}CDCl_3$: 77.16 ppm, t). Data are reported using the abbreviations: s = singlet, d = doublet, t = triplet, q = quartet, m = multiplet, br = broad. Coupling constant(s) are reported in Hz. ^{13}C signals are singlets unless otherwise stated.

High resolution mass spectrometry data (HRMS) were obtained on an Agilent 6230 TOF LC/MS equipped with ESI detector in positive mode and on a Micromass AutoSpec Ultima Magnetic Sector instrument with EI detector in positive mode. Reaction analysis was typically performed by thin-layer chromatography on silica gel or using a Waters I-class ACQUITY UPLC-MS (Waters Corporation, Milford, MA, USA) equipped with in-line

photodiode array detector (PDA), evaporative light scattering detector (ELSD) and QDa mass detector (Both ESI positive and negative ionization modes). Typically, 0.1 μL sample injections were taken from acetonitrile solutions of reaction mixtures or products (~1 mg/mL). A partial loop injection mode was used with the needle placement at 2.0 mm from bottom of the wells and a 0.2 μL air gap at pre-aspiration and post-aspiration. Column used: Waters Cortecs UPLC C18+ column, 2.1 mm × 50 mm with (Waters #186007114) with Waters Cortecs UPLC C18+ VanGuard Pre-column 2.1 mm × 5 mm (Waters #186007125), Mobile Phase A: 0.1 % formic acid in Optima LC/MS-grade water, Mobile Phase B: 0.1% S5 formic acid in Optima LC/MS-grade MeCN. Flow rate: 0.8 mL/min. Column temperature: 45 °C. The PDA sampling rate was 20 points/sec. The QDa detector monitored m/z 150-750 with a scan time of 0.06 seconds and a cone voltage of 30 V. The ELSD had a gain of 750, data rate of 10 pps, time constant “normal” 0.2000 sec, a gas pressure of 40.0 psi, with the nebulizer in cooling mode at 75% power level and the drift tube temperature set to 50 °C. The PDA detector range was between 210 nm – 400 nm with a resolution of 1.2 nm. A two-minute method was used. The method gradients are: 0 min: 0.8 mL/min, 95% 0.1% formic acid in water/5% 0.1% formic acid in acetonitrile; 1.5 min: 0.8 mL/min, 0.1% 0.1% formic acid in water/99.9% 0.1% formic acid in acetonitrile; 1.91 min: 0.8 mL/min, 95% 0.1% formic acid in water/5% 0.1% formic acid in acetonitrile.

Flash chromatography was performed on silica gel (230 – 400 Mesh, Grade 60) under a positive pressure of nitrogen. Thin Layer Chromatography was performed on 25 μm TLC silica gel 60 F254 glass plates purchased from Fisher Scientific (part number: S07876). Visualization was performed using ultraviolet light (254 and 365 nm) and/or potassium permanganate ($KMnO_4$) stain.

All computational analyses can be accessed at:
github.com/cernak-lab/ATL_EXP

4) General procedure for HTE screen preparation

In an inert atmosphere glovebox, reagents were weighed and dissolved or suspended in anhydrous solvent to achieve their listed concentrations in table. Stock solutions of reagents were stirred until either a clear solution or a uniform slurry was achieved. A 24 or 96-well aluminum microvial plate (Analytical Sales & Services cat. no. 25243) was equipped with oven-dried shell vials (Analytical Sales & Services cat. no. 884001) and then moved into the glovebox. Stock solutions were dosed to the appropriate shell vials according to the plate map shown in table using single channel micropipettes. A perylene-coated stir dowel (Analytical Sales & Services cat. no. 13258) was then added to each vial. The microvial plate was sealed, removed from the glove box, and stirred on a tumble stirrer with heating to indicated temperature for planned reaction time in a heating block.

The reactions were quenched by opening the reaction block and adding 100 μL saturated aqueous NaCl solution and 400 μL EtOAc. Reactions were extracted by resealing the plate and shaking manually. From each reaction, a 40 μL aliquot of the quenched reaction mixture was added into a 96-well polypropylene collection plate (Analytical Sales & Services cat. no. 17P687). The solvent was evaporated by blowing nitrogen down on the analytical plate. An acetonitrile solution of caffeine as internal standard (0.05 mg/mL, 800 μL) was added, and mixed by pipetting up and down. The reactions were then analyzed by UPLC-MS. The assay yields were produced by measuring the UV absorbance of desired product relative to the caffeine internal standard with calibration curves shown in Figure S2.

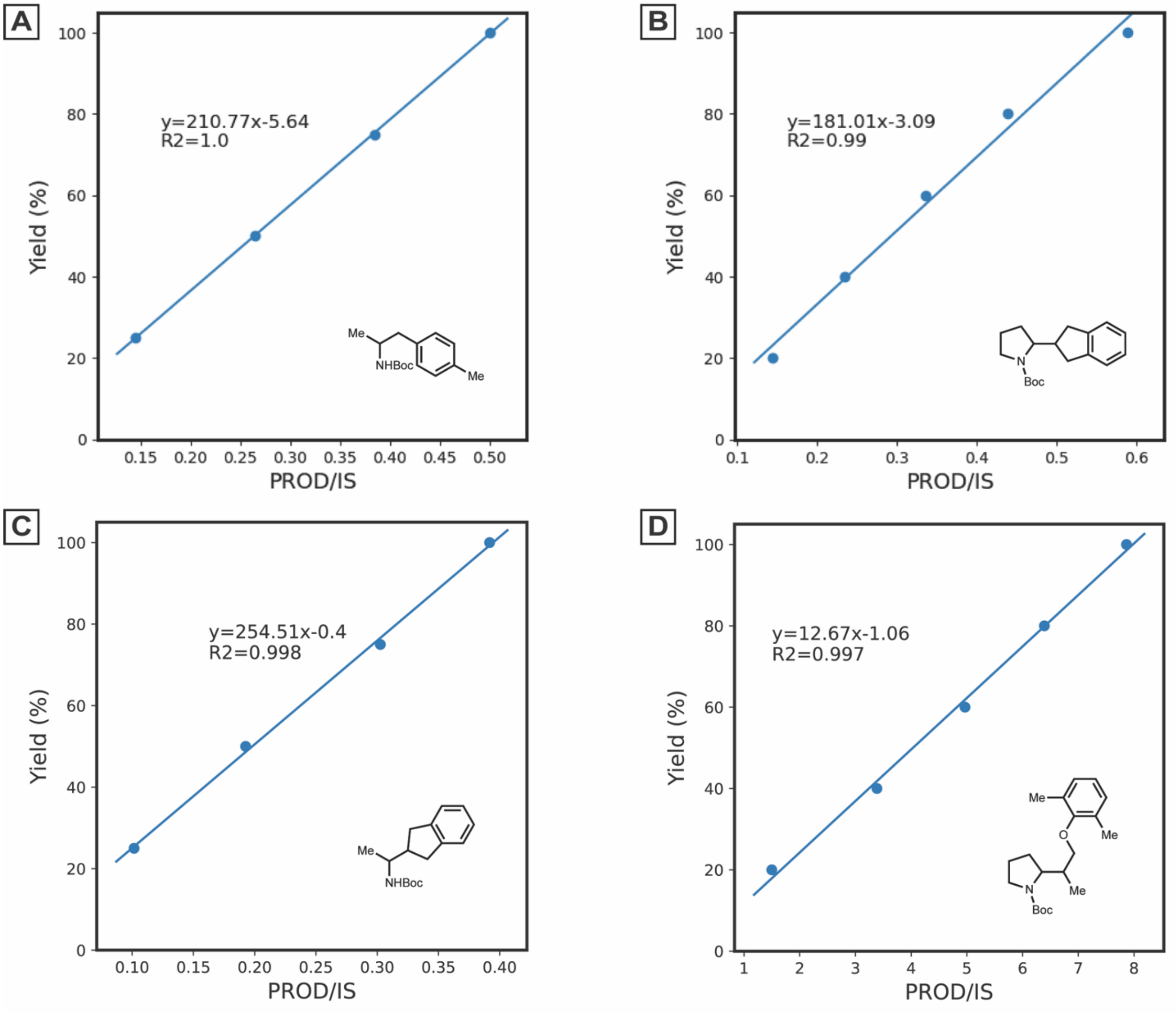

Figure S2. Calibration curves used for target products of the four test studies in the main text.

5) Source Dataset

5-1) Curation

To equip the source model with knowledge across all reaction components, in addition to the reactions of *N*-Boc-proline and benzyl pyridinium salt from the main text's Ref. 8, the following sets of HTE experiments were conducted. Due to consistently poor reactivity of 4'-methoxy- or trifluoromethyl-substituted pyridiniums, only unsubstituted pyridinium substrates were included as the source dataset and further considered in this study.

Screen 1

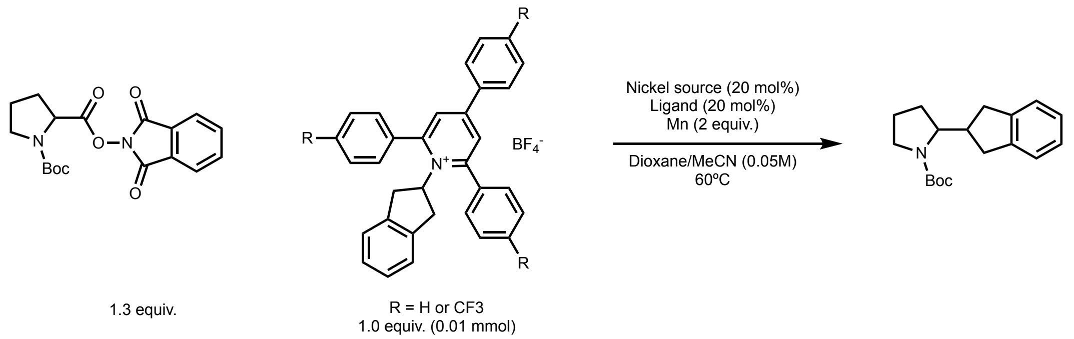

Table S1. Reagents, stock solution concentrations, dosing volumes and well locations in screen 1.

Reagents	Solvent	C_{stock} (M)	V_{dose} (µL)	Wells
BocPro-NHPI	1,4-dioxane	0.52	25	All
Indan pyridinium	1,4-dioxane	0.4	25	A-D1, A-D3, A-D5
Indan 4'CF_3-pyridinium	1,4-dioxane	0.4	25	A-D2, A-D4, A-D6
$NiBr_2$•dme	1,4-dioxane	0.08	25	A-D 1-2
$NiCl_2$•dme	1,4-dioxane	0.08	25	A-D 3-4
$Ni(acac)_2$	1,4-dioxane	0.08	25	A-D 5-6
4,4'-dimethyl-2,2'-bipyridine	1,4-dioxane	0.08	25	A 1-6
5,5'-dimethyl-2,2'-bipyridine	1,4-dioxane	0.08	25	B 1-6
4,4'-bis(trifluoromethyl)-2,2'-bipyridine	1,4-dioxane	0.08	25	C 1-6
5,5'-bis(trifluoromethyl)-2,2'-bipyridine	1,4-dioxane	0.08	25	D 1-6
Mn	MeCN	0.2	100	All

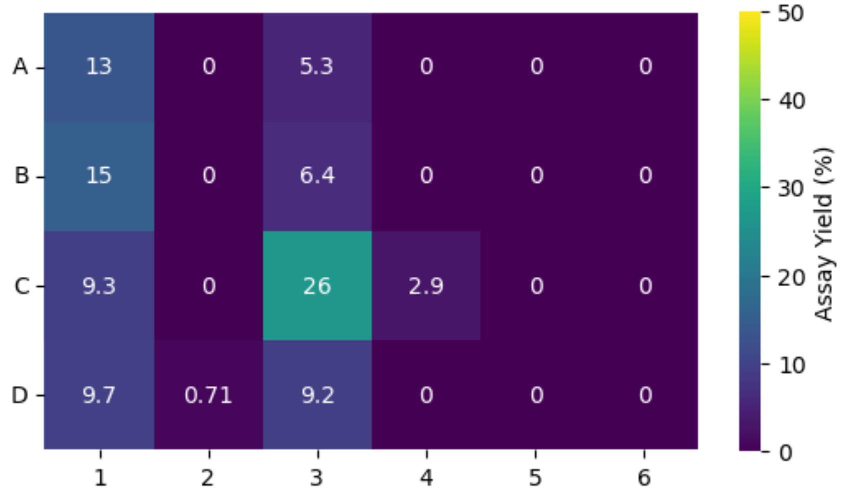

Figure S3. Assay yield heatmap of screen 1 (see Table S1).

Screen 2

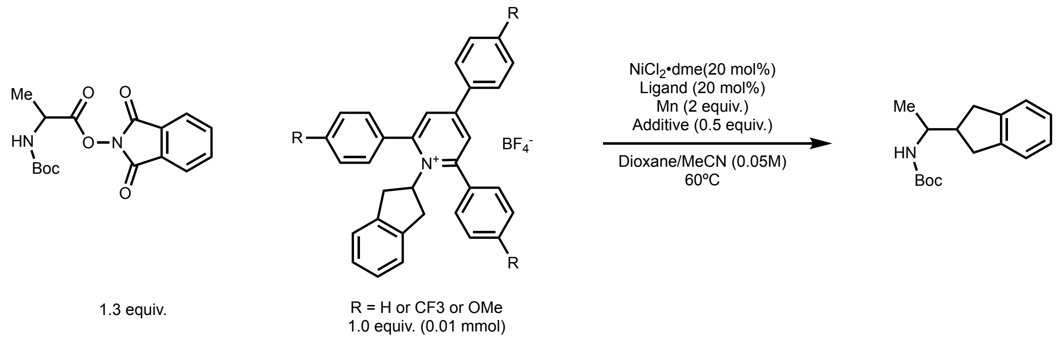

Table S2. Reagents, stock solution concentrations, dosing volumes and well locations in screen 2.

Reagents	Solvent	C_{stock} (M)	V_{dose} (μL)	Wells
BocAla-NHPI	1,4-dioxane	0.52	25	All
Indan 4'OMe-pyridinium	1,4-dioxane	0.4	25	A1-H4
Indan pyridinium	1,4-dioxane	0.4	25	A5-H8
Indan 4'CF_3-pyridinium	1,4-dioxane	0.4	25	A9-H12
$NiCl_2$•dme	1,4-dioxane	0.08	25	All
4,4'-dimethyl-2,2'-bipyridine	1,4-dioxane	0.08	25	A1-A12
5,5'-dimethyl-2,2'-bipyridine	1,4-dioxane	0.08	25	B1-B12
4,4'-bis(trifluoromethyl)-2,2'-bipyridine	1,4-dioxane	0.08	25	C1-C12
5,5'-bis(trifluoromethyl)-2,2'-bipyridine	1,4-dioxane	0.08	25	D1-D12
Diethyl 2,2'-bipyridine-5,5'-dicarboxylate	1,4-dioxane	0.08	25	E1-E12
2,2'-bisoxazole	1,4-dioxane	0.08	25	F1-F12
(*S*)-4-(*tert*-butyl)-2-(5-(trifluoromethyl)pyridin-2-yl)-4,5-dihydrooxazole	1,4-dioxane	0.08	25	G1-G12
(*Z*)-*N'*-cyanopicolinimidamide	1,4-dioxane	0.08	25	H1-H12
Pure solvent	MeCN	n/a	50	A-H 1, 5, 9
NaI	MeCN	0.1	50	A-H 2, 6, 10
$MgCl_2$	MeCN	0.1	50	A-H 3, 7, 11
Succinimide	MeCN	0.1	50	A-H, 4, 8, 12
Mn	MeCN	0.4	50	All

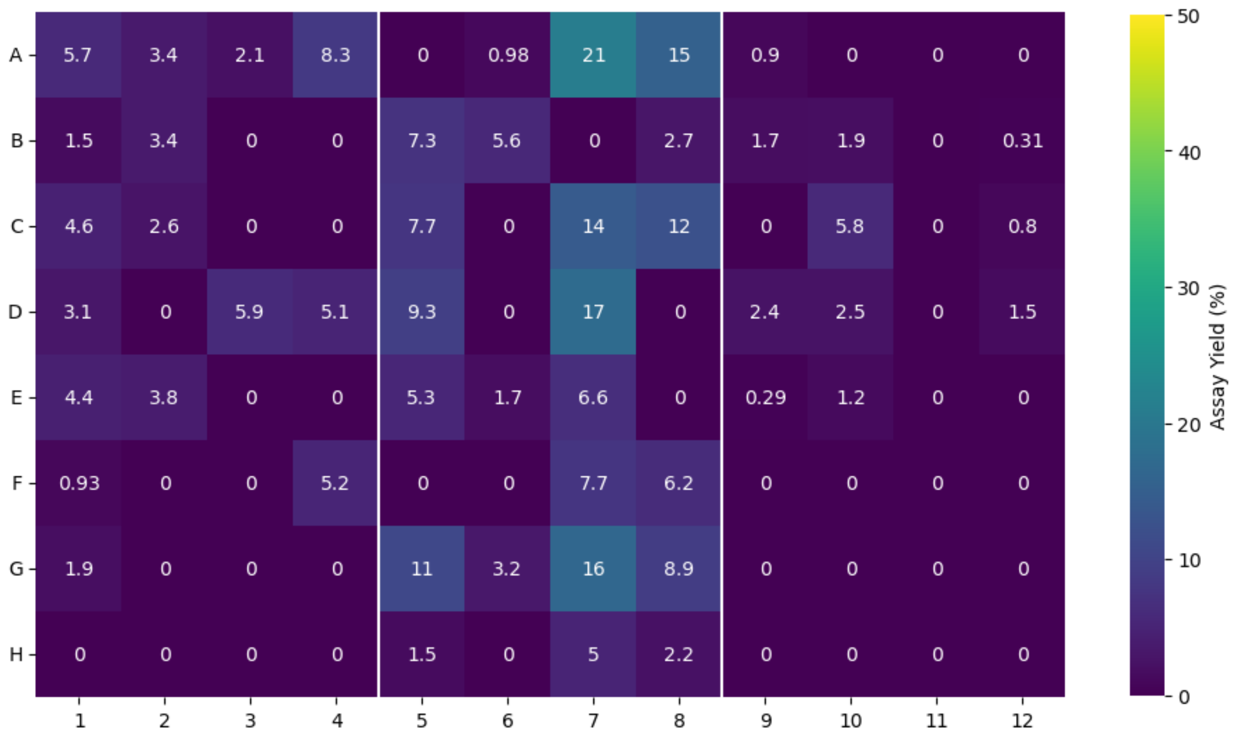

Figure S4. Assay yield heatmap of screen 2 (see Table S2).

Screen 3

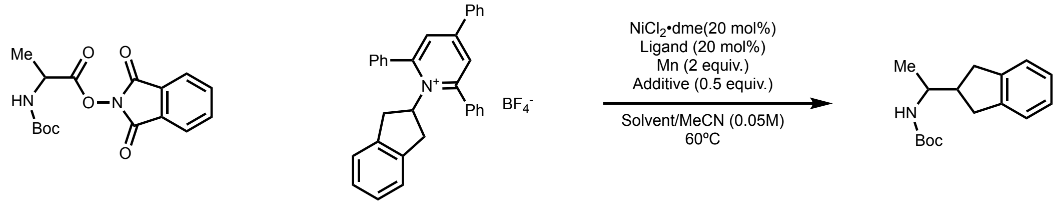

Table S3. Reagents, stock solution concentrations, dosing volumes and well locations in screen 3.

Reagents	Solvent	C_{stock} (M)	V_{dose} (μL)	Wells
BocAla-NHPI	1,4-dioxane	0.52	25	A1-D3
BocAla-NHPI	DMA	0.52	25	A4-D6
Indan pyridinium	1,4-dioxane	0.4	25	A1-D3
Indan pyridinium	DMA	0.4	25	A4-D6
$NiCl_2$•dme	1,4-dioxane	0.08	25	A1-D3
$NiCl_2$•dme	DMA	0.08	25	A4-D6
4,4'-dimethyl-2,2'-bipyridine	1,4-dioxane	0.08	25	A-D 1
4,4'-dimethyl-2,2'-bipyridine	DMA	0.08	25	A-D 4
4,4'-bis(trifluoromethyl)-2,2'-bipyridine	1,4-dioxane	0.08	25	A-D 2
4,4'-bis(trifluoromethyl)-2,2'-bipyridine	DMA	0.08	25	A-D 5
5,5'-bis(trifluoromethyl)-2,2'-bipyridine	1,4-dioxane	0.08	25	A-D 3
5,5'-bis(trifluoromethyl)-2,2'-bipyridine	DMA	0.08	25	A-D 6
TMSCl	MeCN	0.1	50	A1-A6
LiCl	MeCN	0.1	50	B1-B6
$MgCl_2$	MeCN	0.1	50	C1-C6
$ZnCl_2$	MeCN	0.1	50	D1-D6
Mn	MeCN	0.4	50	All

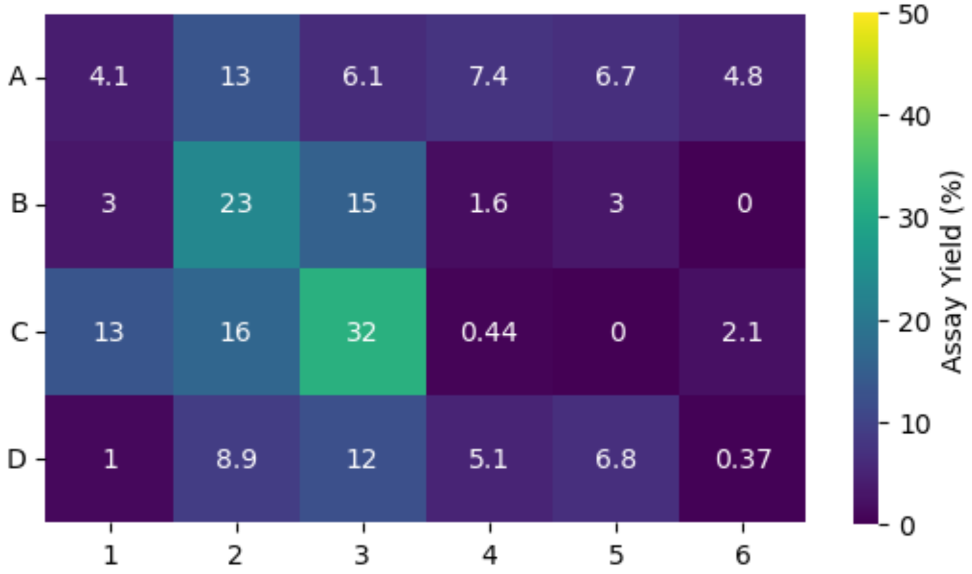

Figure S5. Assay yield heatmap of screen 3 (see Table S3).

Screen 4

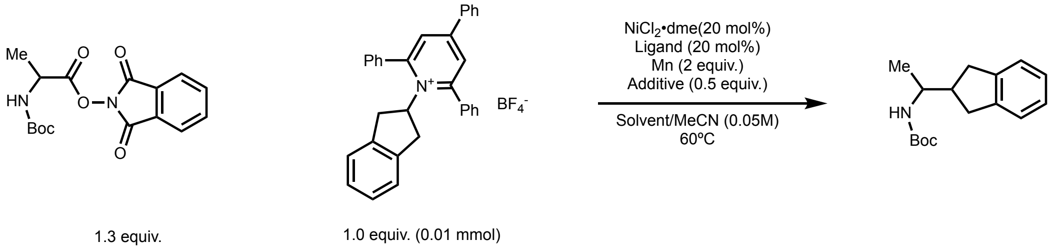

Table S4. Reagents, stock solution concentrations, dosing volumes and well locations in screen 4.

Reagents	Solvent	C_{stock} (M)	V_{dose} (μL)	Wells
BocAla-NHPI	THF	0.52	25	A1-D2
BocAla-NHPI	NMP	0.52	25	A3-D4
BocAla-NHPI	glyme	0.52	25	A5-D6
Indan pyridinium	THF	0.4	25	A1-D2
Indan pyridinium	NMP	0.4	25	A3-D4
Indan pyridinium	glyme	0.4	25	A5-D6
$NiCl_2$•dme	THF	0.08	25	A1-D2
$NiCl_2$•dme	NMP	0.08	25	A3-D4
$NiCl_2$•dme	glyme	0.08	25	A5-D6
4,4’-bis(trifluoromethyl)-2,2’-bipyridine	THF	0.08	25	A-D 1
4,4’-bis(trifluoromethyl)-2,2’-bipyridine	NMP	0.08	25	A-D 3
4,4’-bis(trifluoromethyl)-2,2’-bipyridine	glyme	0.08	25	A-D 5
4,4’-dimethyl-2,2’-bipyridine	THF	0.08	25	A-D 2
4,4’-dimethyl-2,2’-bipyridine	NMP	0.08	25	A-D 4
4,4’-dimethyl-2,2’-bipyridine	glyme	0.08	25	A-D 6
$MgCl_2$	MeCN	0.1	50	A1-A6
KBr	MeCN	0.1	50	B1-B6
NBu_4Br	MeCN	0.1	50	C1-C6
NBu_4I	MeCN	0.1	50	D1-D6
Mn	MeCN	0.4	50	All

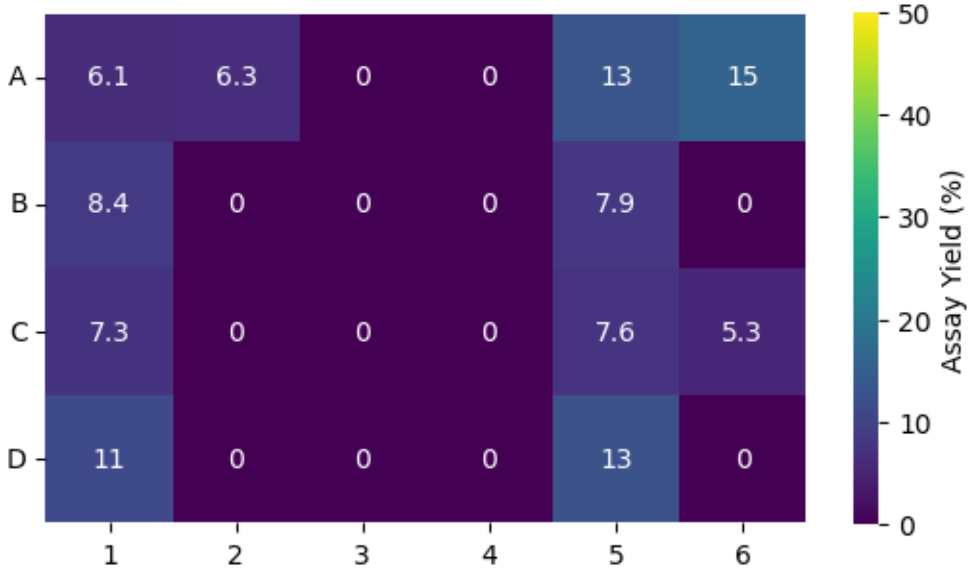

Figure S6. Assay yield heatmap of screen 4 (see Table S4).

5-2) Structure of the source dataset

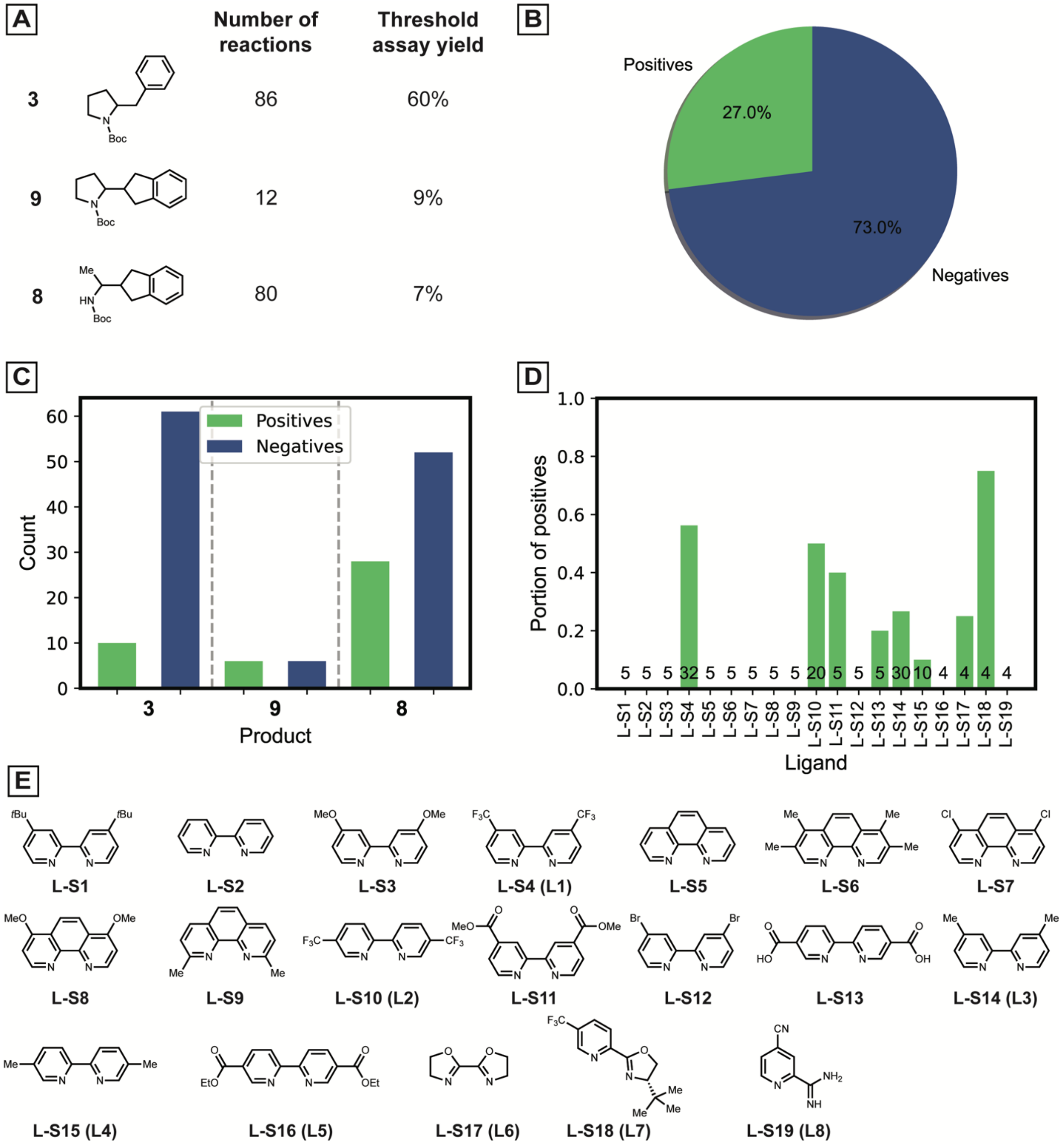

Figure S7. (A) Summary of the source dataset showing the number of reactions aiming to form each product and the threshold value used for classification. (B) Portion of positives and negatives across the entire source dataset. (C) Count of positives and negatives by product. (D) Portion of positives by ligand identity. The numbers above ligand id corresponds to the number of reactions the ligand was used in. (E) Structures of all ligands used in the source dataset.

All reaction data is provided as an excel file as well as code that processes it and generates the figures are provided on github.

5-3) Reaction featurization

The reaction studied comprises NHPI-ester, pyridinium, nickel source, ligand, additive and co-solvent to be used along with MeCN. In particular, the nickel source ($NiCl_2$•dme, $NiBr_2$•dme, NiI_2, $Ni(COD)_2$, $Ni(acac)_2$) and additive ions (anions: Cl^-, Br^-, I^-; cations: Na^+, Mg^{2+}, K^+, Zn^{2+}, TMS^+, NBu_4^+; succinimide considered separately) were represented as one-hot vectors. Descriptors were used for the remaining reaction components, as will now be delineated.

• NHPI-esters and pyridinium salts (12 descriptors): reduction potential, highest occupied molecular orbital energy (HOMO), lowest unoccupied molecular orbital energy (LUMO), dipole moment, % buried volume, sterimol L, B1 and B5 parameters around the carbon at which decarboxylation or deamination happens, spin density and % buried volume at the radical center after decarboxylation or deamination and the free energy difference in the two transformations shown below. Descriptors % buried volume and sterimol parameters were computed with the python library morfeus.[9] All other descriptors were computed at the B3LYP/6-31G* level of theory.[6]

decomposition after reduction

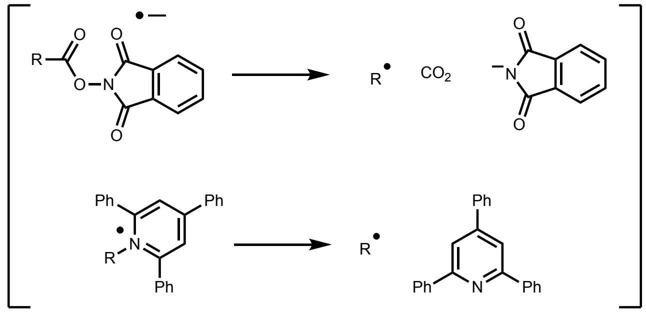

methylation of resulting radical

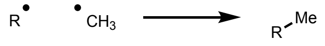

• Ligands (16 descriptors): HOMO, LUMO, dipole moment, Ni–Cl bond length, two Ni–N bond lengths, N–Ni–N bond angle, torsion of the ligand's N–C–C–N bonds, spin density at Ni, natural bond order (NBO) of Ni, NBO of N, C, C, N atoms, vibrational frequency and intensity of the Ni–Cl bond. These values were computed at B3LYP/6-31G* (LANL-2DZ for Ni) level of theory for L2–Ni(I)–Cl.[10] For example,

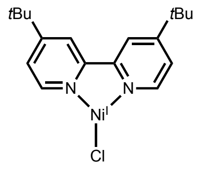

• Solvents (8 descriptors): Hansen D, P and H, Abraham Vx and Hildebrand parameters, dipole moment, dielectric constant, refractive index were extracted from the ACS solvent selection tool (https://www.acs.org/greenchemistry/research-innovation/tools-for-green-chemistry/solvent-selection-tool.html).

After representing all categorical values with descriptors, those that show extremely high correlation were pruned. In active transfer learning (ATL), the target substrate pairs, which predictions are made for, are fixed for each case study and thus their descriptors will not be used by target models to make predictions. Therefore, only the correlation between descriptors of ligands and solvents were examined and shown in Figure S8. Among ligand descriptors, LUMO (correlation of 0.95 with HOMO), spin density at Ni (correlation of 0.99 with NBO of Ni) were removed. Among solvent descriptors, dielectric constant (correlation > 0.9 with two descriptors) and Hildebrand index (correlation > 0.8 with four descriptors) were removed.

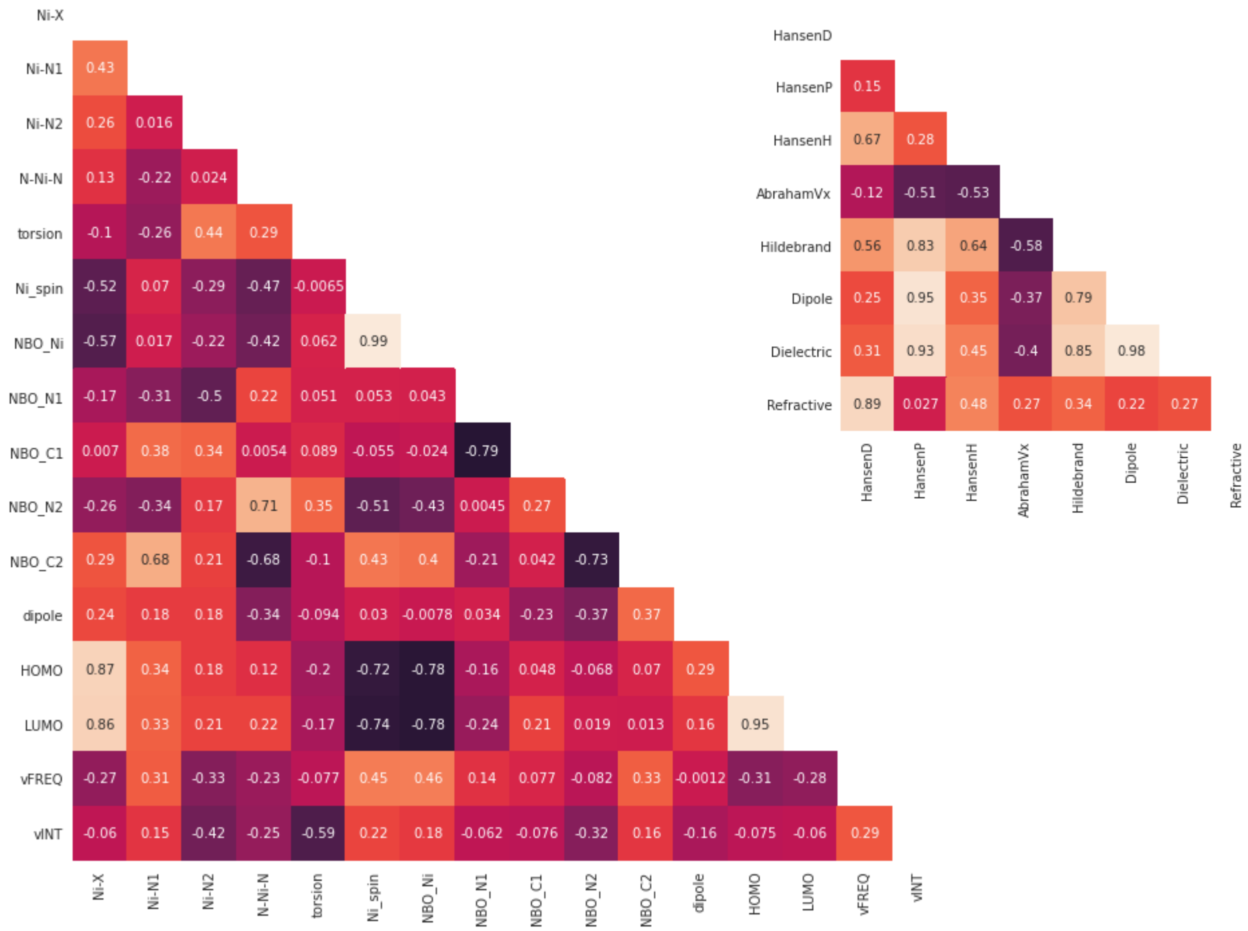

Figure S8. Pearson correlation matrix between descriptors of ligands (left) and solvents (upper right). Bright colors correspond to pairs of descriptors with high positive linear correlation.

5-4) Reaction condition candidates

To fix a set of reaction conditions to choose from with ATL, reagents that were available in our inventory in addition to all the reagents that were used during the source dataset curation were collected (see descriptors.xlsx). This resulted in five nickel sources, 29 ligands, 14 additives, and nine solvents. Enumerating all possible combinations between reagent classes resulted in a total of 18,270 reaction condition candidates.

5-5) Preliminary modeling

To get a sense of in-domain predictivity, random forest classifiers (RFC) with different combinations of hyperparameters (number of decision trees, maximum depth of each tree) were evaluated with 5-fold cross validation (CV). With only three substrate pairs, conducting leave-one-substrate-out CV is not practical. Limiting the complexity of individual decision trees had a positive effect (Figure S9).

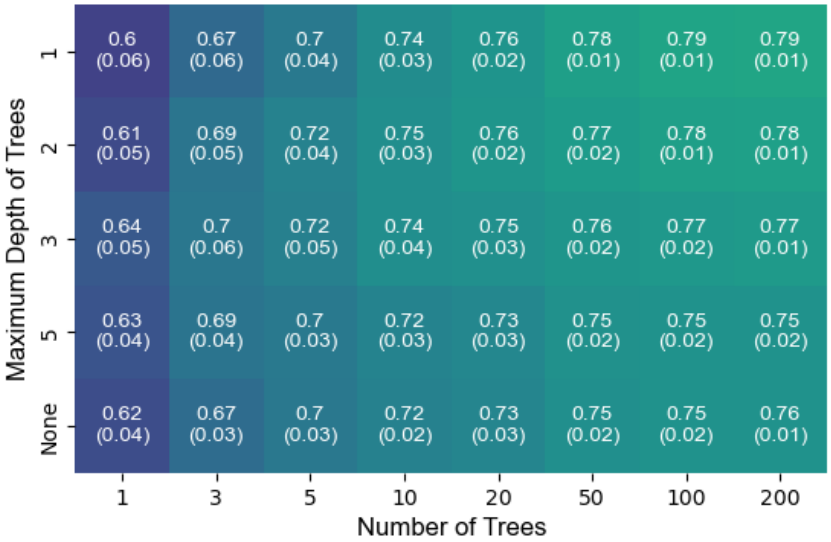

Figure S9. Heatmap of ROC-AUC scores from 5-fold CV of RFCs of different hyperparameter combinations trained with the source dataset.

In the previous retrospective study, the ATL protocol trained a single source RFC with three decision trees of depth one.[7] With a larger source dataset on a different transformation, and an aim of prospective application, the model training process was reconsidered. However, the depth of decision trees were not altered. Limiting the depth of the decision trees to one has the effect of regularizing the model, making it less overfit

to the source domain, helping the model's transferability (also see page S-46 for retrospective analysis with data generated in this study). Also, our previous study has shown the limited depth is beneficial for the source model's adaptability to the target domain as AL progresses.[7] On the other hand, we sought to reduce the variability of the source models by training an ensemble of 100 models. In the retrospective study, the number of decision trees in the source model did not impact model performance. Regardless, this point was revisited prior to beginning the prospective study.

For each value of number of decision trees in RFC ∈ {5, 10, 25, 50, 100, 200}, a set of 100 separate models were trained with different random seeds on the source dataset. Each model 'voted' for 24 reaction condition candidates with the highest predicted positive probability. These votes from the 100 models were aggregated and the distribution was shown below for the target substrate pair BocAla-NHPI (**11**) and 4-methylbenzyl pyridinium (**12**).

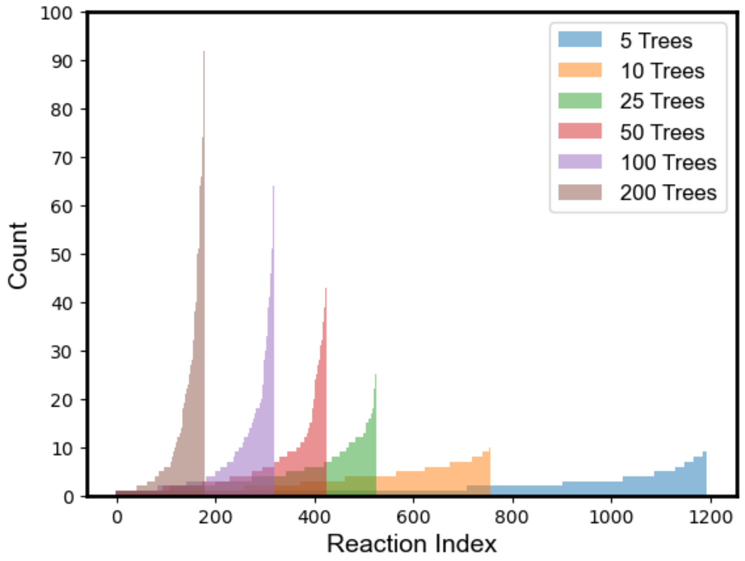

Figure S10. Distribution of reaction conditions being recommended as one of the top 24 choices from an ensemble of 100 RFCs depending on the number of decision trees. Reaction index corresponds to the total number of reaction conditions in the aggregated recordings of suggestions from RFCs in the ensemble.

As the number of decision trees increases, the diversity of reaction conditions in the top 24 decreases (compare the blue band from 5 trees with the brown band from 200 trees in Figure S10). In other words, models with a larger number of decision trees recommend a smaller, focused set of reaction conditions to conduct. From a user's

perspective, such focused suggestions are perceived to be more reliable. As the in-domain predictivity of RFCs of 100 and 200 decision trees showed minimal difference, RFCs of 100 decision trees of depth one were selected for source models.

6) Case study 1: BocAla-NHPI (11) + 4-methylbenzyl pyridinium (12)

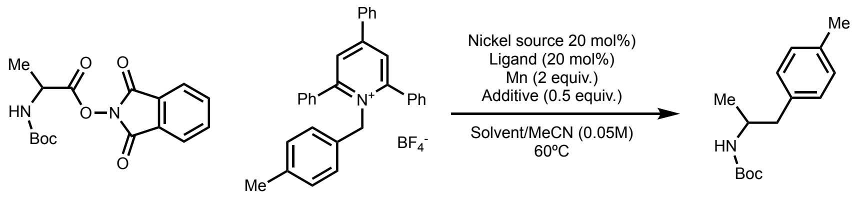

11 1.3 equiv. **12** 1.0 equiv. (0.05 mmol) **7**

The nickel-catalyzed reductive cross coupling of activated amines and carboxylic acids was discovered and developed upon benzyl pyridinium (**2**). In the previous report, the coupling of **11** and **2** yielded 65% product.[8] As such, substituting **2** for **12** was considered to be relatively straightforward and thus an appropriate initial case study.

Due to its resemblance to the representative reaction, a small number of experiments for each iteration (four) was deemed sufficient. The source model (see previous section) was used to select the first set of three reactions to carry out (in addition to the previous reaction condition as control) for coupling **11** with **12**. Under this setting, the number of votes from each RFC in the ensemble impacts the reactions recommended. Therefore, we compared 12 and 4 (smaller numbers than 24, which was used for Figure S10, were chosen due to the choice of running four reactions at a time). The table below shows the reactions which were recommended.

Table S5. Top five reactions recommended by the same ensemble of 100 RFCs but with different number of votes from each RFC. The same colors across the two tables correspond to the same reaction condition showing up in different ranks.

Vote **12** most likely reactions					Vote **4** most likely reactions				
Ni	Ligand	Additive	Solv.	Votes	Ni	Ligand	Additive	Solv.	Votes
$NiBr_2$•dme	**L1**	$MgCl_2$	tBuOMe	50	$NiBr_2$•dme	**L1**	$MgCl_2$	tBuOMe	29
$NiBr_2$•dme	**L1**	$MgCl_2$	Glyme	45	$NiBr_2$•dme	**L1**	$MgCl_2$	Glyme	27
$NiBr_2$•dme	**L1**	$MgBr_2$	Glyme	42	$NiBr_2$•dme	**L1**	$MgBr_2$	tBuOMe	17
$NiBr_2$•dme	**L1**	$MgBr_2$	tBuOMe	42	$NiCl_2$•dme	**L1**	$MgCl_2$	Glyme	16
$NiCl_2$•dme	**L1**	$MgCl_2$	Glyme	36	$NiBr_2$•dme	**L1**	$MgBr_2$	Glyme	15

As shown in Table S5, the first two suggestions were identical for the two voting schemes. Furthermore, although the orders are different, the set of 3rd~5th suggestions are the same, implying the robustness of this ensemble approach.

In terms of specific reagents, the consistent recommendation of **L1** stands out. This is because the vast majority of the descriptors employed in the RFCs are those of ligands (see Figure S15). Based on the models' perception of ligand importance, ATL's greedy approach strongly prioritizes the use of **L1** for target compounds. The selection of two additives and solvents, respectively, probably stems from their success in promoting the coupling of **11** and indan-2-yl pyridinium **10** (forming **8**) which is part of the source dataset.

After confirming the stability of the predictions across different voting schemes, the study proceeded with each RFC voting 12 reaction conditions. In the first iteration, three reactions from the top left of Table S5 along with a control using the previously reported reaction condition were conducted. The reactions were conducted at 0.05 mmol scale (see General Procedure D) using 4 mL vials as reaction vessels. The results (also shown in Figure 4C), show no improved yields.

Table S6. Results of 0.05 mmol scale experiments in the first ATL iteration. [a]0.15 mmol scale.

Description	Ni	Ligand	Additive	Solv.	Votes	Assay Yield (%)
Control[a]	$NiBr_2$•dme	**L1**	none	dioxane	n/a	55
Batch 1	$NiBr_2$•dme	**L1**	$MgCl_2$	tBuOMe	50	34
	$NiBr_2$•dme	**L1**	$MgCl_2$	Glyme	45	14
	$NiBr_2$•dme	**L1**	$MgBr_2$	Glyme	42	17

With no entries returning yield above that from the control (55%), a meaningful target RFC model cannot be trained as a negative prediction will be made regardless of the input. As an alternative, the decision trees within the source RFCs that predicted any of these reactions to be positive were ignored, by setting their weights to 0. This resulted in an average of 38.7 trees being removed from the RFCs that began with 100 trees in the ensemble. With this smaller ensemble of RFCs, the batch of reactions to conduct in the second iteration was recommended. The top three conditions shared the nickel

source, ligand, and solvent. Due to the high chemical similarity of **12** to **2** (which performed well without using additives), the fourth reaction was selected with the same reaction components without the additive. Same as above, the reactions were conducted at 0.05 mmol scale using 4 mL vials.

Table S7. Results of 0.05 mmol scale experiments in the second ATL iteration.

Description	Ni	Ligand	Additive	Solv.	Votes	Assay Yield (%)
Batch 2	$NiCl_2$•dme	**L1**	none	THF	n/a	65
	$NiCl_2$•dme	**L1**	KCl	THF	24	23
	$NiCl_2$•dme	**L1**	$MgBr_2$	THF	18	45
	$NiCl_2$•dme	**L1**	$MgCl_2$	THF	18	44

By changing the nickel source to $NiCl_2$•dme and solvent to THF and not using any additive, the yield of **7** improved by 10% compared to the previous condition. The key of not employing an additive was not suggested by the model, but rather by chemical intuition. This suggests the role that chemists can play in guiding autonomous exploration.

Considering this as a success for an initial target, further optimization of continuous variables at a larger scale of 0.15 mmol scale was pursued subsequently, as described in Table S8. By increasing the equivalents of Mn from 2 to 3, an average of 63% of **7** was isolated (entry 3, 64%, 62%).

Table S8. Results of 0.15 mmol scale experiments screening different continuous variables. [a]Values in parentheses are isolated yields. [b]Average of two runs.

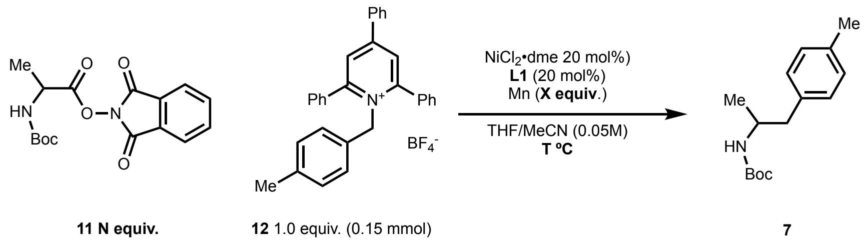

Entry	Equiv. of 11	Mn equiv.	Temperature (ºC)	Assay yield (%)[a]
**1**	1.3	2	60	65 (60)
**2**	2	2	60	41
**3**	1.3	3	60	(63)[b]
**4**	1.3	2	50	68 (59)
**5**	1.3	3	50	59 (63)

7) Case study 2: BocPro-NHPI (1) + mexiletine-derived pyridinium (13)

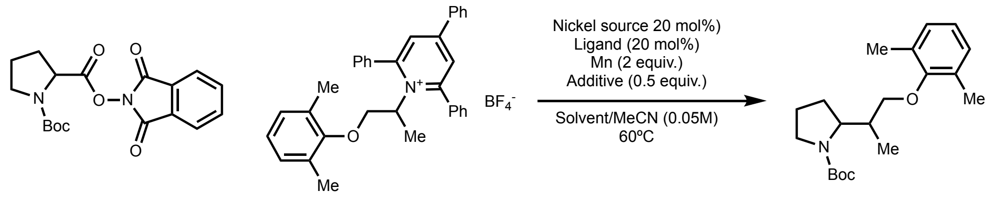

1 1.3 equiv. **13** 1.0 equiv. (0.01 mmol) **14**

In addition to being a secondary acyclic alkyl pyridinium, the presence of both an oxygen alpha to the radical center resulting from deamination and a bulky 2,6-dimethyl phenyl group in **13** makes it a difficult coupling partner. Indeed, two reactions run at 0.05 mmol scale and 0.15 mmol scale show 7% assay and 12% isolated yields, respectively. 7% was used as the threshold for binary classification.

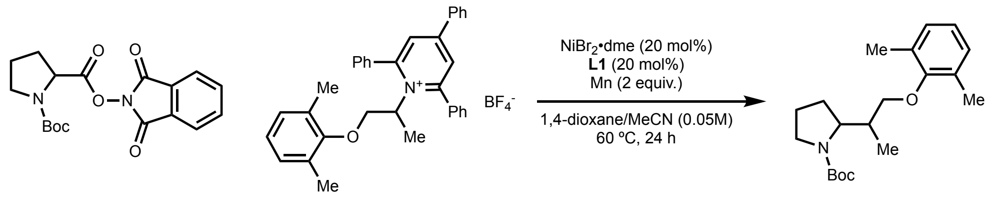

1 1.3 equiv. **13** 1.0 equiv. 0.05 mmol 0.15 mmol **14** 7% assay yield 12% isolated yield

To enhance our chance of identifying an improved reaction condition, 24-well high throughput experimentation at 0.01 mmol scale was used. Using the same source model used in case study 1, each voting for 24 reactions, the first set of 24 reaction condition was collected as Table S9 below. For experimental convenience, the 22nd and 24th suggestions were substituted with [Ni(COD)$_2$, **L1**, $MgCl_2$, Bu_2O] and [Ni(COD)$_2$, **L1**, $MgBr_2$, Bu_2O], resulting in a grid of [4 Ni sources] × [2 additives] × [3 solvents]. The HTE recipe and assay yield results are shown in the next page.

Table S9. List of 24 suggestions from the source model.

Entry	Ni source	Ligand	Additive	Solvent	Vote count
1	$NiBr_2$•dme	**L1**	$MgCl_2$	Glyme	69
2	$NiBr_2$•dme	**L1**	$MgCl_2$	tBuOMe	68
3	$NiBr_2$•dme	**L1**	$MgBr_2$	Glyme	64
4	$NiBr_2$•dme	**L1**	$MgBr_2$	tBuOMe	63
5	$NiCl_2$•dme	**L1**	$MgCl_2$	Glyme	51
6	$NiCl_2$•dme	**L1**	$MgCl_2$	tBuOMe	49
7	$NiBr_2$•dme	**L1**	$MgCl_2$	Bu_2O	46
8	$NiCl_2$•dme	**L1**	$MgBr_2$	tBuOMe	46
9	$NiCl_2$•dme	**L1**	$MgBr_2$	Glyme	45
10	NiI_2	**L1**	$MgCl_2$	tBuOMe	41
11	$NiBr_2$•dme	**L1**	$MgBr_2$	Bu_2O	41
12	NiI_2	**L1**	$MgCl_2$	Glyme	40
13	$NiCl_2$•dme	**L1**	$MgCl_2$	Bu_2O	39
14	NiI_2	**L1**	$MgBr_2$	tBuOMe	36
15	NiI_2	**L1**	$MgBr_2$	Glyme	36
16	$NiCl_2$•dme	**L1**	$MgBr_2$	Bu_2O	33
17	$Ni(COD)_2$	**L1**	$MgCl_2$	Glyme	31
18	$Ni(COD)_2$	**L1**	$MgCl_2$	tBuOMe	30
19	$Ni(COD)_2$	**L1**	$MgBr_2$	tBuOMe	30
20	NiI_2	**L1**	$MgCl_2$	Bu_2O	28
21	$Ni(COD)_2$	**L1**	$MgBr_2$	Glyme	28
22	$NiBr_2$•dme	**L1**	$MgCl_2$	MeCN	25
23	NiI_2	**L1**	$MgBr_2$	Bu_2O	24
24	$NiBr_2$•dme	**L1**	KCl	tBuOMe	23

*Table S10. Reagents, stock solution concentrations, dosing volumes and well locations in the first iteration of ATL to form **9**.*

Reagents	Solvent	C_{stock} (M)	V_{dose} (µL)	Wells
BocPro-NHPI	glyme	0.52	25	A1-D2
BocPro-NHPI	Bu_2O	0.52	25	A3-D4
BocPro-NHPI	tBuOMe	0.52	25	A5-D6
Mexiletine pyridinium	glyme	0.4	25	A1-D2
Mexiletine pyridinium	Bu_2O	0.4	25	A3-D4
Mexiletine pyridinium	tBuOMe	0.4	25	A5-D6
$NiCl_2$•dme	glyme	0.08	25	A1-A2
$NiCl_2$•dme	Bu_2O	0.08	25	A3-A4
$NiCl_2$•dme	tBuOMe	0.08	25	A5-A6
$NiBr_2$•dme	glyme	0.08	25	B1-B2
$NiBr_2$•dme	Bu_2O	0.08	25	B3-B4
$NiBr_2$•dme	tBuOMe	0.08	25	B5-B6
NiI_2	glyme	0.08	25	C1-C2
NiI_2	Bu_2O	0.08	25	C3-C4
NiI_2	tBuOMe	0.08	25	C5-C6
$Ni(COD)_2$	glyme	0.08	25	D1-D2
$Ni(COD)_2$	Bu_2O	0.08	25	D3-D4
$Ni(COD)_2$	tBuOMe	0.08	25	D5-D6
4,4'-bis(trifluoromethyl)-2,2'-bipyridine	tBuOMe	0.08	25	A1-D2
4,4'-bis(trifluoromethyl)-2,2'-bipyridine	Bu_2O	0.08	25	A3-D4
4,4'-bis(trifluoromethyl)-2,2'-bipyridine	glyme	0.08	25	A5-D6
$MgCl_2$	MeCN	0.1	50	A-D 1, 3, 5
$MgBr_2$	MeCN	0.1	50	A-D 2, 4, 6
Mn	MeCN	0.4	50	All

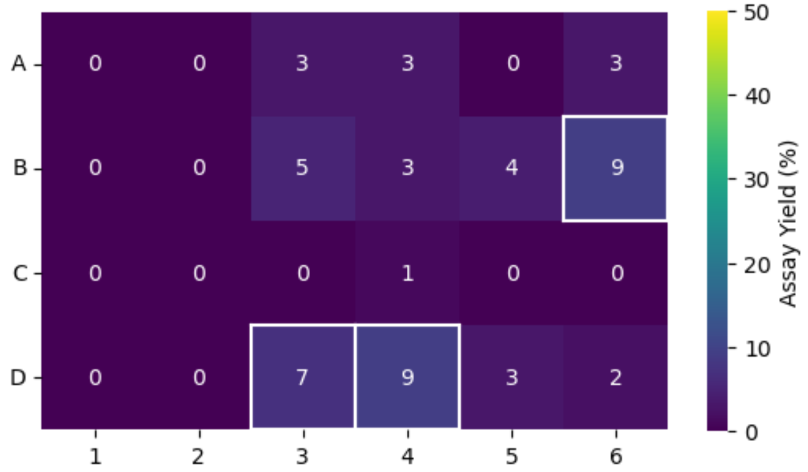

*Figure S11. Assay yield heatmap of first iteration of ATL for **9**. Positives are marked with white squares.*

Among the first set of 24 target reactions, three positives that give equal to or greater than the threshold were observed. Two of them, wells D3 and D4, corresponds to reactions with $Ni(COD)_2$ with $MgCl_2$ or $MgBr_2$ in Bu_2O. The other, well B6, is the reaction with $NiBr_2$•dme, $MgBr_2$ in tBuOMe. However, as assay yields below 10% point to the need for further improvement, the exploration continued.

To continue the ATL campaign, target RFCs – RFC trained on the newly obtained set of 24 target reactions – needed to be trained. To match the number of models in the source ensemble, 100 target RFCs were trained. The number of trees was heuristically selected. In our previous report, each target model had three trees trained on three target reactions. As the number of target reactions to train on and number of trees in the source models are larger than the previous study, increasing the number of target trees was considered appropriate. However, as some reagents are commonly used across multiple conditions, keeping the number smaller than the number of sampled target reactions seemed reasonable. As such, the number of target trees was set to 15.

With the number of trees in the target model fixed, how the number of trees in the source RFC impacts recommendations was inspected next. The target RFC ensemble was combined with two source RFC ensembles each with 25 and 100 decision trees, respectively. In order to combine an ensemble of 100 target RFCs with an ensemble of 100 source RFCs, a target RFC was randomly assigned to a source RFC and their trees were combined, for all 100. The distribution of recommendations is shown below. Interestingly, the combined RFC with a smaller number of decision trees gave a narrower set of recommendations, unlike in Figure S10. Although the reason beneath this observation is unclear, as the agreement between the models are higher, for this case study, we decided to proceed with the RFC with the smaller number of trees.

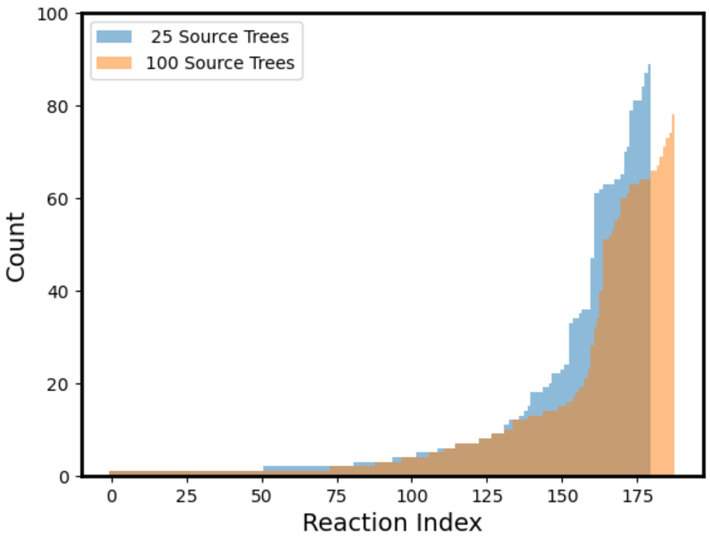

Figure S12. Distribution of reaction conditions being recommended as one of the top 24 choices for RFCs of different number of decision trees. Narrower blue band and higher bars mean higher agreement of suggested reaction conditions across the 100 RFCs.

The HTE recipe shown in Table S11 lists the conditions that were recommended to survey. The number of additives that are screened particularly stands out. In fact, the model queries all remaining additives not used in the first iteration. However, during preliminary experiments after the source data collection, reactions with $ZnCl_2$ produced *N*-Boc pyrrolidine when **1** was a substrate (which has not been observed in any other reactions), probably due to acceleration of decarboxylation outpacing any other process. Therefore, $ZnCl_2$ was manually removed from the list of additives, along with TMSCl (for experimental convenience). The remaining 10 additives were subject to screening with two nickel sources ($NiBr_2$•dme and $Ni(COD)_2$) that provided the positives in the first round, leaving four vacant wells. This was occupied by the other nickel source, $Ni(acac)_2$, which was not studied in the first iteration, with four different additives. The first half of the solvent is fixed to Bu_2O, also due to its success in the first iteration, reflecting the greedy approach of the ATL protocol.

As shown in Figure S13, among the 24 reactions screened, no reactions using $Ni(acac)_2$ gave positive results (column 6). Additive KBr returned positives with both $NiBr_2$•dme and $Ni(COD)_2$ (cells A4 and C4, respectively), while $Ni(COD)_2$ with TBABr gave a comparable result (cell D4).

*Table S11. Reagents, stock solution concentrations, dosing volumes and well locations in the second iteration of ATL to form **9**.*

Reagents	Solvent	C_{stock} (M)	V_{dose} (μL)	Wells
BocPro-NHPI	Bu_2O	0.52	25	All
Mexiletine pyridinium	Bu_2O	0.4	25	All
$NiBr_2$•dme	Bu_2O	0.08	25	A1-B5
$Ni(COD)_2$	Bu_2O	0.08	25	C1-D5
$Ni(acac)_2$	Bu_2O	0.08	25	A6-D6
4,4'-bis(trifluoromethyl)-2,2'-bipyridine	Bu_2O	0.08	25	All
No additive	MeCN	n/a	50	A1, C1
Succinimide	MeCN	0.1	50	A2, C2
KCl	MeCN	0.1	50	A3, C3
KBr	MeCN	0.1	50	A4, C4, B6
KI	MeCN	0.1	50	A5, C5
NaCl	MeCN	0.1	50	B1, D1
NaI	MeCN	0.1	50	B2, D2
TBACl	MeCN	0.1	50	B3, D3
TBABr	MeCN	0.1	50	B4, D4, C6
TBAI	MeCN	0.1	50	B5, D5, D6
$MgBr_2$	MeCN	0.1	50	A6
Mn	MeCN	0.4	50	All

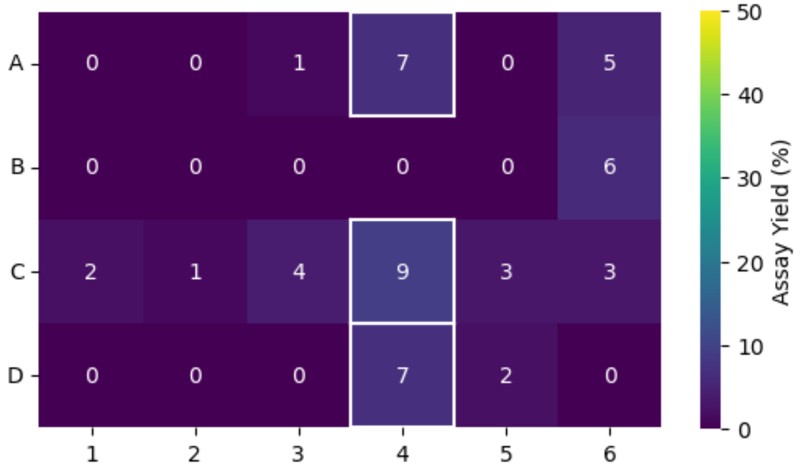

Figure S13. Assay yield heatmap of second iteration of ATL for 9. Positives are marked with white squares.

The second set of 100 target RFCs of 15 decision trees of depth one was trained on the data collected in Figure S14. Each RFC was then combined with a previous model to update the previous set of models. The resulting model was used to collect the next

set of conditions to conduct. A few experiments recommended included NiI_2 as the nickel source, but due to experimental convenience, we decided to focus more on other nickel sources. The resulting recipe for HTE is shown in Table S12 and its results in Figure S15.

*Table S12. Reagents, stock solution concentrations, dosing volumes and well locations in the third iteration of ATL to form **9**.*

Reagents	Solvent	C_{stock} (M)	V_{dose} (μL)	Wells
BocPro-NHPI	Dioxane	0.52	25	A1-D1
BocPro-NHPI	CPME	0.52	25	A2-D2
BocPro-NHPI	tBuOMe	0.52	25	A3-D3
BocPro-NHPI	Bu_2O	0.52	25	A4-D6
Mexiletine pyridinium	Dioxane	0.4	25	A1-D1
Mexiletine pyridinium	CPME	0.4	25	A2-D2
Mexiletine pyridinium	tBuOMe	0.4	25	A3-D3
Mexiletine pyridinium	Bu_2O	0.4	25	A4-D6
$NiCl_2$•dme	Dioxane	0.08	25	A1
$NiCl_2$•dme	CPME	0.08	25	A2
$NiCl_2$•dme	tBuOMe	0.08	25	A3
$NiCl_2$•dme	Bu_2O	0.08	25	A4-D4
$NiBr_2$•dme	Dioxane	0.08	25	B1
$NiBr_2$•dme	CPME	0.08	25	B2
$NiBr_2$•dme	tBuOMe	0.08	25	B3
$NiBr_2$•dme	Bu_2O	0.08	25	A5-A6
$Ni(COD)_2$	Dioxane	0.08	25	C1
$Ni(COD)_2$	CPME	0.08	25	C2
$Ni(COD)_2$	tBuOMe	0.08	25	C3
$Ni(COD)_2$	Bu_2O	0.08	25	B5-D6
$Ni(acac)_2$	Dioxane	0.08	25	D1
$Ni(acac)_2$	CPME	0.08	25	D2
$Ni(acac)_2$	tBuOMe	0.08	25	D3
4,4’-bis(trifluoromethyl)-2,2’-bipyridine	Dioxane	0.08	25	A1-D1
	CPME	0.08	25	A2-D2
	tBuOMe	0.08	25	A3-D3
	Bu_2O	0.08	25	A4-D6
KBr	MeCN	0.1	50	A1-D3, B4, A5-D6
KCl	MeCN	0.1	50	A4
KI	MeCN	0.1	50	C4
TBABr	MeCN	0.1	50	D4-D6
$MgBr_2$	MeCN	0.1	50	C5-C6
Mn	MeCN	0.4	50	All

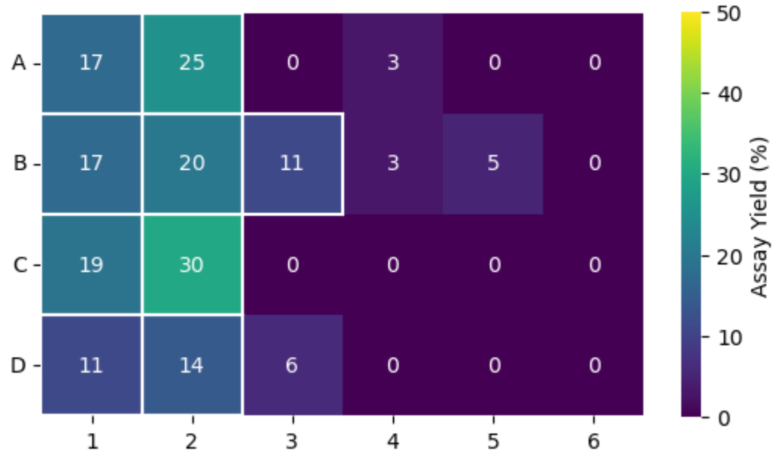

Figure S14. Assay yield heatmap of third iteration of ATL for 9. Positives are marked with white squares.

Using either dioxane (column 1) or CPME (column 2), both the number of positives (9 vs, 2) and observed assay yield was significantly enhanced compared to the previous two batches. This behavior of making better, higher-yielding recommendations at the third iteration is consistent with our previous report. To support the hypothesis that models make better suggestions once data spanning all components of the reaction condition is available, the models were further analyzed. Specifically, which reaction component's features were utilized by the model at each iteration was inspected.

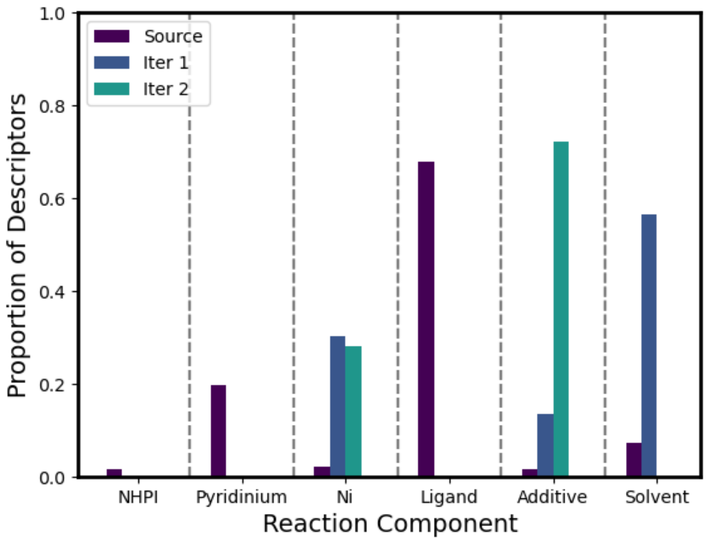

Figure S15. Descriptors used by source and target RFCs at different iterations.

The purple bars in Figure S16 show how source RFCs made use of the descriptors available in the dataset. Ligand descriptors are dominant, emphasizing the importance of the ligand in this reaction. From the other perspective, the source model considers the ligand to be the most important, which explains why the ligand was fixed to **L1** in all of its recommendations.

Due to the focus of source models on ligands, it has not learned enough about the impact of other components on the reaction outcome. The blue bars, which indicate the descriptors learned by the first target RFCs, show that this has been supplemented mostly by learning about the solvent and nickel source. This behavior is continued, as seen from the green bars showing up at components where the heights of the purple and blue bars were relatively short (additives and nickel sources). The combined model – comprising the source, iteration 1 and 2 target RFCs – can make predictions based on all of the condition components, which in return provided significantly improved results as in Figure S15.

With notably improved results obtained after three iterations of 24-well screens, subsequent studies were conducted at a larger scale of 0.15 mmol (Table S13). Initially, the two best reactions, run in A2 and C2, were conducted, resulting in isolated yields within 10% deviation from assay yields observed in Figure S15. However, these results were shown to be difficult to be reproduce, possibly due to the reaction being sensitive to the quality of CPME used. As such, reactions in dioxane, notably the well B1 was subject to scale-up which consistently returned 20% yield in two separate runs.

Table S13. Initial scale up experiments of best performing reactions from Figure S15. Shown are isolated yields.

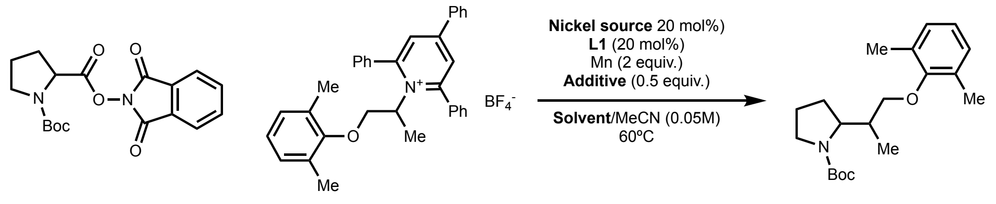

1 1.3 equiv. **13** 1.0 equiv. (0.15 mmol) **14**

Entry	Ni Source	Additive	Solvent	Yield (%)	Comment
Control	$NiBr_2$•dme	None	dioxane	12	
C2	$Ni(COD)_2$	KBr	CPME	22	irreproducible
A2	$NiCl_2$•dme	KBr	CPME	32	
B1	$NiBr_2$•dme	KBr	dioxane	20	Average of two runs

With a reliable, improved (compared to the previous report) reaction condition in hand, subsequent stoichiometry studies were conducted at 0.15 mmol scale (Table S14). Decreasing the temperature resulted in modestly increased yields (20 to 25%, entry 3). Increasing the equivalents of Mn further improved the yield to 33% (entry 4). Other modifications, such as even more Mn (entry 5), even lower temperature (entry 6), altering the equivalents of KBr (entries 7, 8) or stoichiometry between reactants (entries 9-11), did not improve yield. However, combining multiple modifications simultaneously further enhanced the yield to 43% (entry 13).

*Table S14. Further optimization of continuous variables at 0.15 mmol scale. Bold values correspond to those different from the previous report. Isolated yields are shown. [a]) 10 mol% NiBr2•dme and **L1** was used instead of 20 mol%. [b]) Average of two runs (44%, 41%).*

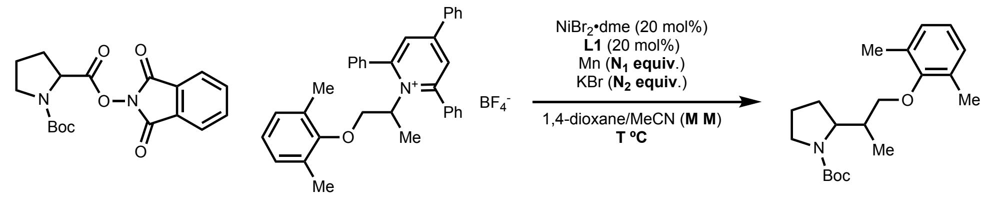

1 **13** **14**

Entry	Limiting Reagent	other reagent equiv.	N_1	N_2	M	T	Yield (%)
1	**13**	1.3	2	0	0.05	60	12
2	**13**	1.3	2	0.5	0.05	60	20
3	**13**	1.3	2	0.5	0.05	**50**	25
4	**13**	1.3	**3**	0.5	0.05	**50**	33
5	**13**	1.3	**4**	0.5	0.05	**50**	32
6	**13**	1.3	**3**	0.5	0.05	**40**	34
7	**13**	1.3	**3**	**0.25**	0.05	**50**	33
8	**13**	1.3	**3**	**1.0**	0.05	**50**	25
9	**13**	**2.0**	**3**	0.5	0.05	**50**	32
10	**1**	1.3	**3**	0.5	0.05	**50**	30
11	**13**	1.3	**3**	0.5	**0.0375**	**50**	32
12	**13**	**2.0**	**3**	**0.25**	0.05	**40**	39
13[a]	**13**	**2.0**	**3**	**0.25**	0.05	**40**	28
14[a]	**13**	**2.0**	**3**	**0.25**	**0.0375**	**40**	**43[b]**
15[a]	**13**	**2.0**	**3**	0	**0.0375**	**40**	36

The results up to now show that improved reaction conditions for single substrate pairs can be improved with ATL, but it is not yet clear that the improvement would be transferrable to related substrates. Accordingly, transferability was evaluated on two pyridinium salts that are similar to **13**. While one was chemically simpler without the oxygen and steric-inducing methyl groups (forming **S1**), the other was thought to be similarly challenging, retaining both the oxygen and bulk (forming **S2**). The reaction condition further optimized in Table S14 (entry 4) was compared to the previous reaction

condition on forming **S1** and **S2**. The latter returned yields of 31% for **S1** and 23% for **S2**, while the reaction condition identified by ATL and further optimized gave improved values of 39% and 28%, respectively. While these are modest improvements, they suggest the applicability of an ATL-identified conditions to chemically related substrates.

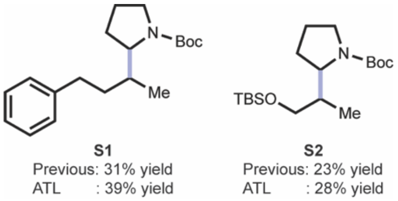

*Scheme S1. Reaction condition identified by ATL and further optimized shows modest increase in yield for coupling **1** and chemically relevant pyridinium substrates, compared to the previously reported condition.*

8) Case study 3: BocPro-NHPI (1) + Indan-2-yl pyridinium (10)

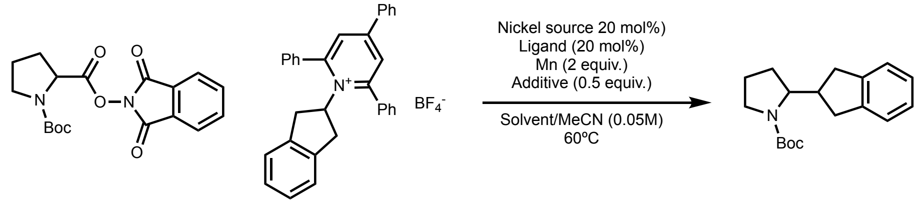

1 1.3 equiv. **10** 1.0 equiv. (0.05 mmol) **9**

45% isolated yield of **9** was achieved in the course of curating the source dataset which is an improvement from the prior condition's 37%. With 12 reactions included in the source dataset, ATL was tested if further improvements could be made in a short time period. As this case study shares **1** with case study 2, the data collected from the previous campaign was included as a part of the source dataset. An ensemble of 100 RFCs with 100 trees of depth one was trained as the initial source model. The dozen reactions coupling **1** and **10** conducted for curating the source dataset was used to train the initial target RFC ensemble where each RFC has 15 trees of depth one.

In order to get a single set of reaction condition recommendations, the source model was updated by combining with the initial target RFC ensemble (resulting in a total 115 trees). From this combined model, the list of recommendations from different number of votes from each model are compared again in the table below. As we considered to run six reactions in parallel, we initially considered vote numbers of 24 and 6.

Table S15. Top seven reactions recommended as an ensemble of 100 RFCs voting for different numbers of highest probable reactions. The same colors across the two tables correspond to the same reaction condition showing up in a different rank.

Vote 24 most likely reactions					Vote 6 most likely reactions				
Ni	Ligand	Additive	Solv.	Votes	Ni	Ligand	Additive	Solv.	Votes
$NiBr_2$•dme	**L1**	KBr	THF	99	$NiBr_2$•dme	**L1**	KBr	THF	92
$NiBr_2$•dme	**L1**	KBr	Dioxane	96	$NiBr_2$•dme	**L1**	KBr	Dioxane	69
$NiBr_2$•dme	**L1**	KCl	THF	89	$NiBr_2$•dme	**L1**	KCl	THF	51
$NiBr_2$•dme	**L1**	KBr	CPME	86	$NiBr_2$•dme	**L1**	KI	THF	46
$NiBr_2$•dme	**L1**	KI	THF	84	$NiBr_2$•dme	**L1**	KBr	MeCN	39
$NiBr_2$•dme	**L1**	KBr	MeCN	81	$NiBr_2$•dme	**L1**	KBr	CPME	31
$NiBr_2$•dme	**L1**	KCl	Dioxane	76	$NiBr_2$•dme	**L1**	KCl	Dioxane	28

Similar observations are made to those in case study 1. Up to the third entry, the recommended reaction conditions are identical, and the set of next three comes in a different order, confirming the robustness of using an ensemble. On the other hand, the additives are all K^+, which is likely due to their success in improving the yield of coupling **1** and **13**.

The top six recommendations were conducted on a 0.05 mmol scale in a 4 mL vial (see General Procedure D). The results are shown below.

Table S16. Results of 0.05 mmol scale experiments in the first ATL iteration.

**Description**	**Ni**	**Ligand**	**Additive**	**Solv.**	**Assay Yield (%)**
Batch 1	$NiBr_2$•dme	**L1**	KBr	THF	32
	$NiBr_2$•dme	**L1**	KBr	Dioxane	25
	$NiBr_2$•dme	**L1**	KCl	THF	33
	$NiBr_2$•dme	**L1**	KI	THF	33
	$NiBr_2$•dme	**L1**	KBr	CPME	33
	$NiBr_2$•dme	**L1**	KBr	MeCN	24

By setting 32% assay yield and above as ‘positives’, a set of 100 target RFCs, each with 15 decision trees, was trained and added to the previous model with 115 decision trees. Similar to the last iteration, six reactions were chosen by aggregating 24 votes from each model. $ZnCl_2$ was one of the recommended additives. However, in

preliminary studies, $ZnCl_2$ was shown to form *N*-Boc pyrrolidine (decarboxylated **1**) which has not been observed with any other additives or reaction conditions. Assuming this is due to accelerated decarboxylation that does not match in rate with deamination processes, the recommendation was not conducted. The subsequent top six recommendations were conducted at a 0.05 mmol scale in a 4 mL vial and their results are shown below in Table S17.

Similar to the second iteration in case study 2 (Figure S14), the recommendations only varied in the additives. More specifically, Cl^- and I^- salts with cations other than K^+ were suggested. This is because both KCl and KI gave positives while KBr gave mixed results in the first batch.

Table S17. Results of 0.05 mmol scale experiments in the second ATL iteration.

Description	Ni	Ligand	Additive	Solv.	Assay Yield (%)
Batch 2	$NiBr_2$•dme	**L1**	TBACl	THF	20
	$NiBr_2$•dme	**L1**	TBAI	THF	24
	$NiBr_2$•dme	**L1**	NaCl	THF	31
	$NiBr_2$•dme	**L1**	NaI	THF	28
	$NiBr_2$•dme	**L1**	$MgCl_2$	THF	31
	$NiBr_2$•dme	**L1**	TMSCl	THF	33

Major improvement in outcome was not observed by this second iteration. When the positive assay yield threshold of 32% is applied, only entry 6 is positive. This was problematic for training RFCs because of the bootstrap procedure for training decision trees. That is, to train a decision tree, the six-reaction data are not used as is, but they are sampled six times with replacements (i.e., one entry can be selected multiple times). As a result, if the single positive entry is not selected, a decision tree cannot be trained because all of the training data have negative labels. To address this technical problem, the single positive entry was duplicated. Following the ATL protocol, 100 RFCs, each with 15 trees of depth 1 was trained and combined to update the model from the previous iteration. Recommendation of the next set of experiments were collected by letting each RFC to vote 24 most probably positive reactions. Entries using NiI_2 as the nickel source were ignored due to its consistent failure in previous experiments. TMSCl was

consistently suggested throughout the top-6 entries probably due to it being the only success in Table S17.

Table S18. Results of 0.05 mmol scale experiments in the third ATL iteration.

Description	Ni	Ligand	Additive	Solv.	Assay Yield (%)
Batch 3	$NiCl_2$•dme	**L1**	TMSCl	CPME	48
	$NiCl_2$•dme	**L1**	TMSCl	THF	39
	$Ni(COD)_2$	**L1**	TMSCl	CPME	29
	$Ni(COD)_2$	**L1**	TMSCl	THF	42
	$NiBr_2$•dme	**L1**	TMSCl	CPME	34
	$Ni(acac)_2$	**L1**	TMSCl	THF	31

Significant enhancements in outcome were observed at the third iteration. Similar to the results in Figure 2A, changing only the nickel source from $NiBr_2$ to $NiCl_2$ resulted in 6% increase in yield (Batch 3 entry 2 vs. Batch 2 entry 6) compared to the best result in the previous batch. Further changing the solvent from THF to CPME resulted in an additional 9% increase (first row). In all, a modest increase of 3% yield compared to the best condition identified during source data collection was achieved.

The plot below shows the descriptors that were used by RFCs trained at each iteration. The source model is almost identical to that used in the previous case study, emphasizing the importance of the ligand, as shown by the highest purple bar. The blue bars correspond to the model trained on the 12 reactions collected during the source dataset curation that screened nickel sources and ligands, adding more weight to the ligand and the nickel source. The teal bars show the information acquired from reactions in Table S16, reinforcing additive and mostly solvent, the two reagent classes that the model has not learned up to this point (very low or no purple or blue bars). The reactions in the second iteration (Table S17) further supplement the model with knowledge on additives. This completes its understanding of all reaction components in the target reaction space, resulting in identifying a reaction condition giving meaningfully increased yield in the third iteration (Table S18).

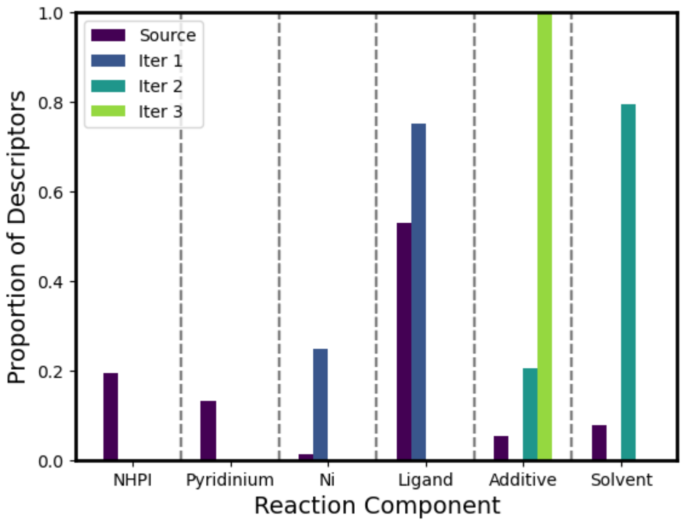

Figure S16. Descriptors used by source and target RFCs at different iterations.

Lastly, as an evaluation of the model's consistent suggestion of **L1** as the ligand, six ligands, as suggested by the model, were evaluated while other reaction conditions were fixed ($NiCl_2$•dme as nickel source, TMSCl as additive and CPME as solvent). Interestingly, most selected ligands had electron withdrawing substituents (including **L-S20** which was not included in the source dataset), in line with the qualitative conclusion drawn from the previous study that determined **L1** was optimal. However, none of the six ligands resulted in comparable yields to **L1** as shown in Figure S17. Although the possibility that the global optimal reaction condition may use a ligand other than **L1** cannot be ruled out, this supports the efficacy of ATL fixing the reagent class that is deemed most important by the source model – in this case, the ligand – in early iterations.

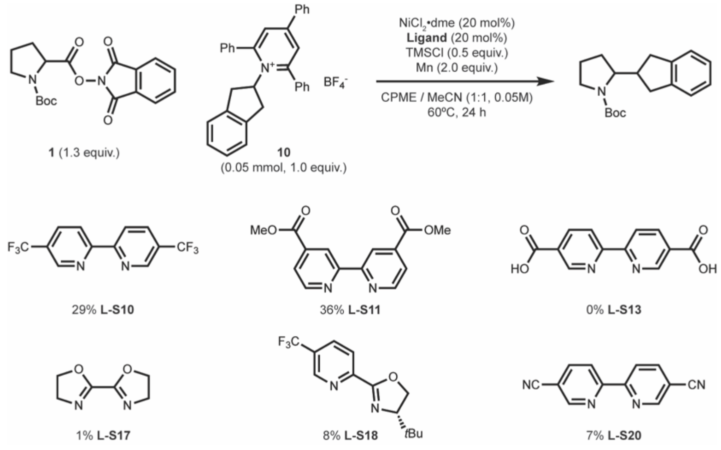

Figure S17. Results of a screen that varies only in ligand, as recommended by the latest ATL model. Assay yields are shown.

After confirming that the reaction is reproducible with CPME (which was problematic for the previous case study) at a larger 0.15 mmol scale (Entry 1, Table S19), it was subjected to stoichiometry studies for further optimization. Increasing the equivalents of **1** from 1.3 to 2.0 resulted in a modest 4% yield (Entry 7, Table S19). No other modification resulted in further improvements.

Table S19. Further optimization of continuous variables at 0.15 mmol scale. Bold values correspond to those different from the ATL condition. Isolated yields are shown except for values in parentheses. [a]) Average of two runs (49% and 47% yield). [b]) $NiBr_2$•dme and 1,4-dioxane instead of $NiCl_2$•dme and CPME were used, respectively (i.e., categorical variables of previous report).

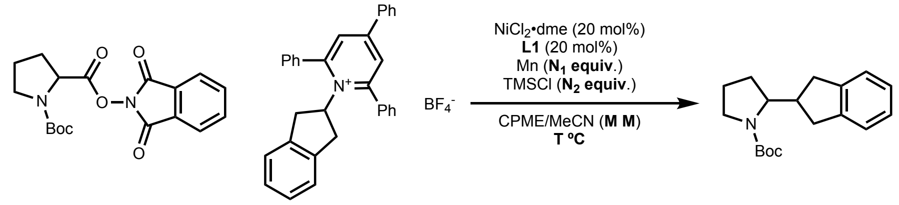

1 **10** **9**

Entry	Limiting reagent	other reagent equiv.	Catalyst loading (%)	N_1	N_2	M	T (°C)	Yield (%)
1	**10**	1.3	20	2	0.5	0.05	60	48[a]
2	**10**	1.3	20	2	**0**	0.05	60	41
3	**10**	1.3	20	2	0.5	0.05	**50**	45
4	**10**	1.3	20	**3**	0.5	0.05	**50**	43
5	**10**	1.3	20	2	**0.25**	0.05	60	40
6	**10**	1.3	20	2	**1.0**	0.05	60	36
7	**10**	**2.0**	20	2	0.5	0.05	60	52
8	**1**	1.3	20	2	0.5	0.05	60	31
9	**10**	1.3	20	2	0.5	**0.0375**	60	45
10	**10**	**2.0**	20	2	0.5	**0.0375**	60	(37)
12	**10**	**2.0**	**10**	**3**	0.5	**0.0375**	60	45
13	**10**	**2.0**	**10**	2	**0.25**	**0.0375**	60	46
14[b]	**10**	**2.0**	**10**	2	**0**	**0.0375**	60	48

9) Case study 4: BocAla-NHPI (11) + Indan-2-yl pyridinium (10)

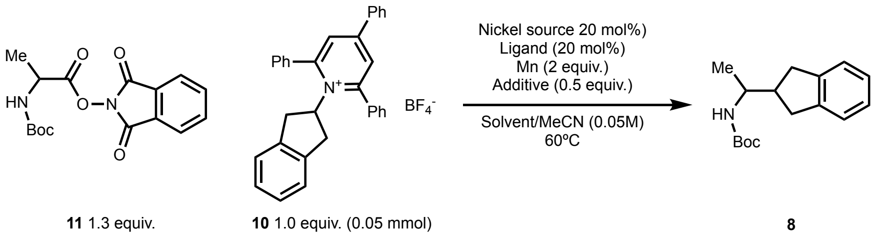

Along with **14**, the formation of **8** presents a challenge: bringing together an acyclic secondary alkyl moiety with a cyclic secondary moiety. This is exemplified by the 13% assay yield achieved with the previous reaction condition as in Figure 5A entry 1.

For training the 100 source models, the sets of reactions collected in case studies 1 and 2 were combined to the original source data. In addition, since the portion of reactions between **11** and **10** in the source dataset is large (approximately 50%), a separate preliminary target model was trained on these reactions. For this case study, each RFC voted for 12 reactions and the top-6 reactions were conducted at 0.05 mmol scale in 4 mL vials as reaction vessels.

After combining the 100 source models with 100 preliminary target models, the reactions below were recommended as the first batch (Table S20). KBr was selected as the additive throughout, again, due to its positive impact in case study 2. THF was likely selected as a solvent (along with dioxane) due to its success in coupling **11** with **12**. All nickel sources were surveyed. Along with these suggested reactions, a reaction with the previously reported condition was conducted, returning 13% assay yield (entry 1), setting the threshold for 'positive' results. Two reaction conditions with different pairs of nickel source and solvent showed modest improvements (entries 2 and 5).

Table S20. Results of 0.05 mmol scale experiments in the first ATL iteration.

Entry	Description	Ni	Ligand	Additive	Solv.	Assay Yield (%)
1	control	$NiBr_2$•dme	**L1**	None	Dioxane	13
2	ATL Batch 1	$NiCl_2$•dme	**L1**	KBr	Dioxane	15
3		$NiBr_2$•dme	**L1**	KBr	Dioxane	11
4		$NiBr_2$•dme	**L1**	KBr	THF	7
5		NiI_2	**L1**	KBr	THF	20
6		$Ni(COD)_2$	**L1**	KBr	THF	8
7		$Ni(acac)_2$	**L1**	KBr	THF	7

These results were used to train 100 RFCs, each with 15 decision trees of depth 1. After updating the previous ensemble of RFCs with the new target RFCs, the next set of six reactions to conduct was collected. Despite observation of positives in both dioxane and THF in the first iteration, the solvent was fixed to dioxane in the subsequent recommendations. The nickel source was mostly NiI_2 with one entry with $NiCl_2$•dme, reflecting the entries that gave positives from the previous batch of reactions. Similar to previous observations, a variety of additives were queried to be screened.

Table S21. Results of 0.05 mmol scale experiments in the second ATL iteration.

Entry	Description	Ni	Ligand	Additive	Solv.	Assay Yield (%)
1	ATL Batch 2	$NiCl_2$•dme	**L1**	KCl	Dioxane	25
2		NiI_2	**L1**	KCl	Dioxane	12
3		NiI_2	**L1**	KBr	Dioxane	10
4		NiI_2	**L1**	KI	Dioxane	17
5		NiI_2	**L1**	TBABr	Dioxane	3
6		NiI_2	**L1**	$MgBr_2$	Dioxane	24

In the second batch of reactions, three positives were observed (entries 1, 4 and 6) with two of them showing higher assay yields compared to the highest observed in the previous iteration (24 and 25% vs. 20%).

A set of 100 target RFCs trained on the reactions in table S21 was trained and used to update the previous model. The last set of reactions recommended (similar to the

previous batch) $NiCl_2$•dme and NiI_2 nickel sources, multiple additives and mostly dioxane as solvent, presumably due to the previous success.

Table S22. Results of 0.05 mmol scale experiments in the third ATL iteration.

Entry	Description	Ni	Ligand	Additive	Solv.	Assay Yield (%)
1	ATL Batch 3	$NiCl_2$•dme	**L1**	KI	Dioxane	14
2		$NiCl_2$•dme	**L1**	$MgBr_2$	Dioxane	37
3		$NiCl_2$•dme	**L1**	TBAI	Dioxane	6
4		$NiCl_2$•dme	**L1**	NaCl	Dioxane	12
5		NiI_2	**L1**	$MgCl_2$	Dioxane	14
6		$NiCl_2$•dme	**L1**	KI	CPME	12

Consistent with previous test cases, the best result is observed in the third iteration, returning a significantly improved assay yield of 37% (entry 2).

The source model uses ligand descriptors the most to classify reaction outcome (purple bars). The blue bars show the descriptors learned from the reactions that were a part of the source dataset which screened through ligands, additives, and solvents. Again, ligand plays a critical role along with solvent features. The combined information learned by the combined model lacks understanding of how nickel source and additives impact reaction outcome. Models trained on reaction data from Table S20 (teal bars) and S21 (yellow green bars) supplement the model's knowledge of how different reaction components impact reaction outcome. Similar to previous case studies, the third iteration, which contains the best yield, is suggested by the models that use features of all reaction components.

Lastly, the reaction was scaled up to 0.15 mmol to conduct further optimization of continuous variables (Table S23). At this scale, the previous best condition returned 10% yield (Entry 1) while the ATL-identified condition gave an improved, average 21% yield from two trials (Entry 2). While lowering the reaction temperature to 50 ºC enhanced the yield to 33% (Entry 3), other modifications such as increasing the equivalents of Mn, altering additive amounts and reactant stoichiometry did not have a positive impact.

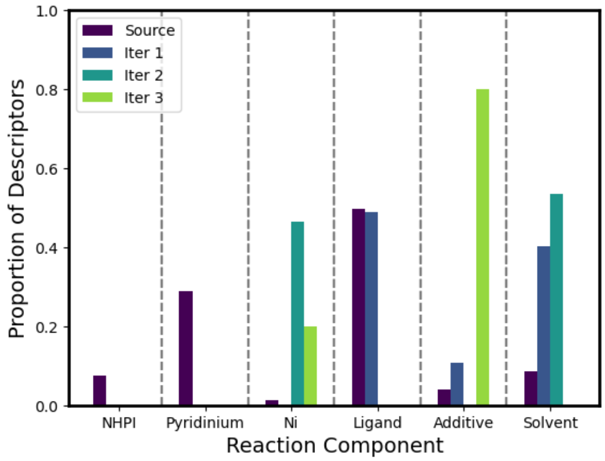

Figure S18. Descriptors used by source and target RFCs at different iterations.

Table S23. Further optimization of continuous variables at 0.15 mmol scale. Bold values correspond to those different from the previous report. Isolated yields are shown. [a]) Previous reaction condition with $NiBr_2$•dme and no $MgBr_2$. [b]) Average of two runs (22% and 20% yield). [c]) Assay yield; not further isolated.

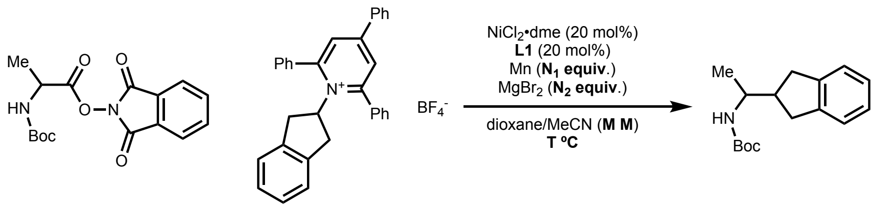

11 **10** **8**

Entry	Limiting reagent	other reagent equiv.	N_1	N_2	M	T	Yield (%)
1[a]	**10**	1.3	2	0	0.05	60	10
2	**10**	1.3	2	**0.5**	0.05	60	21[b]
3	**10**	1.3	2	0	0.05	60	11
4	**10**	1.3	2	**0.25**	0.05	60	15
5	**10**	1.3	2	**1.0**	0.05	60	23
6	**10**	**2.0**	2	0.5	0.05	60	15[c]
7	**11**	1.3	2	0.5	0.05	60	23
8	**10**	1.3	2	0.5	**0.0375**	60	22
9	**10**	1.3	2	0.5	0.05	**50**	33
10	**10**	1.3	**3**	0.5	0.05	**50**	30

10) Retrospective analysis

With the data collected up to this point, we sought to perform a retrospective analysis to compare ATL against baseline strategies such as random selection, transfer learning (TL; source model without any updates through active learning) and Bayesian optimization (EDBO developed by the Doyle group,[2] was used). Since reactions of **11**+**10** were conducted the most and spanned conditions selected by both chemists and ATL, this substrate pair was the most appropriate to use as the target. Accordingly, a problem of identifying the highest yielding reaction condition (37% yield) among 98 choices with a batch size of six was set. All reactions of **1**+**2** and **1**+**10** (total 102 datapoints) were used as source data where applicable.

Before comparing the iterative exploration of different strategies, the influence of hyperparameter combinations on source model transferability was evaluated. Specifically, a source model was trained on the 102 source reactions and used to predict the results of the 98 target reactions without any modification. Consistent with our previous observation with Pd-catalyzed C–N coupling reactions,[7] with a sufficient number of decision trees, the best target ROC-AUC score is achieved by limiting the maximum depth to one (Figure S19). This supports the importance of source model simplification for maximizing its transferability to the target domain.

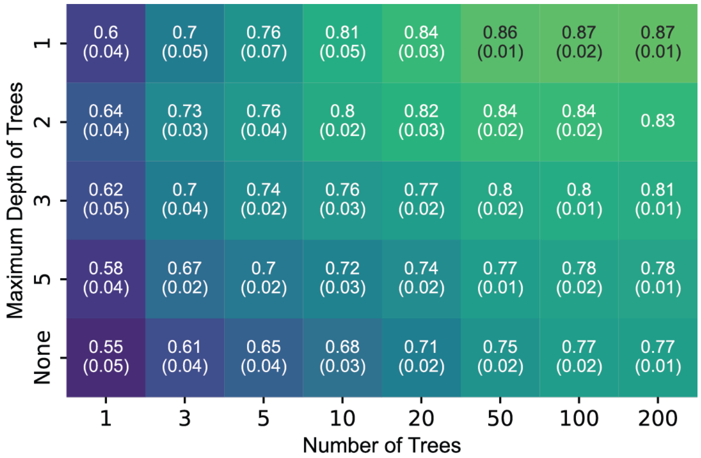

*Figure S19. Heatmap of ROC-AUC scores of predictions on 98 target reactions involving **11**+**10** with source models with different hyperparameter combinations trained on 102 reactions of **1**+**2** and **1**+**10**.*

With this result in hand, we sought to simulate the iterative exploration. For ATL and TL, the source model training procedure used in previous case studies (100 source

models of 100 decision trees of depth one) was employed. For ATL, this ensemble of source RFCs was iteratively updated with 100 target RFCs of 15 decision trees of depth one. Votes were collected up to 12 reactions and the top six were simulated as selections. One ensemble, each of ATL and TL, was evaluated. On the other hand, for random selection and EDBO, simulations were conducted 50 times. Default parameters were used for EDBO.[2]

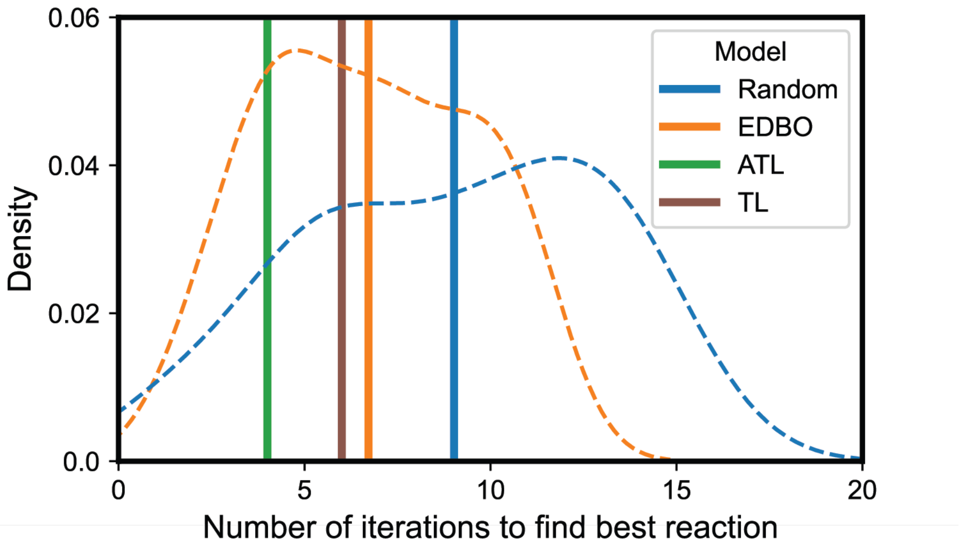

Figure S20. Number of iterations each model took to find the best yielding reaction out of 98 choices. Dotted lines correspond to kernel density estimates from 50 iterations of random selection (blue) and EDBO (orange), respectively. Since ATL and TL involved an ensemble of 100 models, only one iteration was evaluated and therefore no equivalent curves are shown. Solid vertical lines correspond to the average number of iterations each strategy needed to take to find the best reaction condition.

ATL was able to identify the best reaction condition in its fourth batch of experiments (Figure S20, green line). TL, which sequentially evaluates the static predictions of the source model (same as that used for ATL), on the other hand, examined this condition in the sixth iteration (brown line). In contrast, for EDBO and random selection, the average number of iterations it took to identify the same reaction across the 50 model instances were 6.72 and 9.02 respectively (orange and blue solid lines, respectively). This result suggests the efficiency of ATL. In particular, its comparison against TL highlights the importance of model adaptation to the target reactivity through active learning (ATL vs. TL).

11) Model interpretation through Shapley value analysis

With the accumulated dataset in hand, we investigated whether a predictive model could provide insight into aspects that impact the yield of this transformation.

First, the reactions of all five substrate pairs collected up to this point were combined to train a random forest classifier. A model was trained by searching through hyperparameters "n_estimators" $\in$ {5, 10, 25, 100} and "max_depth" $\in$ {1, 3, 5, None} with grid search CV. The resulting model, with 100 decision trees and maximum depth of 5, scored a ROC-AUC=0.836 under leave-one-substrate-pair-out CV. Impact of each descriptors on the model's prediction was then inspected through Shapley value analysis.[8]

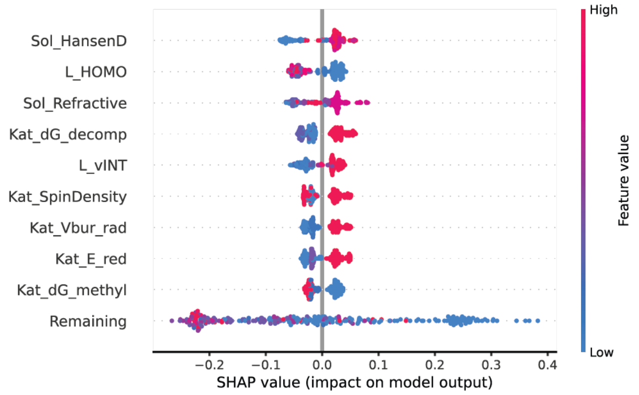

Figure S21. Shapley additive explanations (SHAP) for interpreting importance of each feature for predicting the yields of five substrate pairs considered in this study with a random forest regressor.

When predicting reaction outcomes of all five substrate pairs, the degree of impact between descriptors making the largest contributions are not significantly different (Figure S19, the span of the swarm points except the bottom row). This might be due to the subtle response of the reaction outcome to the physical features of these reagent components, namely solvent, ligand and activated amine.

Examining two solvent descriptors show solvents with high Hansen dispersion parameters (top row) and intermediate refractive index values (third row) help reaction performance. Combined, this implies the importance of pinpointing solvents within a narrow range of physical features. This is in line with our observation that enhancements were shown only with dioxane and CPME as co-solvents with MeCN.

In terms of ligands, the ligated Ni(I)–Cl complex having low HOMO values seemed necessary but not sufficient (the left side of the second row is a mix of red and blue dots). Sorting the ligands by this value places ligands with electron withdrawing substituents on the top, putting **L1** second (followed by 5,5'-dicyano-2,2'-bipyridine, which showed low activity, possibly due to the nitrogen being to electron poor to bind to Ni). Whether the ligand has high intensity of the Ni(I)–Cl bond vibration (fourth row) seems to help making the decision, with **L1** having the highest value among others.

The characteristic of the activated amine is another main contributor to the outcome. Among them, the most influential descriptor is 'Kat_dG_decomp' (fourth row) which corresponds to the free energy change when the reduced pyridinium salt undergoes deamination to form the corresponding alkyl radical. The swarmplot shows that higher 'Kat_dG_decomp' values lead to better outcomes. However, higher values come from the secondary alkyl amines **10** and **13**, which at first glance seems unintuitive because these substrates give lower yields compared to benzylic amines **2** and **12**.[8] We note that the 'model output' as noted by the label of the x-axis is classification result which compares the performance against the previously reported reaction condition, not raw yields.

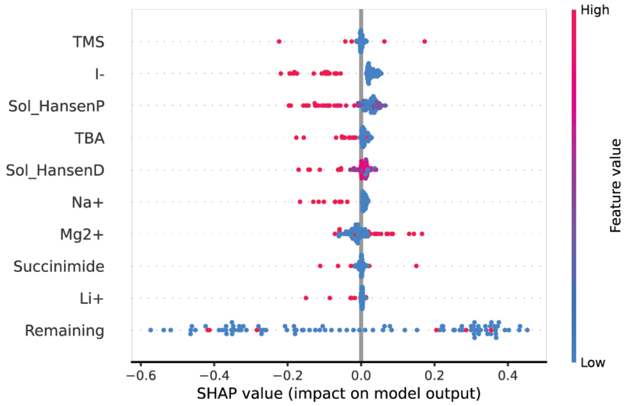

*Figure S22 . Shapley additive explanations (SHAP) for interpreting importance of each feature for predicting the yields of forming **8** with a random forest regressor.*

Next, the same analysis was applied to two substrate pairs with the largest number of reactions. Similar to the procedure above, using all reactions that form **8** as product, a classifier was trained and was further subject to analysis (Figure S20). For this reaction between **11** and **10**, additives seem to play an impactful role. Interestingly, most of ions show up with negative effects. For example, the use of iodide, tetrabutyl ammonium,

sodium or lithium cations seem detrimental as the red dots have negative SHAP values. Other additive ions that appear as one of the nine most contributing descriptor (e.g., trimethylsilyl or Mg^{2+}, first and seventh row, respectively) show a more subtle effect, where red dots are spread across both positive and negative SHAP values.

Lastly, the analysis on a classifier predicting the reactivity of **14** is shown in Figure S21. In contrast to the previous example, the impact of solvents is dominantly high. Interestingly, however, the trend in the refractive index (second row) and Hansen D (seventh row) is opposite from that in Figure S19, supporting the importance of tailoring an effective solvent environment depending on the substrates. While other factors had relatively low influence on the reaction outcome, additives' anions seem to play a role (c.f., both cations and anions were important in Figure S20). Positive SHAP values are involved with bromide additives, many of which improved yield in our studies. On the other hand, negative SHAP values are assigned to chloride additives.

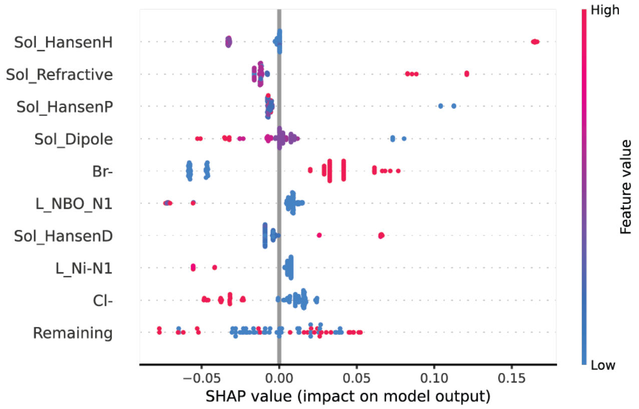

*Figure S23. Shapley additive explanations (SHAP) for interpreting importance of each feature for predicting the yields of forming **14** with a random forest regressor.*

12) Synthetic Procedures

Procedures for the synthesis of alkyl pyridinium salts

General Procedure A

In a round-bottom flask equipped with a magnetic stir bar, triphenylpyrylium tetrafluoroborate (1.0 equiv.) was dissolved in ethanol (1.0 M). Amine (1.2 equiv.) was then added while stirring. The reaction was heated to 90 ºC for 5 h and then cooled to room temperature. The reaction was concentrated *in vacuo* then diluted in DCM. The organic layer was washed with water (×5). The combined aqueous layer was extracted with DCM (×3). The combined organic layer was dried over Na_2SO_4 and concentrated *in vacuo*. The resulting crude mixture was dissolved in 3 mL DCM and was added dropwise to 50 mL Et_2O and stirred vigorously for 2 h which then the solid was filtered. If desired product does not precipitate, column chromatography was conducted using 2% acetone in DCM to remove impurities, followed by 10% acetone in DCM to isolate product. The yields are unoptimized.

General Procedure B

A round-bottom flask equipped with a magnetic stir bar was charged with triphenylpyrylium tetrafluoroborate (1.0 equiv.) and amine (1.0 equiv.). DCM (0.5 M) along with powdered 4 Å molecular sieves (~500 mg/mmol) were added. Triethylamine (2.0 equiv.) was added and stirred for 20 min followed by addition of acetic acid (2.0 equiv.). The reaction was stirred at room temperature overnight. The mixture was filtered over a pad of celite to remove molecular sieves and eluted with additional DCM. The resulting solution was washed with 1 M HCl, sat. $NaHCO_3$ (aq.), sat. NaCl (aq.), which was then dried over Na_2SO_4 and concentrated *in vacuo*. The resulting crude reaction mixture was dissolved in 10 mL DCM and was added dropwise into 100 mL Et_2O at room temperature and stirred vigorously for 2 h. The resulting solid was filtered, washed with cold Et_2O and dried under vacuum. The yields are unoptimized.

1-Benzyl-2,4,6-triphenylpyridin-1-ium tetrafluoroborate **2**

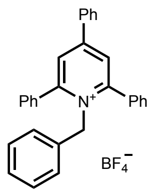

Following General Procedure A, using 4.00 g of triphenylpyrilium tetrafluoroborate, yields **2** (4.4 g, 90%) as a white solid. **^{1}H NMR (500 MHz, $CDCl_3$)** δ 7.95 (s, 2H), 7.82 (d, *J* = 6.8 Hz, 2H), 7.65 (d, *J* = 6.8 Hz, 4H), 7.61–7.42 (m, 9H), 7.19–7.07 (m, 3H), 6.47 (d, *J* = 7.5 Hz, 2H); **^{13}C NMR (126 MHz, $CDCl_3$)** δ 157.8, 156.5, 134.4, 134.0, 132.9, 132.5, 131.1, 130.0, 129.3, 129.2, 128.9, 128.4, 128.3, 126.7, 126.4, 58.37. **LRMS (ESI)**: m/z calculated $C_{30}H_{24}N$ $[M–BF_4]^+$ 398.2, found 398.5. All data match that previously reported in the literature.[9]

1-(4-Methylbenzyl)-2,4,6-triphenylpyridin-1-ium tetrafluoroborate **12**

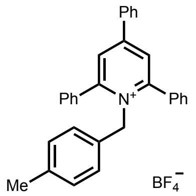

Following General Procedure A, using 3.00 g of triphenylpyrilium tetrafluoroborate, yields **12** (3.3 g, 88%) as a white solid. **^{1}H NMR (500 MHz, $CDCl_3$)** δ 7.90 (s, 2H), 7.80–7.76 (m, 2H), 7.64 (d, *J* = 6.9 Hz, 4H), 7.58–7.42 (m, 9H), 6.89 (d, *J* = 7.9 Hz, 2H), 6.32 (d, *J* = 8.0 Hz, 2H), 5.71 (s, 2H), 2.22 (s, 3H); **^{13}C NMR (126 MHz, $CDCl_3$)** δ 157.7, 156.3, 138.3, 133.9, 132.9, 132.5, 131.2, 131.0, 129.9, 129.6, 129.3, 129.2, 128.3, 126.7, 126.3, 58.2, 21.1. **LRMS (ESI)**: m/z calculated $C_{31}H_{26}N$ $[M–BF_4]^+$ 412.2, found 412.5. All data match that previously reported in the literature.[9]

1-(2,3-dihydro-1H-inden-2-yl)-2,4,6-triphenylpyridin-1-ium tetrafluoroborate **10**

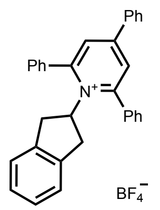

Following General Procedure B, using 4.68 g of triphenylpyrilium tetrafluoroborate, yields **10** (5.1 g, 85%) as a pale-yellow solid. **^{1}H NMR (500 MHz, $CDCl_3$)** δ 7.78 (s, 2H), 7.72–7.65 (m, 6H), 7.52–7.47 (m, 1H), 7.45–7.40 (m, 2H), 7.37–7.30 (m, 6H), 6.91 (dt, *J* = 7.3, 3.7 Hz, 2H), 6.74 (dt, *J* = 5.4, 3.4 Hz, 2H), 5.64 (tt, *J* = 10.7, 4.5 Hz, 1H), 3.69 (dd, *J* = 18.5, 4.4 Hz, 2H), 3.37 (dd, *J* = 18.5, 10.7 Hz, 2H); **^{13}C NMR (126 MHz, $CDCl_3$)** δ 157.8, 155.2, 139.3, 134.2, 133.5, 132.0, 130.7, 129.7, 129.6, 128.7, 128.3, 128.2, 126.8, 124.1, 67.7, 41.7. **LRMS (ESI)**: m/z calculated $C_{32}H_{26}N$ $[M–BF_4]^+$ 424.2, found 424.5. All data match that previously reported in the literature.[10]

1-(1-(2,6-dimethylphenoxy)propan-2-yl)-2,4,6-triphenylpyridin-1-ium tetrafluoroborate **13**

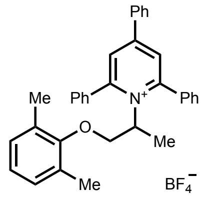

Following General Procedure B, using 3.96 g of triphenylpyrilium tetrafluoroborate, yields **13** (3.9 g, 70%) as a pale-yellow solid. **^{1}H NMR (500 MHz, $CDCl_3$)** δ 8.01–7.67 (m, 7H), 7.63–7.51 (m, 7H), 7.48 (t, *J* = 7.8 Hz, 2H), 6.95–6.87 (m, 3H), 5.48 (h, *J* = 7.0 Hz, 1H), 4.12 (dd, *J* = 9.9, 6.7 Hz, 1H), 3.53 (dd, *J* = 9.9, 6.9 Hz, 1H), 1.92 (s, 6H), 1.54 (d, *J* = 7.2 Hz, 3H); **^{13}C NMR (126 MHz, $CDCl_3$)** δ 155.9, 154.4, 134.1, 133.7, 132.2, 131.2, 130.1, 129.7, 129.4, 129.3, 129.0, 128.5, 124.6, 73.6, 65.4, 19.4, 16.4. **LRMS (ESI)**: m/z calculated $C_{34}H_{32}NO$ $[M–BF_4]^+$ 470.3, found 470.5. All data match that previously reported in the literature.[10]

2,4,6-triphenyl-1-(4-phenylbutan-2-yl)pyridin-1-ium tetrafluoroborate **S3**

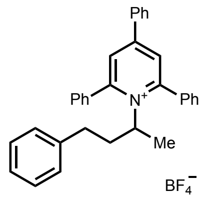

Following General Procedure B, using 4.59 g of triphenylpyrilium tetrafluoroborate, yields **S3** (4.2 g, 69%) as a pale-yellow solid. **^{1}H NMR (500 MHz, $CDCl_3$)** δ 7.71 (d, *J* = 6.8 Hz, 2H), 7.66 (s, 2H), 7.63 (d, *J* = 7.7 Hz, 2H), 7.58–7.42 (m, 7H), 7.37 (t, *J* = 7.7 Hz, 2H), 7.18 - 7.09 (m, 3H), 6.91–6.83 (m, 2H), 4.94–4.80 (m, 1H), 2.42–2.30 (m, 1H), 2.25–2.07 (m, 2H), 1.79–1.67 (m, 1H), 1.43 (d, *J* = 6.9 Hz, 3H); **^{13}C NMR (126 MHz, $CDCl_3$)** δ 157.1, 154.9, 138.9, 133.8, 133.6, 131.8, 130.7, 129.4, 128.6, 128.2, 128.0, 126.3, 65.9, 37.5, 32.3, 21.6 (3 aromatic carbon signals are not observed due to signal broadening). **LRMS (ESI)**: m/z calculated $C_{33}H_{30}N$ $[M–BF_4]^+$ 440.2, found 440.5. All data match that previously reported in the literature.[10]

1-(1-((*tert*-butyldimethylsilyl)oxy)propan-2-yl)-2,4,6-triphenylpyridin-1-ium tetrafluoroborate **S4**

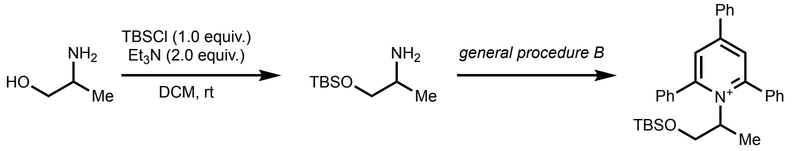

To a 50 mL flame-dried round bottom flask was added 8mL DCM, followed by 2-aminopropan-1-ol (1.0 g, 13.3 mmol) and Et_3N (3.7 mL, 2.7 g, 26.6 mmol). In a separate 25 mL round bottom flask was added chloro-*tert*-butyldimethylsilane chloride (2.01 g, 13.3 mmol), which was dissolved in 8 mL DCM. This solution was transferred dropwise to the other flask at room temperature dropwise. After stirring at room temperature overnight, sat. NH_4Cl (aq.) was added. The layers were separated, and the aqueous layer was extracted with DCM (3 × 20 mL). The combined organic layer was washed with brine and dried over Na_2SO_4, filtered, and concentrated *in vacuo*.[11] The crude mixture was used for the next step without purification, following General Procedure B. Purification with 2 and 10% acetone in DCM gives **S4** (4.4 g, 58%) as a yellow solid. **^{1}H NMR (500 MHz, $CDCl_3$)** δ 7.89–7.67 (m, 7H), 7.62–7.53 (m, 7H), 7.53–7.47 (m, 3H), 5.11 (dp, *J* = 10.0, 7.0 Hz, 1H), 3.44 (dd, *J* = 11.1, 10.0 Hz, 1H), 3.33 (dd, *J* = 11.2, 6.3 Hz, 1H), 1.45 (d, *J* = 7.1 Hz,

3H), 0.77 (s, 9H), -0.05 (d, *J* = 11.1 Hz, 6H); **^{13}C NMR (126 MHz, $CDCl_3$)** δ 158.1, 155.5, 134.2, 133.9, 132.1, 131.1, 129.8, 129.3, 128.8, 128.4, 127.8, 67.5, 64.3, 25.8, 18.3, 18.0, -5.3; **HRMS (ESI)**: calculated $C_{32}H_{38}NOSi$ $[M]^+$ 480.2723, found 480.2712.

Procedure for the synthesis of NHPI esters

General Procedure C

To a flame-dried round-bottom flask with a magnetic stir bar was added N-hydroxyphthalimide (1.1 equiv.), carboxylic acid (1.0 equiv.), which was then dissolved in DCM (0.2 M). While stirring, *N,N*-dimethyl-4-aminopyridine (0.1 equiv.) was added, which was then stirred at room temperature for 5 minutes. Then, diisopropylmethanedimine (1.1 equiv.) was added via syringe dropwise upon which the reaction mixture's color changes. The reaction mixture was stirred at room temperature overnight. After confirming the starting material was fully consumed, the reaction mixture was directly poured onto column chromatography, which with a solution of EtOAc in Hex as eluent isolates the product. The resulting solid or gel can be recrystallized over methanol for higher purity. The yields are unoptimized.

1-(*tert*-Butyl) 2-(1,3-dioxoisoindolin-2-yl) pyrrolidine-1,2-dicarboxylate **1**

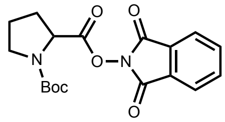

Following General Procedure C, using 3.00 g of *N*-Boc-proline, yields **1** (4.7 g, 93%) as white solid. **^{1}H NMR (500 MHz, $CDCl_3$)** δ 7.89–7.82 (m, 2H), 7.80–7.72 (m, 2H), 4.63–4.54 (dd, *J* = 8.7, 3.7 Hz, 1H), 3.65–3.56 (m, 1H), 3.51–3.43 (dt, *J* = 10.5, 7.5 Hz, 1H), 2.48–2.29 (m, 2H), 2.13–1.90 (m, 2H), 1.50 (s, 9H); **^{13}C NMR (126 MHz, $CDCl_3$)** δ 169.8, 161.8, 153.6, 134.9, 129.1, 124.0, 81.3, 57.3, 46.4, 31.6, 28.2, 23.7. **LRMS (ESI)**: m/z calculated $C_{13}H_{12}N_2O_4$ $[M\text{-}Boc+H]^+$ 261.1, found 261.3. All data match that previously reported in the literature.[9]

1,3-Dioxoisoindolin-2-yl (*tert*-butoxycarbonyl)alaninate **11**

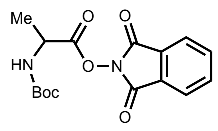

Following General Procedure C, using 1.91 g of *N*-Boc-alanine, yields **11** (2.8 g, 82%) as white solid. **^{1}H NMR (500 MHz, $CDCl_3$)** δ 7.96–7.87 (dd, *J* = 5.4, 3.1 Hz, 2H), 7.86–7.77 (m, 2H), 5.15–5.00 (br s, 1H), 4.86–4.70 (br s, 1H), 1.70–1.59 (d, *J* = 7.3 Hz, 3H), 1.59–1.40 (s, 9H); **^{13}C NMR (126 MHz, $CDCl_3$)** δ 170.1, 161.6, 154.8, 135.0, 129.0, 124.2, 80.7, 47.8, 28.4, 22.8, 19.0. **LRMS (ESI)**: m/z calculated $C_{12}H_{10}N_2O_6$ $[M–tBu+H]^+$ 279.1, found 279.2. All data match that previously reported in the literature.[9]

Procedure for the cross-coupling reaction

General Procedure D

To a flame-dried 8 mL vial equipped with a magnetic stir bar, NHPI ester (0.20 mmol, 1.3 equiv.), Katritzky salt (0.15 mmol, 1.0 equiv.) were added, then brought into the glovebox. In the glovebox, $NiBr_2$•glyme (9.3 mg, 0.030 mmol, 20 mol%), and **L1** (8.8 mg, 0.030 mmol, 20 mol%) were weighed into the reaction vial. 1,4-Dioxane (1.5 mL) was added, and the mixture was stirred for 10 minutes at room temperature in the glovebox. In the meanwhile, additive (if used, 0.075 mmol, 0.5 equiv.) and Mn (16.5 mg, 0.30 mmol, 2.0 equiv.) was weighed. These were added, followed by the addition of MeCN (1.5 mL). The vial was brought out of glovebox, sealed with parafilm, followed by electrical tape, and heated at 60 °C for 24 hours stirring at 900 rpm. After the reaction was cooled down to room temperature, it was eluted through a pad of silica gel (which needs to be prepared in a wide enough column to prevent clogging; specifically, a plug approximately 2 inch high in a 30 mm diameter column was used) with EtOAc. The crude reaction mixture was concentrated *in vacuo* and the residue was purified by column chromatography (EtOAc / hexanes or Et_2O / toluene) to give isolated product.

tert-butyl (1-(*p*-tolyl)propan-2-yl)carbamate **7**

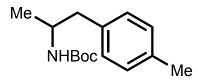

Following General Procedure D, using 65.2 mg **11** and 74.9 mg **12**, gives an average 55% yield of **7** (21.0 mg, 0.084 mmol, 56% / 20.0 mg, 0.080 mmol, 54%) as white solid after purification with 3–8% EtOAc in hexanes. Using THF instead of 1,4-dioxane as the first solvent and increasing the equivalents of Mn from 2 to 3 resulted in an average 63% yield of **7** (24.0 mg, 0.096 mmol, 64% / 23.1 mg, 0.093 mmol, 62%). **^{1}H NMR (500 MHz, $CDCl_3$)** δ 7.10 (d, *J* = 7.9 Hz, 1H), 7.07 (d, *J* = 7.9 Hz, 1H), 4.38 (s, 1H), 3.88 (s, 1H), 2.80 (dd, *J* = 13.6, 5.4 Hz, 1H), 2.61 (dd, *J* = 13.4, 7.4 Hz, 1H), 2.32 (s, 3H), 1.43 (s, 9H), 1.07 (d, *J* = 6.6 Hz, 1H); **^{13}C NMR (126 MHz, $CDCl_3$)** δ 155.3, 135.9, 135.2, 129.5, 129.1, 79.2, 47.6, 42.7, 28.6, 21.2, 20.3. **HRMS (ESI)**: calculated $C_{11}H_{16}NO_2$ $[M\text{-}Boc+H]^+$ 194.1181, found 194.1179.

tert-butyl (S)-2-(2,3-dihydro-1H-inden-2-yl)pyrrolidine-1-carboxylate **9**

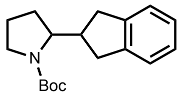

Following General Procedure D, using 70.3 mg **1** and 76.7 mg **10**, gives an average 37% yield of **9** (16.0 mg, 0.056 mmol, 37% / 16.1 mg, 0.056 mmol, 37%) as yellow oil after purification with 3–10% EtOAc in hexanes. Using $NiCl_2$•dme, cyclopentyl methyl ether instead of $NiBr_2$•dme and dioxane, respectively, along with TMSCl as additive (9.5 μL, 0.075 mmol, 0.5 equiv.) affords average 48% yield of **9** (21.0 mg, 0.073 mmol, 49% / 20.2 mg, 0.070 mmol, 47%).

^{1}H NMR (500 MHz, $CDCl_3$) δ 7.18 (d, *J* = 5.4 Hz, 1H), 7.11 (d, *J* = 4.2 Hz, 1H), 4.01 (br s, 1H), 3.54 (br s, 1H), 3.36–3.24 (m, 1H), 2.96–2.61 (m, 5H), 1.96–1.78 (ddd, *J* = 16.0, 12.0, 6.9 Hz, 2H), 1.78–1.65 (br s, 2H), 1.53–1.39 (s, 9H); **^{13}C NMR (126 MHz, $CDCl_3$)** δ 155.3, 143.0, 126.3, 124.5, 79.5, 60.6, 46.4, 44.0, 36.6, 35.7, 28.7, 23.2. **LRMS (ESI)**: m/z calculated $C_{14}H_{17}NO_2$ $[M\text{–}tBu+H]^+$ 232.1, found 232.3. All data match that previously reported in the literature.[12]

tert-butyl (1-(2,3-dihydro-1H-inden-2-yl)ethyl)carbamate **8**

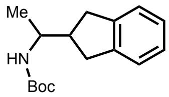

Following General Procedure D, using 65.2 mg **11** and 76.7 mg **10**, gives 10% yield of **8** (4.1 mg, 0.016 mmol) as yellow oil after purification with 0–3% Et_2O in toluene. Using $NiCl_2$•dme instead of $NiBr_2$•dme along with $MgBr_2$ as additive (13.8 mg, 0.075 mmol, 0.5 equiv.) affords 33% yield of **8** (13.0mg, 0.050 mmol) after purification.

^{1}H NMR (500 MHz, $CDCl_3$) δ 7.20–7.16 (m, 1H), 7.13 (dd, *J* = 5.5, 3.2 Hz, 1H), 4.45 (br s, 1H), 3.82 (q, *J* = 7.6 Hz, 1H), 2.99 (ddd, *J* = 20.5, 15.7, 8.2 Hz, 2H), 2.75 (ddd, *J* = 24.9, 15.8, 9.2 Hz, 1H), 2.47 (h, *J* = 8.8 Hz, 1H), 1.45 (s, 9H), 1.45 (s, 3H); **^{13}C NMR (126 MHz, $CDCl_3$)** δ 155.6, 143.0, 126.4, 124.5, 79.2, 50.2, 46.5, 36.3, 28.6, 20.2. **HRMS (ESI)**: calculated $C_{12}H_{16}NO_2$ $[M-C_4H_9+H]^+$ 206.1181, found 206.1174.

tert-butyl 2-(1-(2,6-dimethylphenoxy)propan-2-yl)pyrrolidine-1-carboxylate **14**

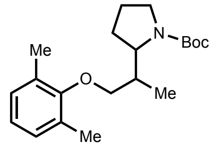

Following General Procedure D, using 70.3 mg **1** and 84.0 mg **13**, gives an 12% yield of **14** (6.2 mg, 0.018 mmol) as yellow oil after purification with 0–3% Et_2O in toluene. Using 8.9 mg, 0.075 mmol, 0.5 equiv. of KBr as additive, along with 3 equiv. of Mn at 50 ºC returns 33% yield of **14** (16.7 mg, 0.050 mmol) after purification.

^{1}H NMR (500 MHz, $CDCl_3$) Major Diastereomer δ 7.00 (d, *J* = 7.5 Hz, 2H), 6.93–6.86 (m, 1H), 4.02 (q, *J* = 5.6 Hz, 1H), 3.69 (dd, *J* = 8.7, 3.9 Hz, 1H), 3.62–3.51(m, 2H), 3.26 (dt, *J* = 13.1, 7.8 Hz, 1H), 2.66 (p, *J* = 6.6 Hz, 1H), 2.26 (s, 6H), 1.98–1.71 (m, 4H), 1.46 (s, 9H), 1.09–0.94 (m, 3H) **Minor diastereomer** δ 7.00 (d, *J* = 7.5 Hz, 2H), 6.93–6.86 (m, 1H), 3.96–3.83 (m, 1H), 3.67–3.63 (m, 1H), 3.51–3.39 (m, 2H), 3.23–3.17 (m, 1H), 2.62–2.51 (m, 1H), 2.26 (s, 6H), 1.98–1.71 (m, 4H), 1.46 (s, 9H), 1.17 (d, *J* = 6.9 Hz, 2H); **^{13}C NMR (126 MHz, $CDCl_3$)** δ 156.1, 155.0, 131.0, 128.9, 123.8, 79.4, 75.2, 60.1, 47.0, 37.0, 29.8, 28.6, 26.7, 24.0, 16.4; **HRMS (ESI)**: calculated $C_{15}H_{24}NO$ $[M-Boc+H]^+$ 234.1858, found 234.1860.

tert-butyl 2-(4-phenylbutan-2-yl)pyrrolidine-1-carboxylate **S1**

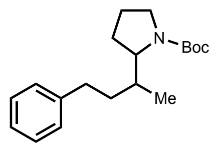

Following General Procedure D, using 70.3 mg **1** and 79.1mg **S3**, gives an 31% yield of **S1** (14.3 mg, 0.047 mmol) as yellow oil after purification with 0–3% Et_2O in toluene. Using 8.9 mg, 0.075 mmol, 0.5 equiv. of KBr as additive, along with 3 equiv. of Mn at 50 ºC returns 39% yield of **S1** (17.6 mg, 0.058 mmol) after purification.

^{1}H NMR (500 MHz, $CDCl_3$) mixture of diastereomers: δ 7.26–7.21 (m, 2H), 7.18–7.11 (m, 3H), 3.91–3.62 (m, 1H), 3.61–3.37 (m, 1H), 3.18 (dtd, *J* = 10.5, 6.6, 3.7 Hz, 1H), 2.69 (ddd, *J* = 13.6, 10.7, 5.1 Hz, 1H), 2.61–2.50 (m, 1H of major diastereomer), 2.50–2.40 (m, 1H of minor diastereomer), 2.32–1.89 (m, 1H), 1.87–1.51 (m, 6H), 1.45 (s, 9H), 0.92 (d, *J* = 6.9 Hz, 1H of minor diastereomer), 0.82 (d, *J* = 6.8 Hz, 3H of major diastereomer); **^{13}C NMR (126 MHz, $CDCl_3$)** δ 155.1 (155.2), 142.7 (143.3), 128.4 (128.5), 128.4, 125.8, 79.2 (79.0), 62.3, 61.3, 47.2 (47.6), 36.0 (36.5), 35.1, 34.1 (34.3), 28.6, 26.4 (25.6), 24.0 (23.8), 13.9 (16.7). **LRMS (ESI)**: m/z calculated $C_{15}H_{21}NO_2$ $[M–tBu+H]^+$ 248.2, found 248.3. All data match that previously reported in the literature.[12]

tert-butyl 2-(1-((*tert*-butyldimethylsilyl)oxy)propan-2-yl)pyrrolidine-1-carboxylate **S2**

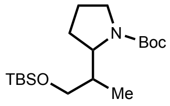

Following General Procedure D, using 70.3 mg **1** and 85.1mg **S4**, gives 23% yield of **S2** (11.7 mg, 0.034 mmol) as yellow oil after purification with 0–3% Et_2O in toluene. Using 8.9 mg, 0.075 mmol, 0.5 equiv. of KBr as additive, along with 3 equiv. of Mn at 50 ºC returns 28% yield of **S2** (14.5 mg, 0.042 mmol) after purification.

^{1}H NMR (500 MHz, $CDCl_3$) mixture of diastereomers: δ 3.86–3.73 (m, 1H), 3.60–3.33 (m, 3H), 3.27–3.16 (m, 1H), 2.22–2.12, 1.91–1.69 (m, 5H), 1.48–1.41 (m, 9H), 0.92 (d, *J* = 7.0 Hz, 3H of one diastereomer), 0.88 (s, 9H), 0.83–0.77 (m, 3H of minor diastereomer), 0.03 (s, 6H); **^{13}C NMR (126 MHz, $CDCl_3$)** δ 155.1, 79.3 (78.9), 65.4 (66.5), 60.0 (60.3), 59.0 (59.5), 46.9 (46.6), 38.7 (39.6), 28.7 (28.7), 26.1 (26.9), 23.9 (23.5), 18.4 (14.5), 11.9 (12.4), -5.29 (dd, *J* = 5.1, 3.6 Hz); **HRMS (ESI)**: calculated $C_{13}H_{30}NOSi$ $[M-Boc+H]^+$ 244.2097, found 244.2086.

13) References

1. Reker, D., Hoyt, E. A., Bernardes, G. J. L. & Rodrigues, T. Adaptive Optimization of Chemical Reactions with Minimal Experimental Information. *Cell Reports Phys Sci* **1,** 100247 (2020).

2. Shields, B. J., Stevens, J., Li, J., Parasram, M., Damani, F., Alvarado, J. I. M., Janey, J. M., Adams, R. P. & Doyle, A. G. Bayesian reaction optimization as a tool for chemical synthesis. *Nature* **590,** 89–96 (2021).

3. Christensen, M., Yunker, L. P. E., Adedeji, F., Häse, F., Roch, L. M., Gensch, T., Gomes, G. dos P., Zepel, T., Sigman, M. S., Aspuru-Guzik, A. & Hein, J. E. Data-science driven autonomous process optimization. *Commun Chem* **4,** 112 (2021).

4. Torres, J. A. G., Lau, S. H., Anchuri, P., Stevens, J. M., Tabora, J. E., Li, J., Borovika, A., Adams, R. P. & Doyle, A. G. A Multi-Objective Active Learning Platform and Web App for Reaction Optimization. *J Am Chem Soc* **144,** 19999–20007 (2022).

5. Dalton, D. M., Walroth, R. C., Rouget-Virbel, C., Mack, K. A. & Toste, F. D. Utopia Point Bayesian Optimization Finds Condition-Dependent Selectivity for N-Methyl Pyrazole Condensation. *Journal of the American Chemical Society* **146,** 15779–15786 (2024).

6. Epifanovsky, E., Gilbert, A. T. B., Feng, X., Lee, J., Mao, Y., Mardirossian, N., Pokhilko, P., White, A. F., Coons, M. P., Dempwolff, A. L., Gan, Z., Hait, D., Horn, P. R., Jacobson, L. D., Kaliman, I., Kussmann, J., Lange, A. W., Lao, K. U., Levine, D. S., Liu, J., McKenzie, S. C., Morrison, A. F., Nanda, K. D., Plasser, F., Rehn, D. R., Vidal, M. L., You, Z.-Q., Zhu, Y., Alam, B., Albrecht, B. J., Aldossary, A., Alguire, E., Andersen, J. H., Athavale, V., Barton, D., Begam, K., Behn, A., Bellonzi, N., Bernard, Y. A., Berquist, E. J., Burton, H. G. A., Carreras, A., Carter-Fenk, K., Chakraborty, R., Chien, A. D., Closser, K. D., Cofer-Shabica, V., Dasgupta, S., Wergifosse, M. de, Deng, J., Diedenhofen, M., Do, H., Ehlert, S., Fang, P.-T., Fatehi, S., Feng, Q., Friedhoff, T., Gayvert, J., Ge, Q., Gidofalvi, G., Goldey, M., Gomes, J., González-Espinoza, C. E., Gulania, S., Gunina, A. O., Hanson-Heine, M. W. D., Harbach, P. H. P., Hauser, A., Herbst, M. F., Vera, M. H., Hodecker, M., Holden, Z. C., Houck, S., Huang, X., Hui, K., Huynh, B. C., Ivanov, M., Jász, Á., Ji, H., Jiang, H., Kaduk, B., Kähler, S., Khistyaev, K., Kim, J., Kis, G., Klunzinger, P., Koczor-Benda, Z., Koh, J. H., Kosenkov, D., Koulias, L., Kowalczyk, T., Krauter, C. M., Kue, K., Kunitsa, A., Kus, T., Ladjánszki, I., Landau, A., Lawler, K. V., Lefrancois, D., Lehtola, S., Li, R. R., Li, Y.-P., Liang, J., Liebenthal, M., Lin, H.-H., Lin, Y.-S., Liu, F., Liu, K.-Y., Loipersberger, M., Luenser, A., Manjanath, A., Manohar, P., Mansoor, E., Manzer, S. F., Mao, S.-P., Marenich, A. V., Markovich, T., Mason, S., Maurer, S. A., McLaughlin, P. F., Menger, M. F. S. J., Mewes, J.-M., Mewes, S. A., Morgante, P., Mullinax, J. W., Oosterbaan, K. J., Paran, G., Paul, A. C., Paul, S. K., Pavošević, F., Pei, Z., Prager, S., Proynov, E. I., Rák, Á., Ramos-Cordoba, E., Rana, B., Rask, A. E., Rettig, A., Richard, R. M., Rob, F., Rossomme, E., Scheele, T., Scheurer, M., Schneider, M., Sergueev, N., Sharada, S. M., Skomorowski, W., Small, D. W., Stein, C. J., Su, Y.-C., Sundstrom, E. J., Tao, Z., Thirman, J., Tornai, G. J., Tsuchimochi, T., Tubman, N. M., Veccham, S. P., Vydrov, O., Wenzel, J., Witte,

J., Yamada, A., Yao, K., Yeganeh, S., Yost, S. R., Zech, A., Zhang, I. Y., Zhang, X., Zhang, Y., Zuev, D., Aspuru-Guzik, A., Bell, A. T., Besley, N. A., Bravaya, K. B., Brooks, B. R., Casanova, D., Chai, J.-D., Coriani, S., Cramer, C. J., Cserey, G., DePrince, A. E., DiStasio, R. A., Dreuw, A., Dunietz, B. D., Furlani, T. R., Goddard, W. A., Hammes-Schiffer, S., Head-Gordon, T., Hehre, W. J., Hsu, C.-P., Jagau, T.-C., Jung, Y., Klamt, A., Kong, J., Lambrecht, D. S., Liang, W., Mayhall, N. J., McCurdy, C. W., Neaton, J. B., Ochsenfeld, C., Parkhill, J. A., Peverati, R., Rassolov, V. A., Shao, Y., Slipchenko, L. V., Stauch, T., Steele, R. P., Subotnik, J. E., Thom, A. J. W., Tkatchenko, A., Truhlar, D. G., Voorhis, T. V., Wesolowski, T. A., Whaley, K. B., Woodcock, H. L., Zimmerman, P. M., Faraji, S., Gill, P. M. W., Head-Gordon, M., Herbert, J. M. & Krylov, A. I. Software for the frontiers of quantum chemistry: An overview of developments in the Q-Chem 5 package. *J Chem Phys* **155,** 084801 (2021).

7. Shim, E., Kammeraad, J. A., Xu, Z., Tewari, A., Cernak, T. & Zimmerman, P. M. Predicting reaction conditions from limited data through active transfer learning. *Chem Sci* **13,** 6655–6668 (2022).

8. Molnar, C. *Interpretable Machine Learning: A Guide for Making Black Box Models Explainable*. (2022). at <https://christophm.github.io/interpretable-ml-book/>

9. Zhang, Z. & Cernak, T. The Formal Cross-Coupling of Amines and Carboxylic Acids to Form sp3–sp3 Carbon–Carbon Bonds. *Angew. Chem. Int. Ed.* **60,** 27293–27298 (2021).

10. Douthwaite, J. L., Zhao, R., Shim, E., Mahjour, B., Zimmerman, P. M. & Cernak, T. Formal Cross-Coupling of Amines and Carboxylic Acids to Form sp3–sp2 Carbon–Carbon Bonds. *J. Am. Chem. Soc.* **145,** 10930–10937 (2023).

11. Palomo, C., Aizpurua, J. M., Balentová, E., Jimenez, A., Oyarbide, J., Fratila, R. M. & Miranda, J. I. Synthesis of β-Lactam Scaffolds for Ditopic Peptidomimetics. *Org. Lett.* **9,** 101–104 (2007).

12. Qian, D. & Hu, X. Ligand-Controlled Regiodivergent Hydroalkylation of Pyrrolines. *Angew. Chem. Int. Ed.* **58,** 18519–18523 (2019).

14) NMR Spectra

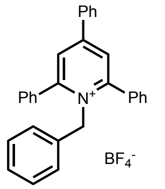

2

^{1}H NMR (500 MHz)

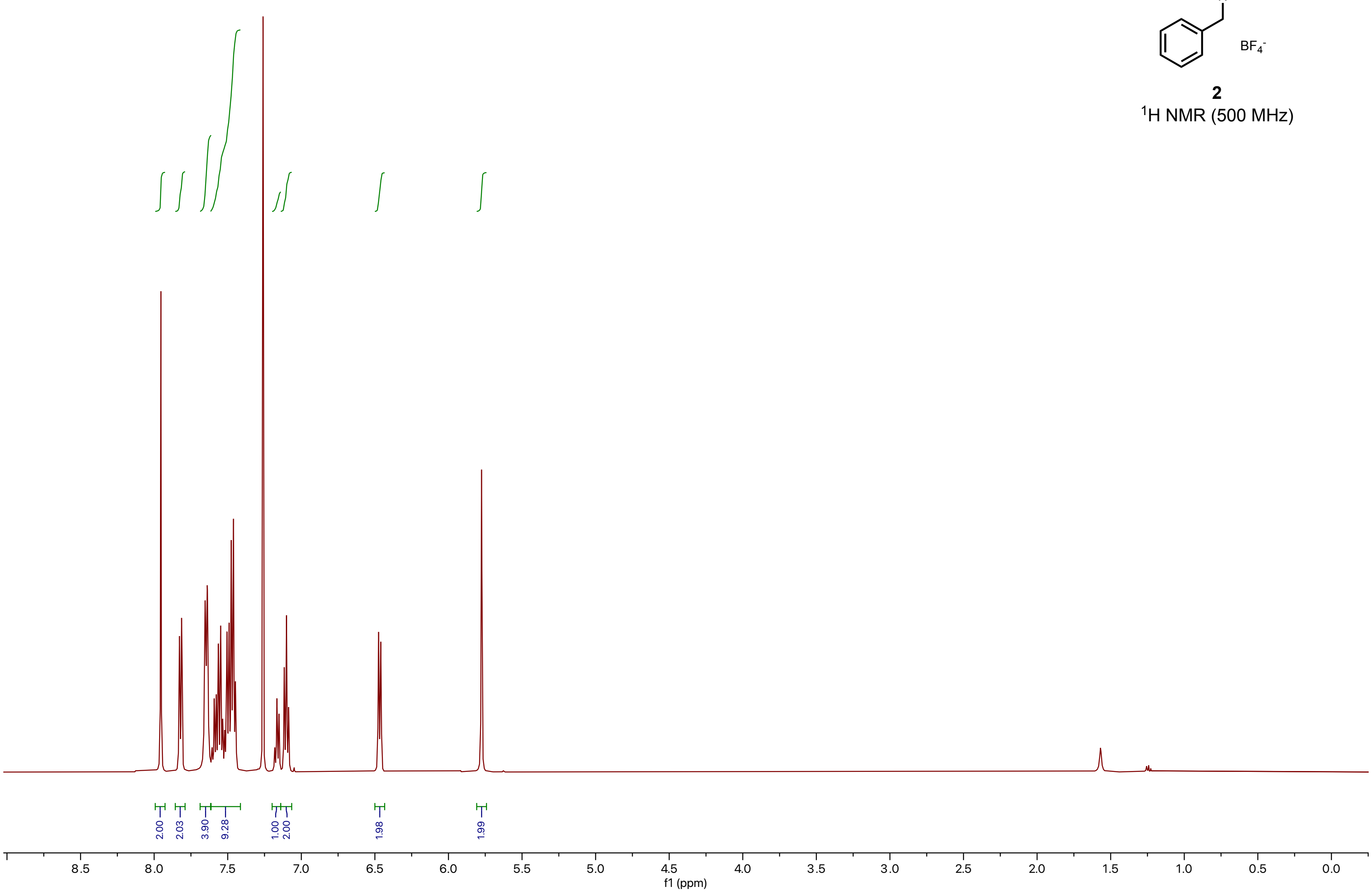

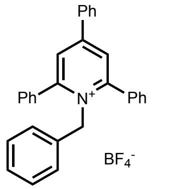

2

^{13}C NMR (126 MHz)

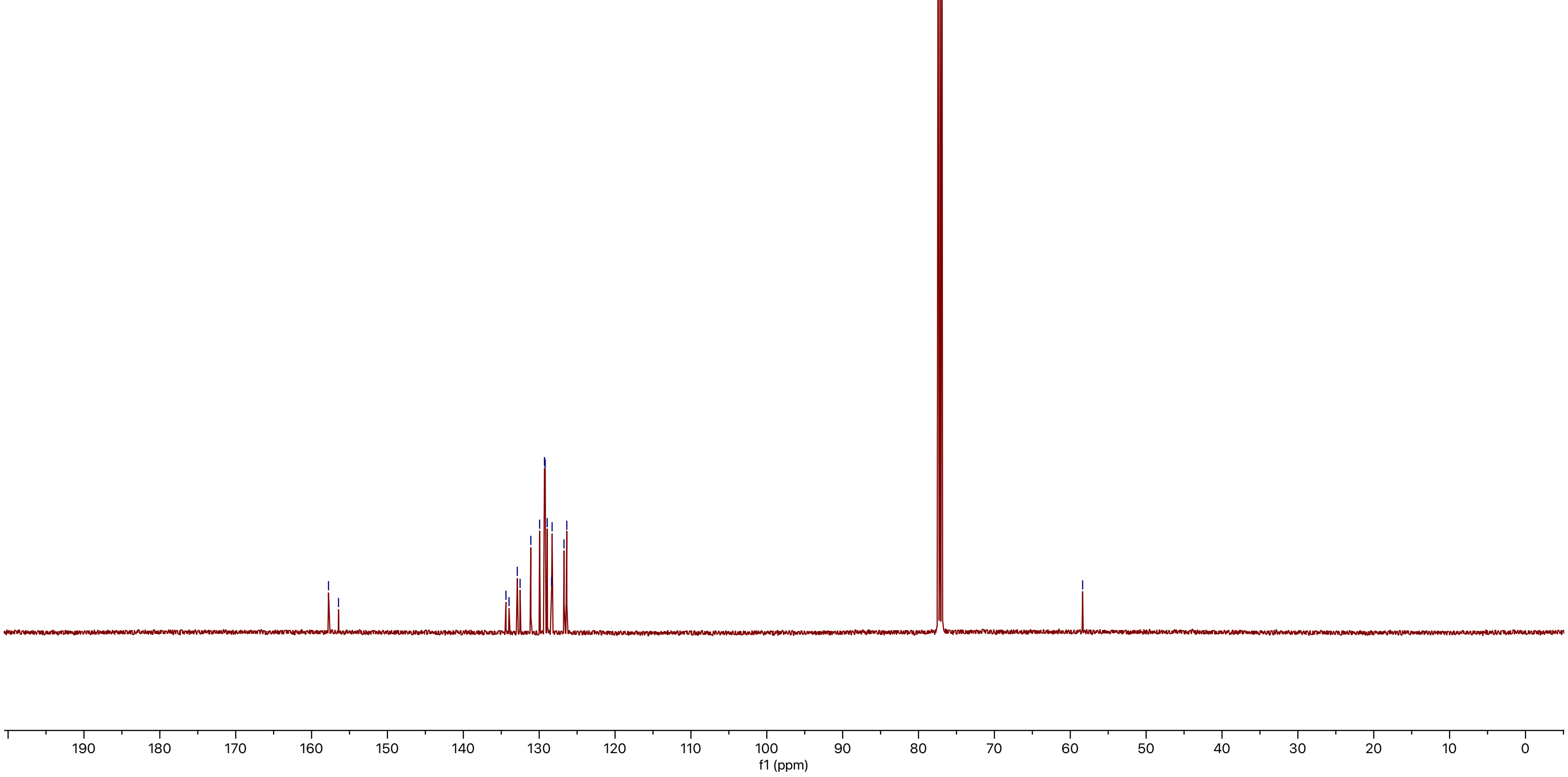

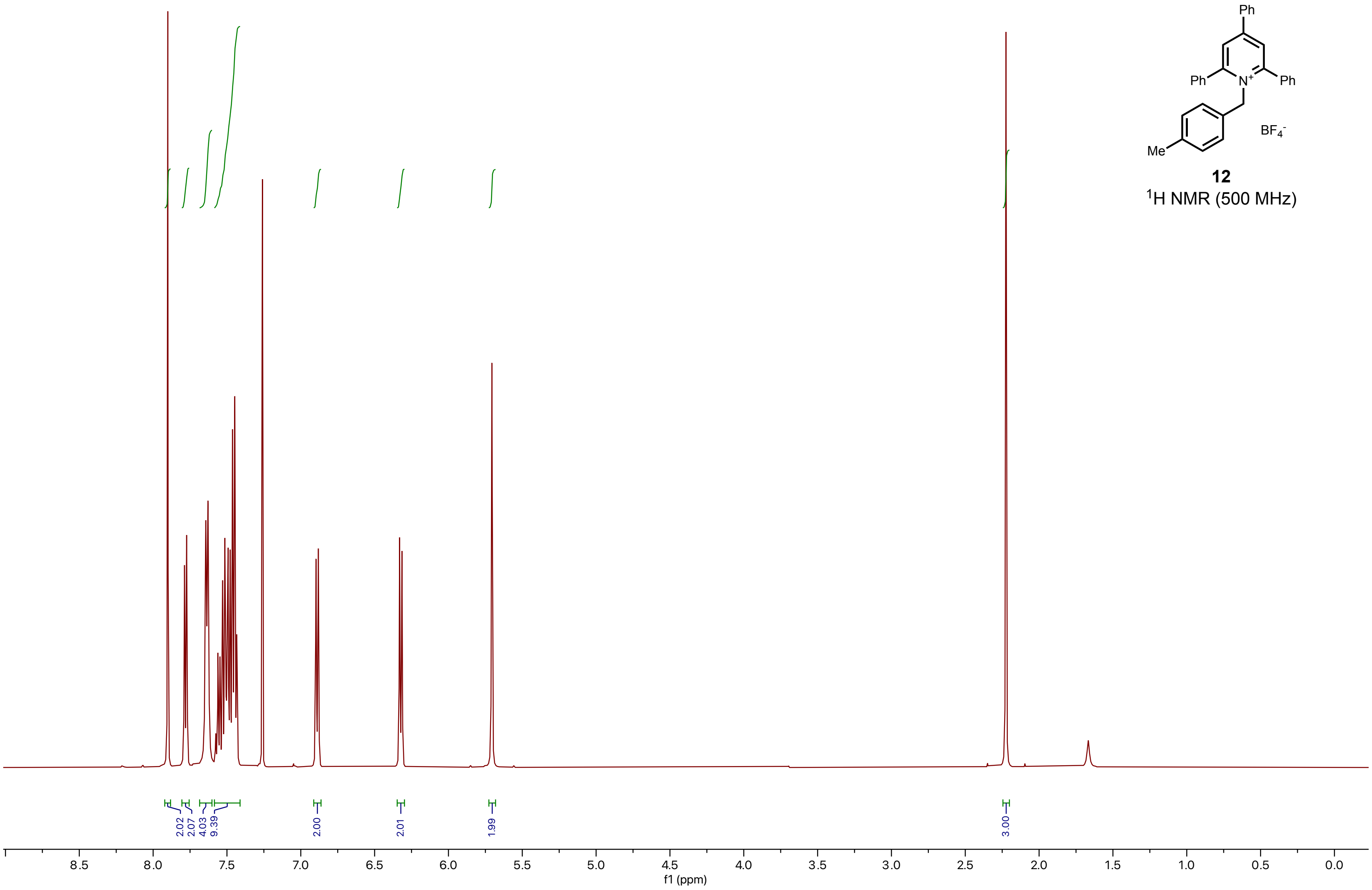

Ph
Ph
N+
Ph
BF4-
Me
12
1H NMR (500 MHz)
2.02
2.07
4.03
9.39
2.00
2.01
1.99
3.00
8.5
8.0
7.5
7.0
6.5
6.0
5.5
5.0
4.5
4.0
3.5
3.0
2.5
2.0
1.5
1.0
0.5
0.0
f1 (ppm)

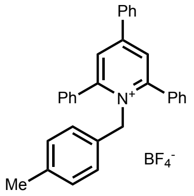

12

^{13}C NMR (126 MHz)

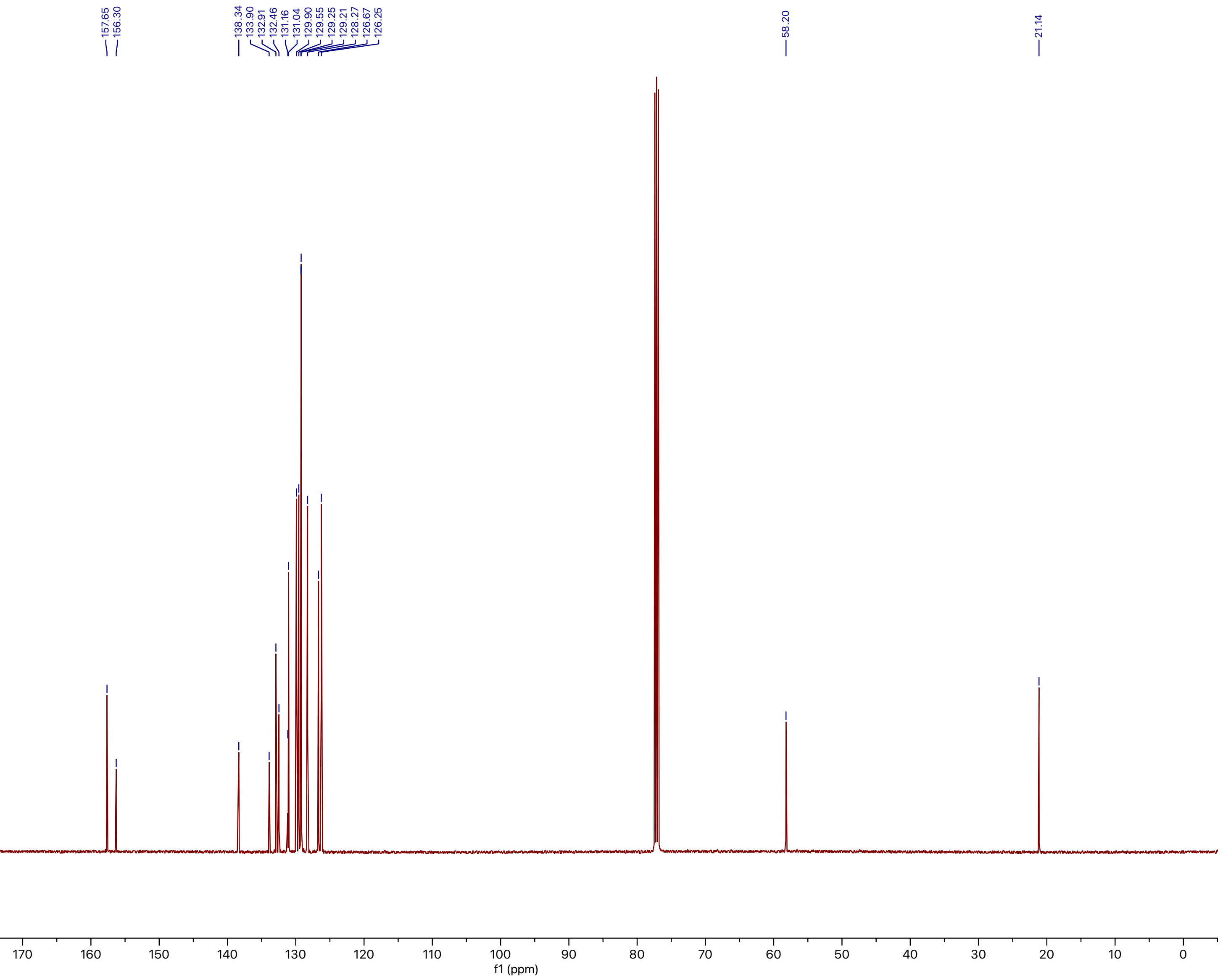

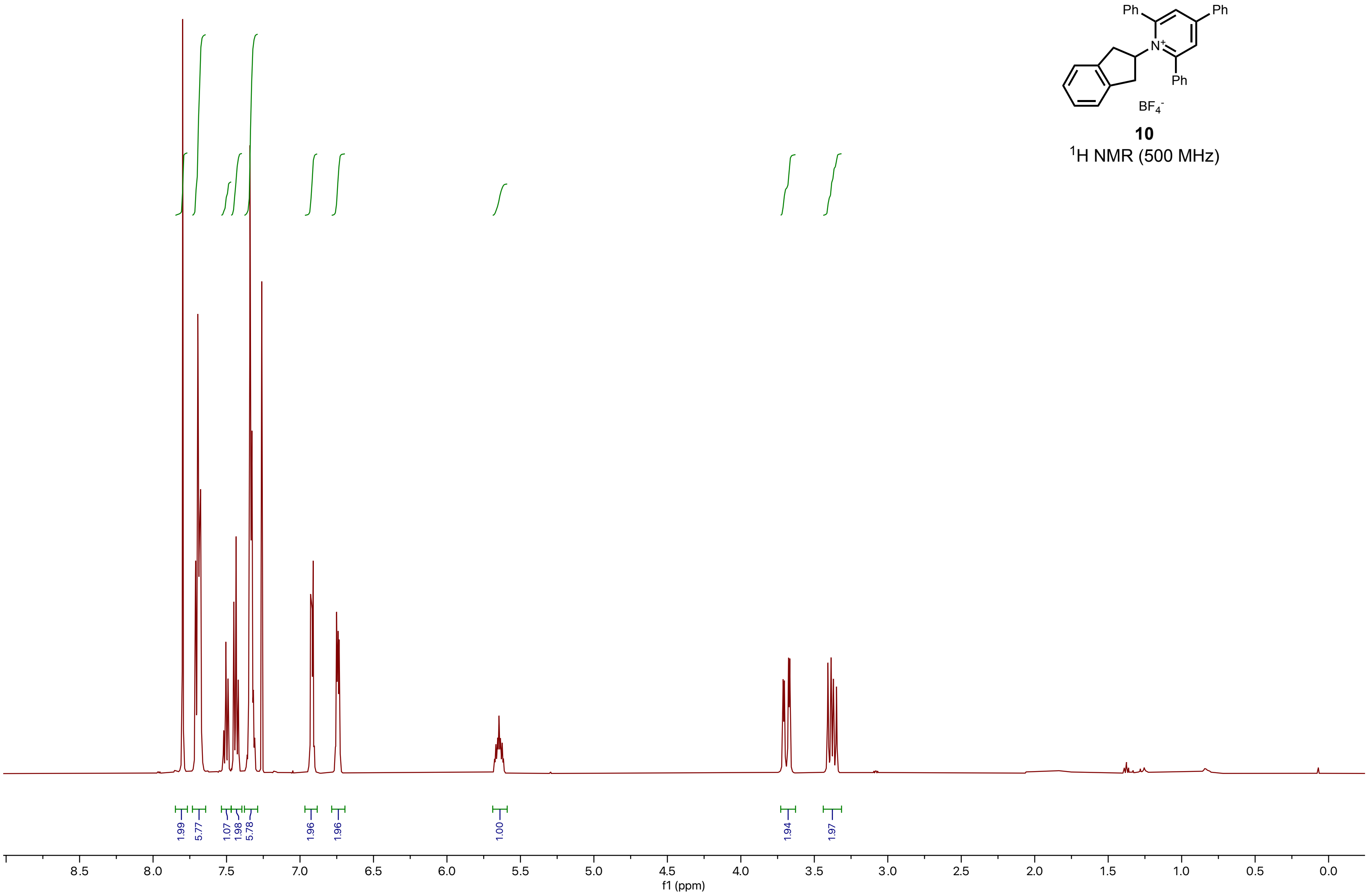

Ph
Ph
N+
Ph
BF4-
10
1H NMR (500 MHz)
1.99
5.77
1.07
1.98
5.78
1.96
1.96
1.00
1.94
1.97
8.5
8.0
7.5
7.0
6.5
6.0
5.5
5.0
4.5
4.0
3.5
3.0
2.5
2.0
1.5
1.0
0.5
0.0
f1 (ppm)

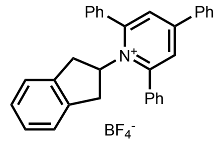

10

^{13}C NMR (126 MHz)

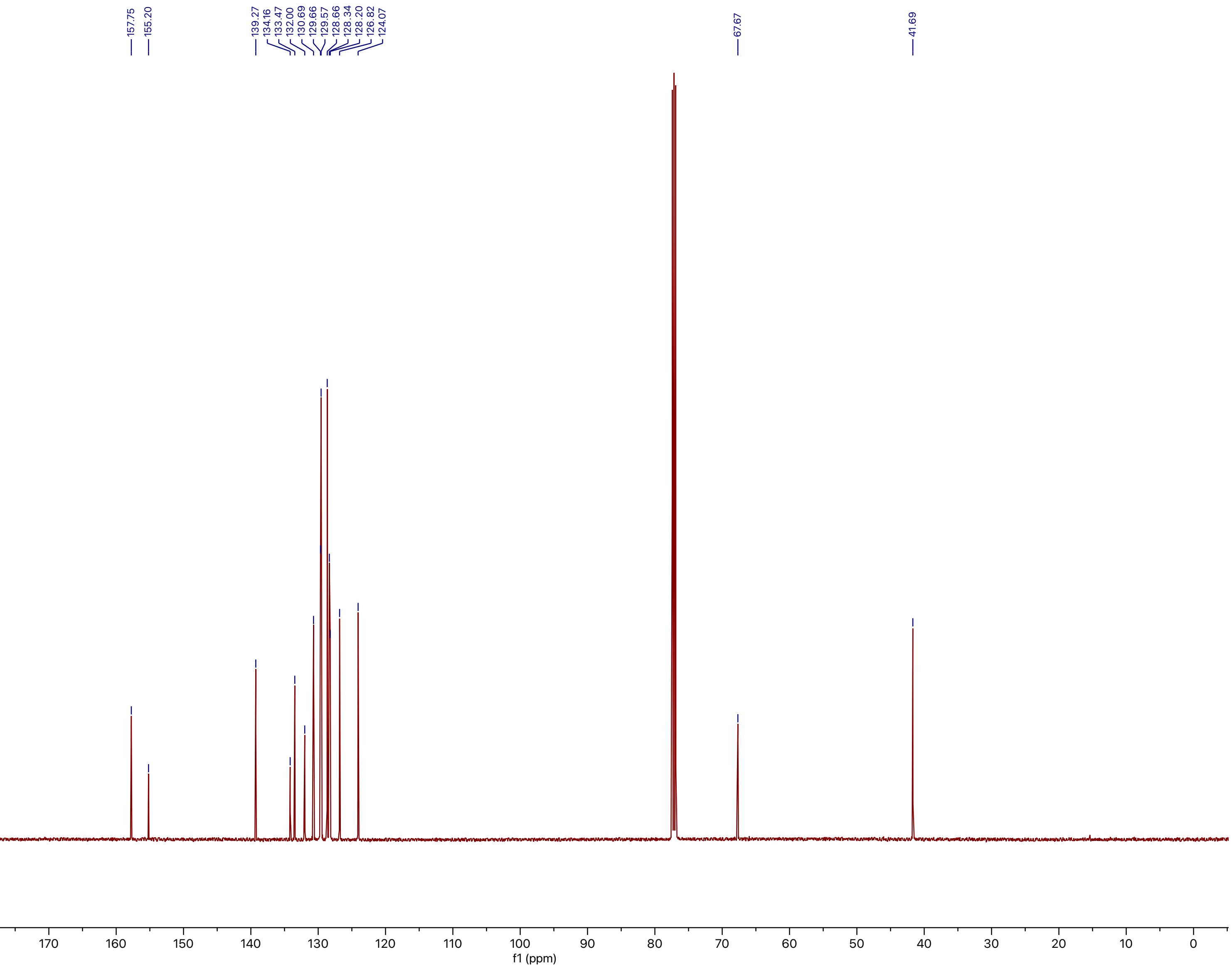

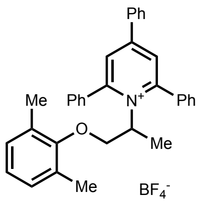

13

^{1}H NMR (500 MHz)

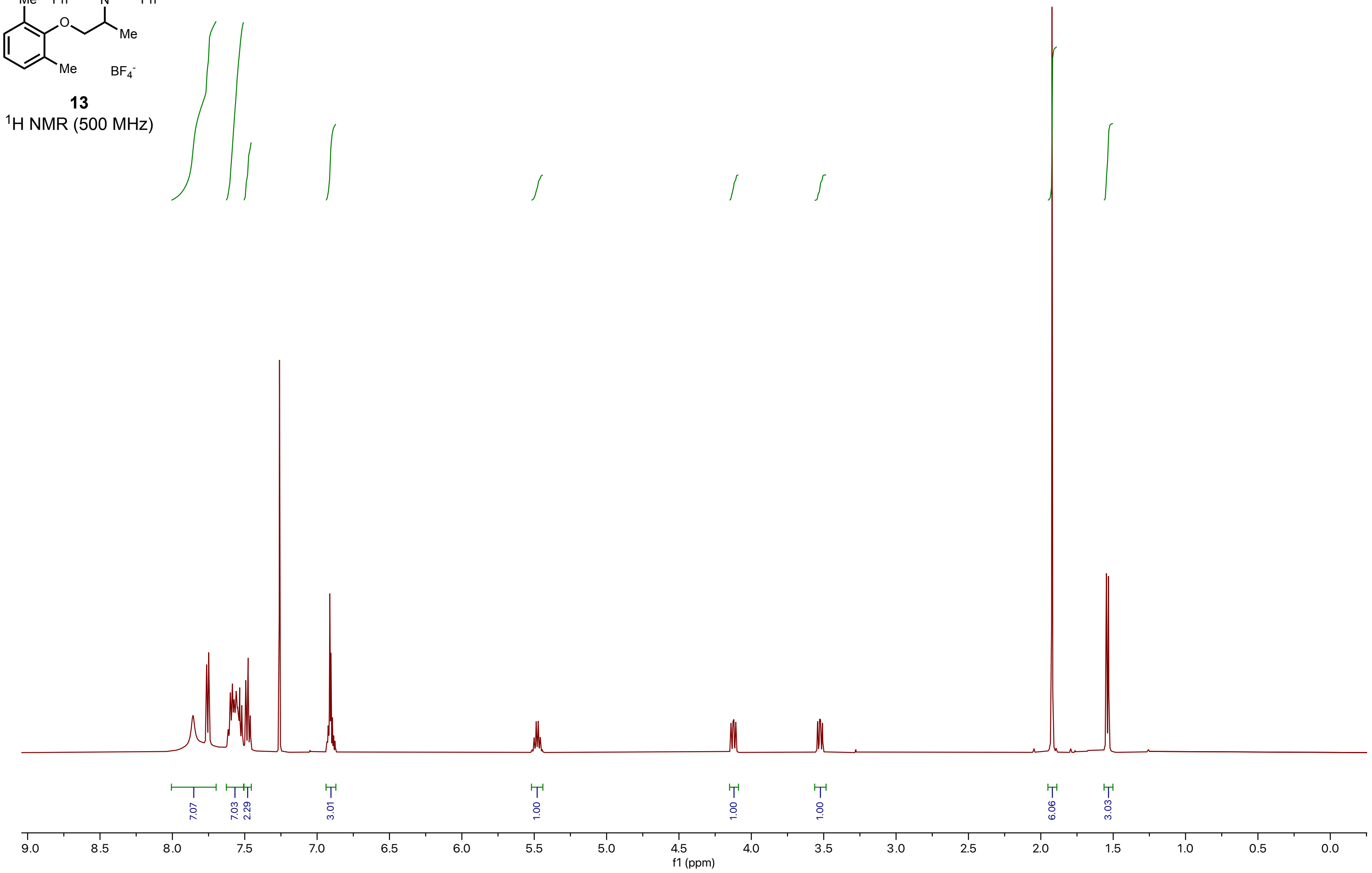

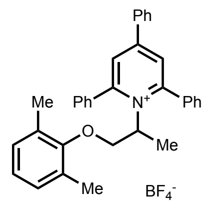

13

^{13}C NMR (126 MHz)

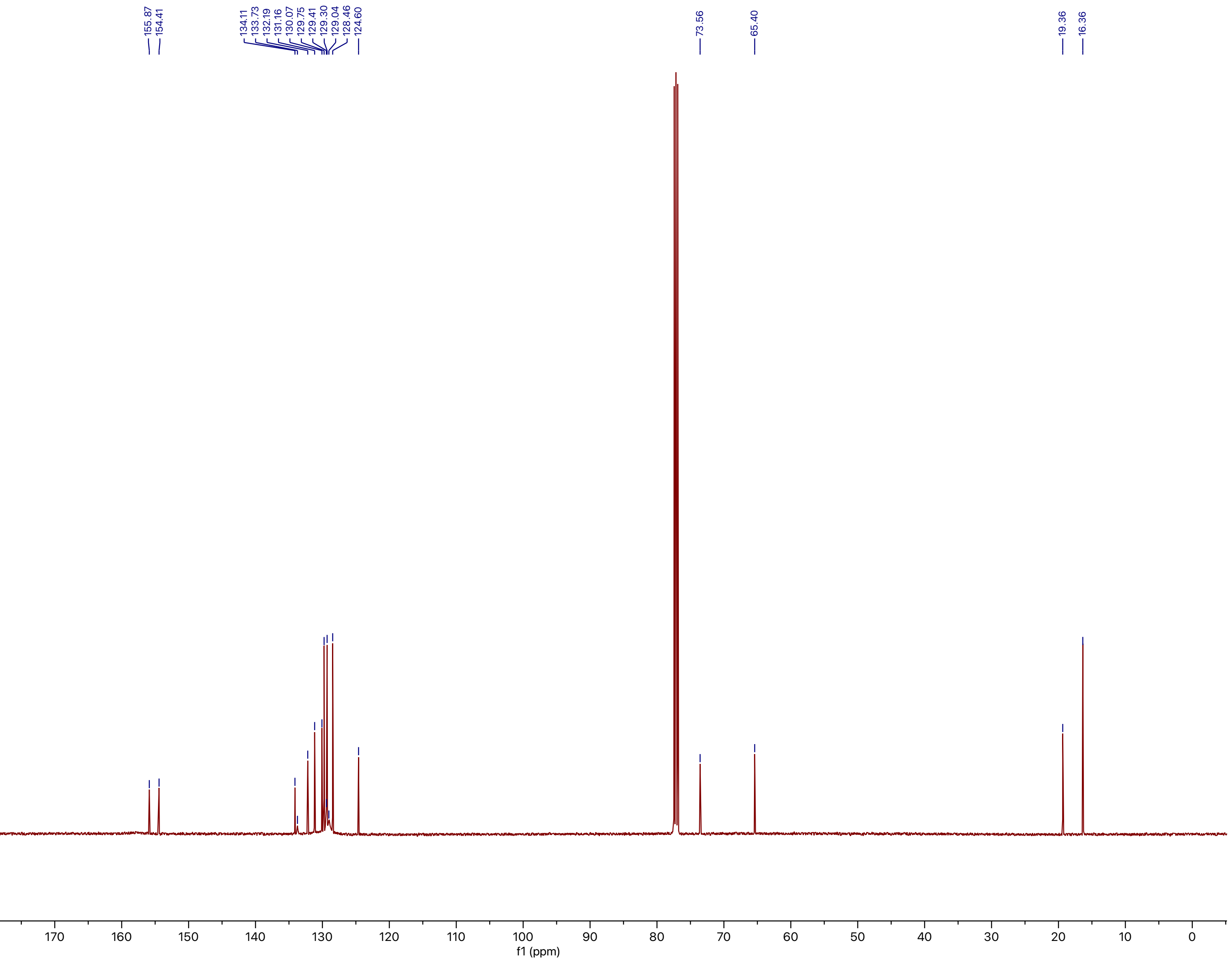

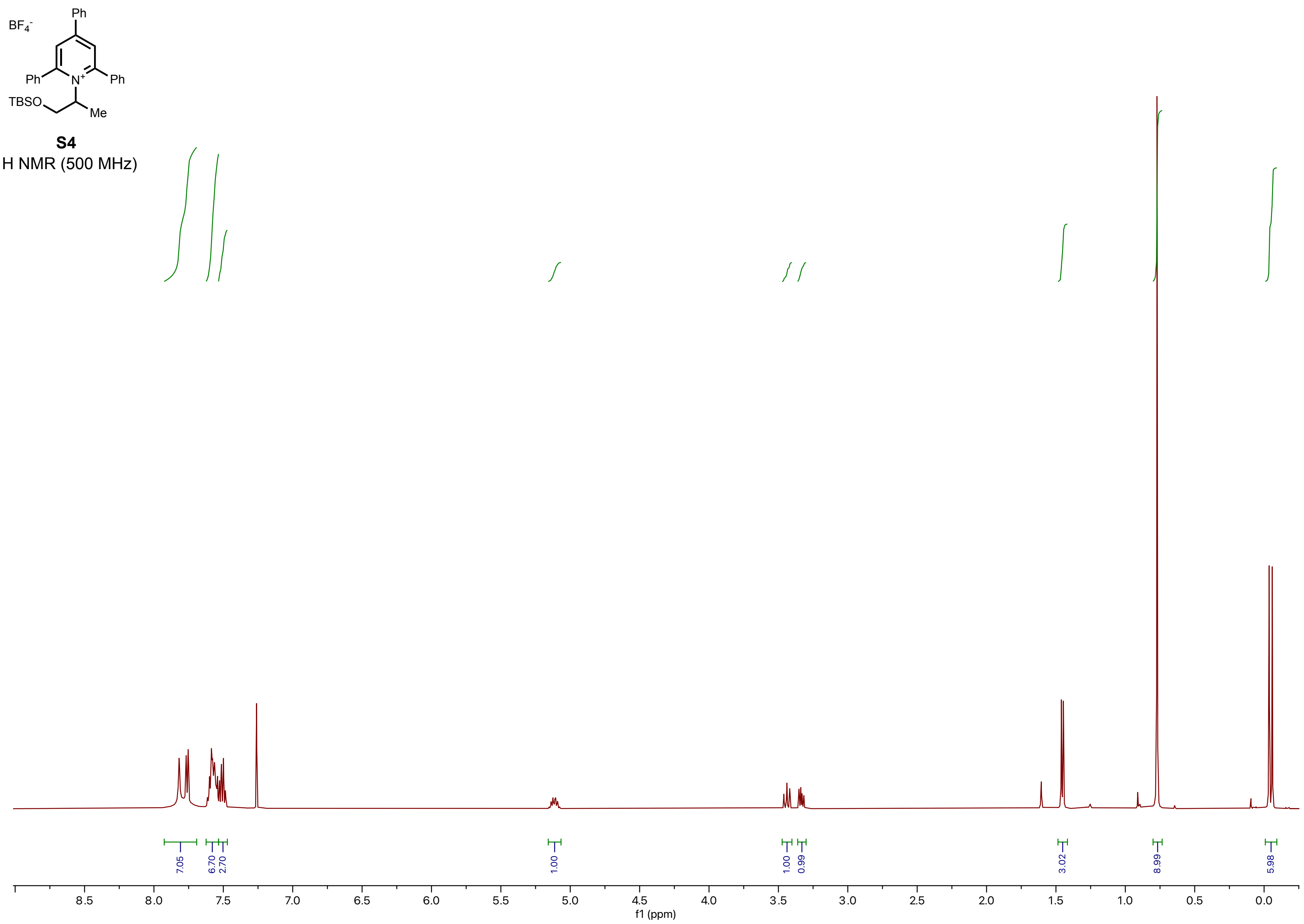

S4

^{1}H NMR (500 MHz)

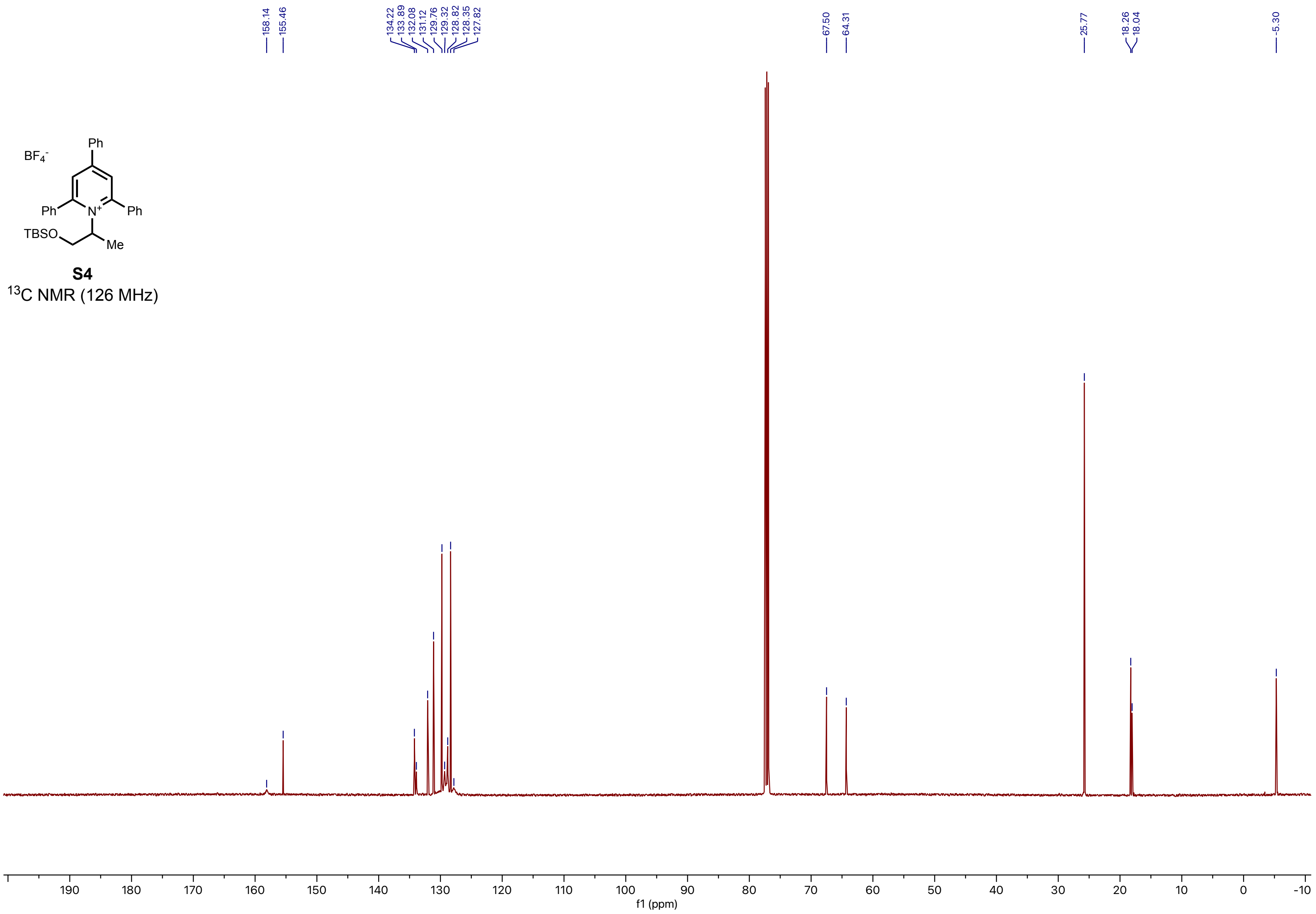
158.14
155.46
134.22
133.89
132.08
131.12
129.76
129.32
128.82
128.35
127.82
67.50
64.31
25.77
18.26
18.04
-5.30
BF4-
Ph
Ph
N+
Ph
TBSO
Me
S4
13C NMR (126 MHz)
190
180
170
160
150
140
130
120
110
100
90
80
70
60
50
40
30
20
10
0
-10
f1 (ppm)

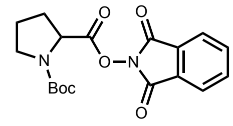

1
^{1}H NMR (500 MHz)

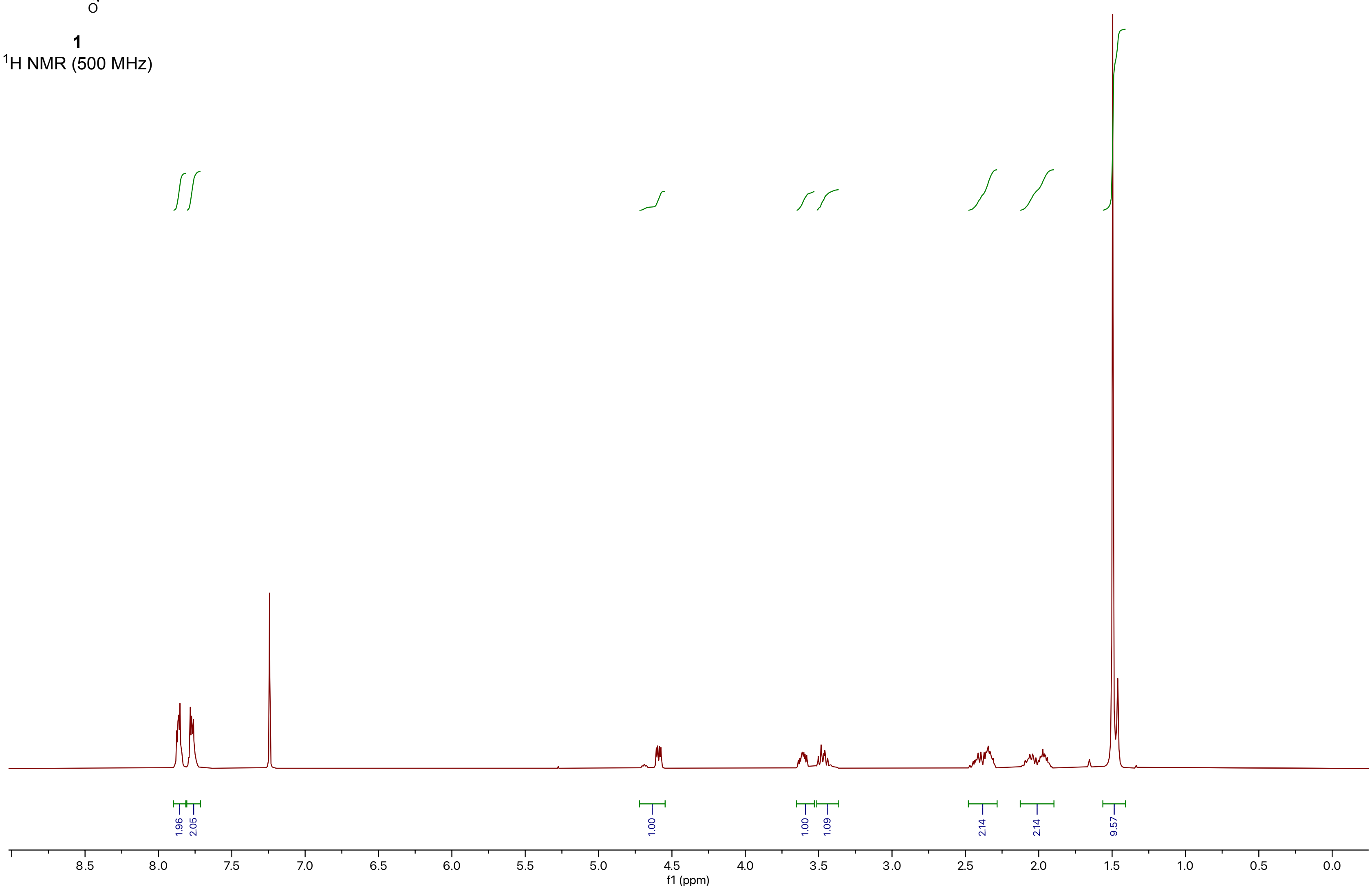

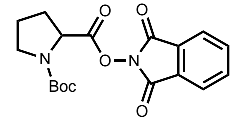

1

^{13}C NMR (126 MHz)

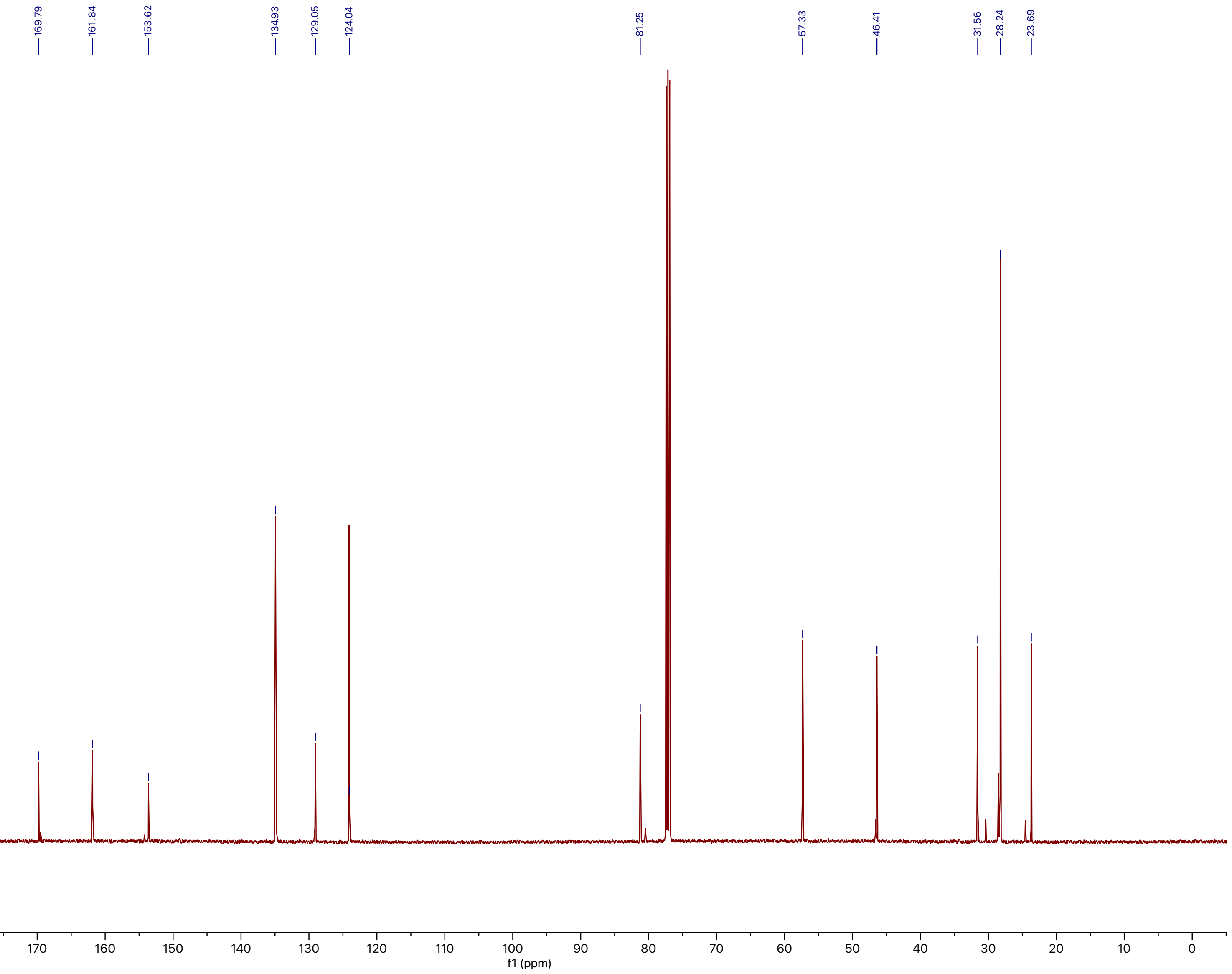

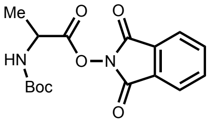

11

^{1}H NMR (500 MHz)

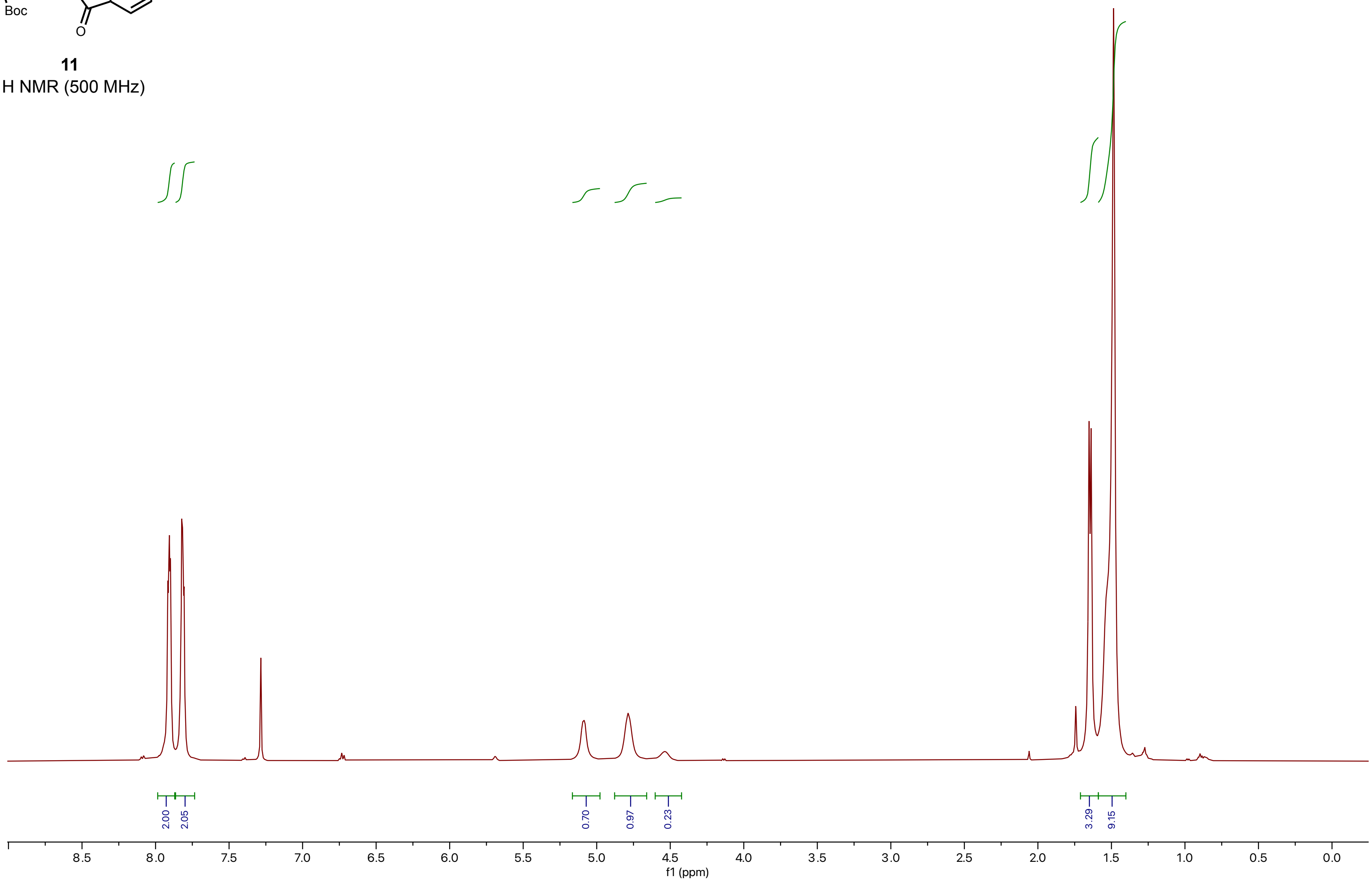

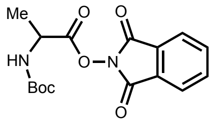

11

^{13}C NMR (126 MHz)

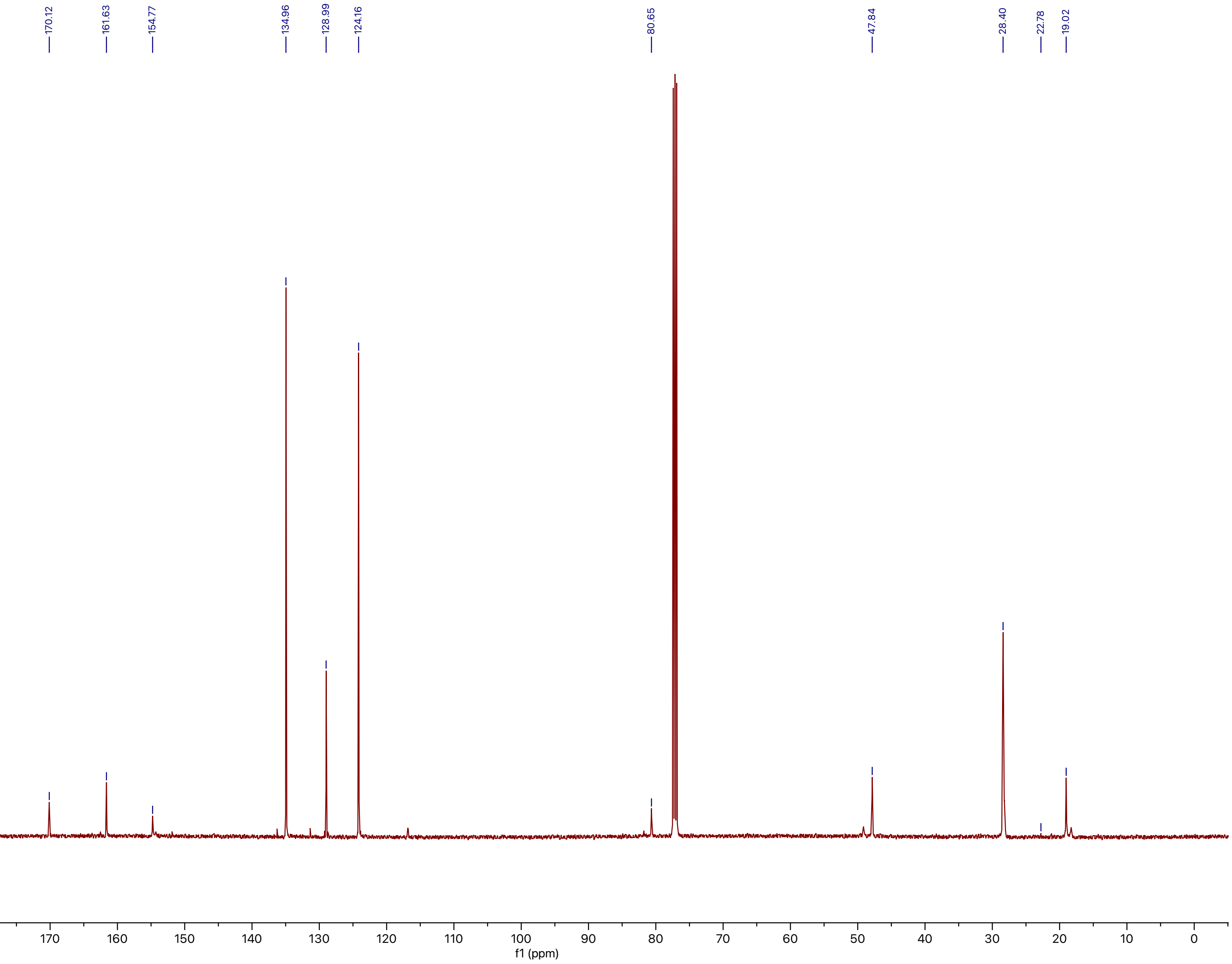

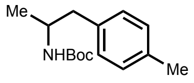

7

^{1}H NMR (500 MHz)

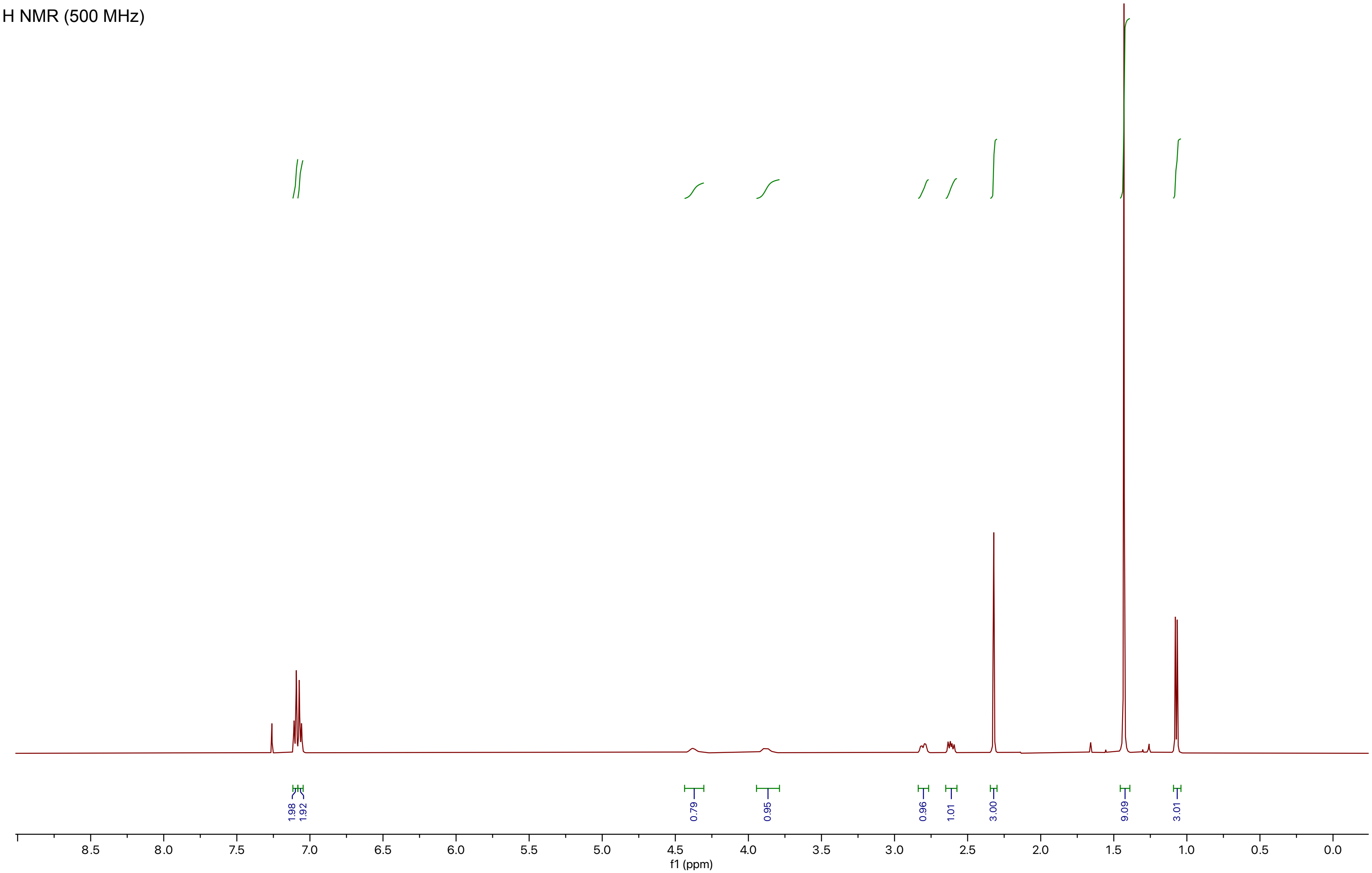

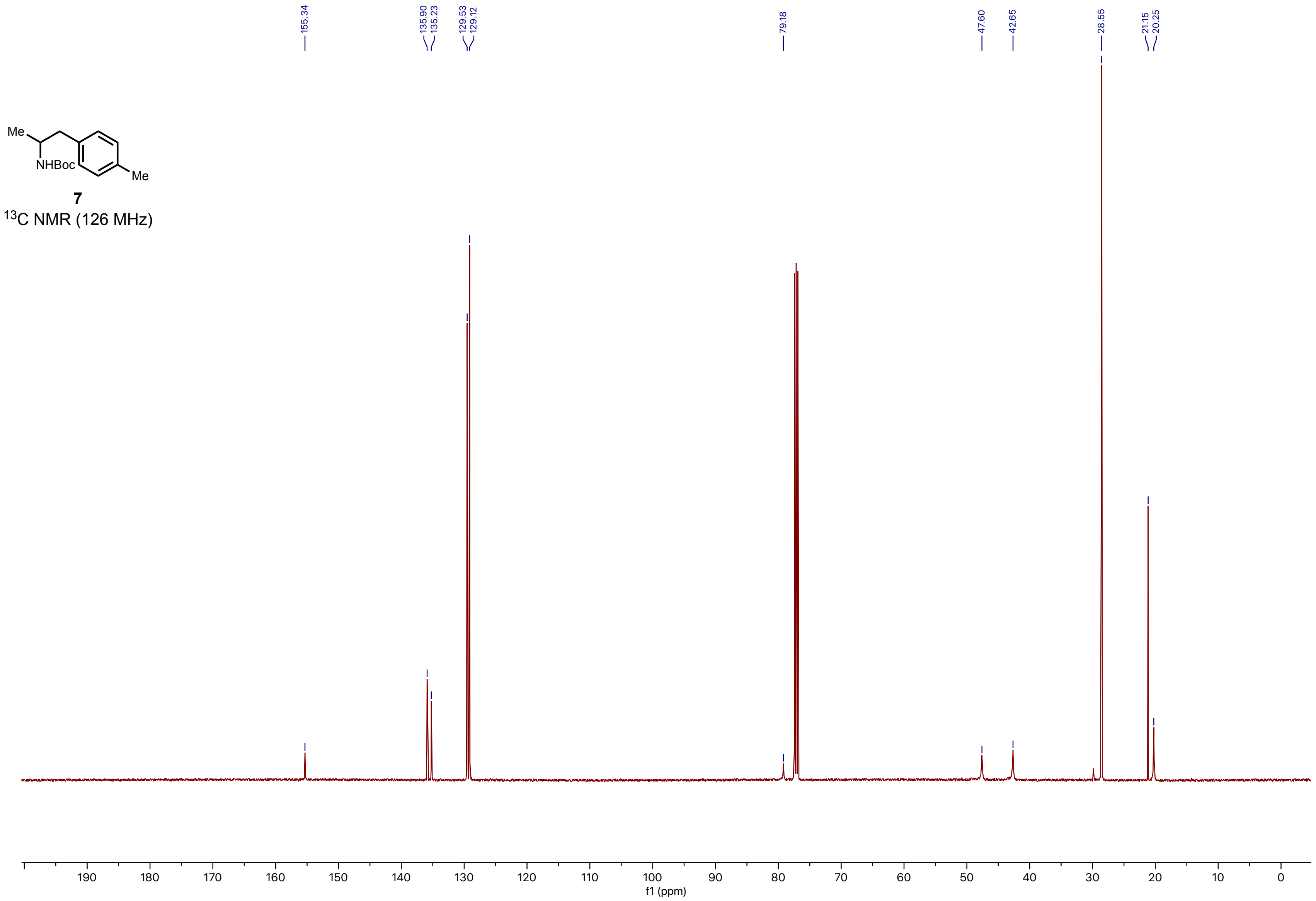

Me
NHBoc
Me
7
13C NMR (126 MHz)
155.34
135.90
135.23
129.53
129.12
79.18
47.60
42.65
28.55
21.15
20.25
190
180
170
160
150
140
130
120
110
100
90
80
70
60
50
40
30
20
10
0
f1 (ppm)

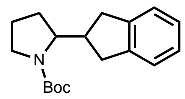

9

^{1}H NMR (500 MHz)

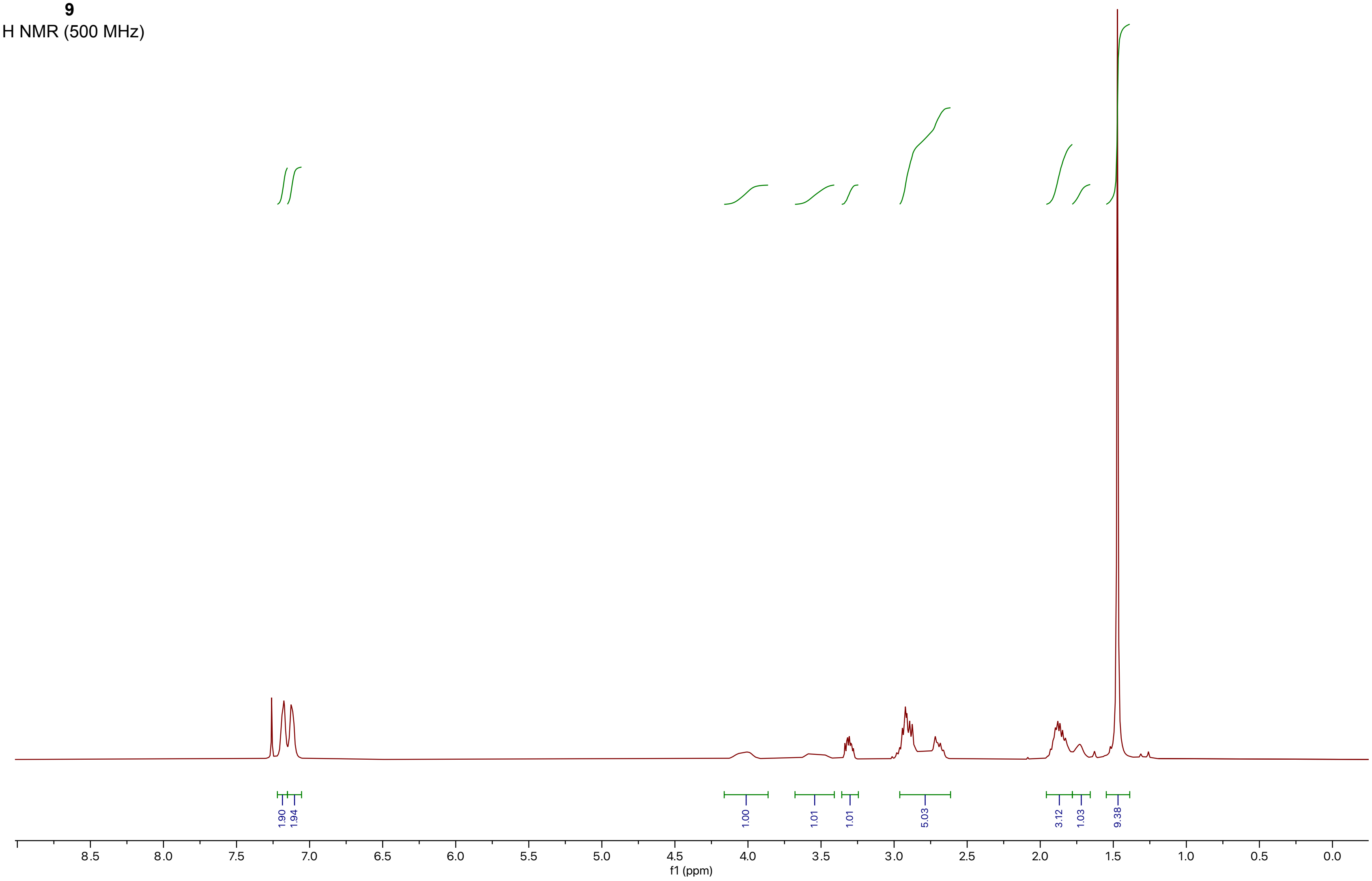

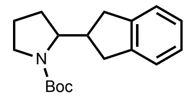

9

^{13}C NMR (126 MHz)

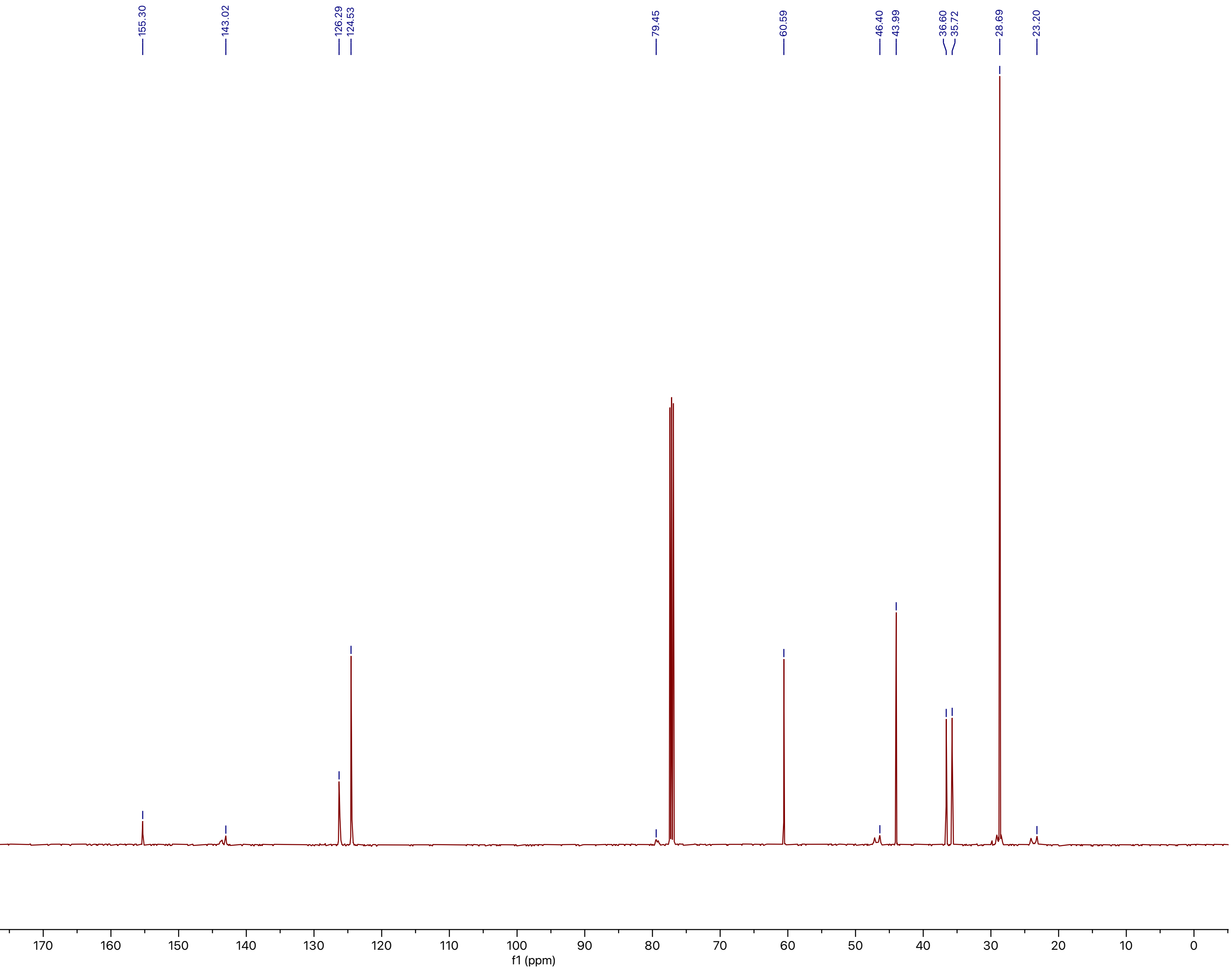

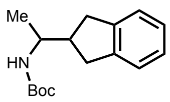

8

^{1}H NMR (500 MHz)

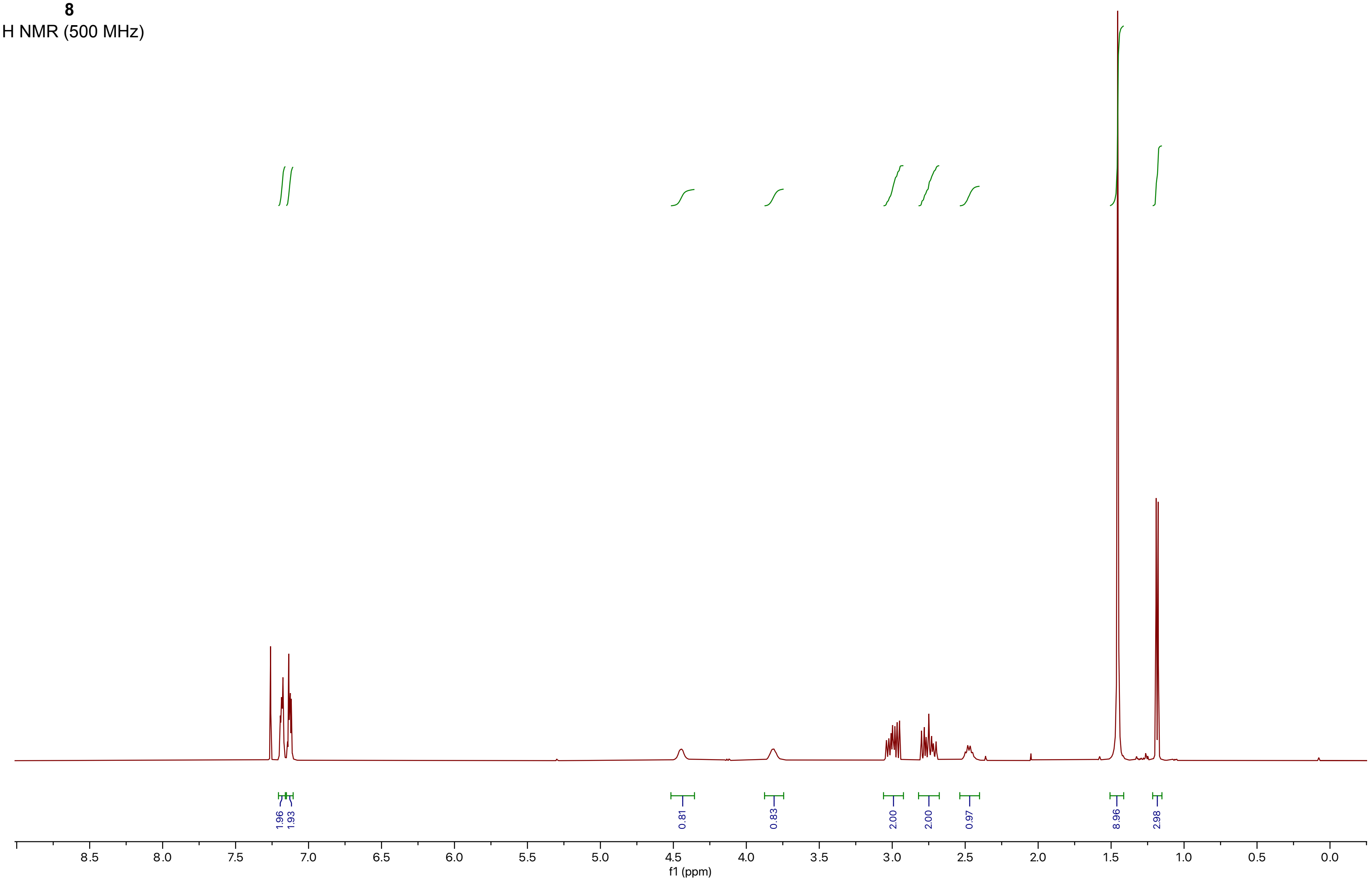

8
^{13}C NMR (126 MHz)

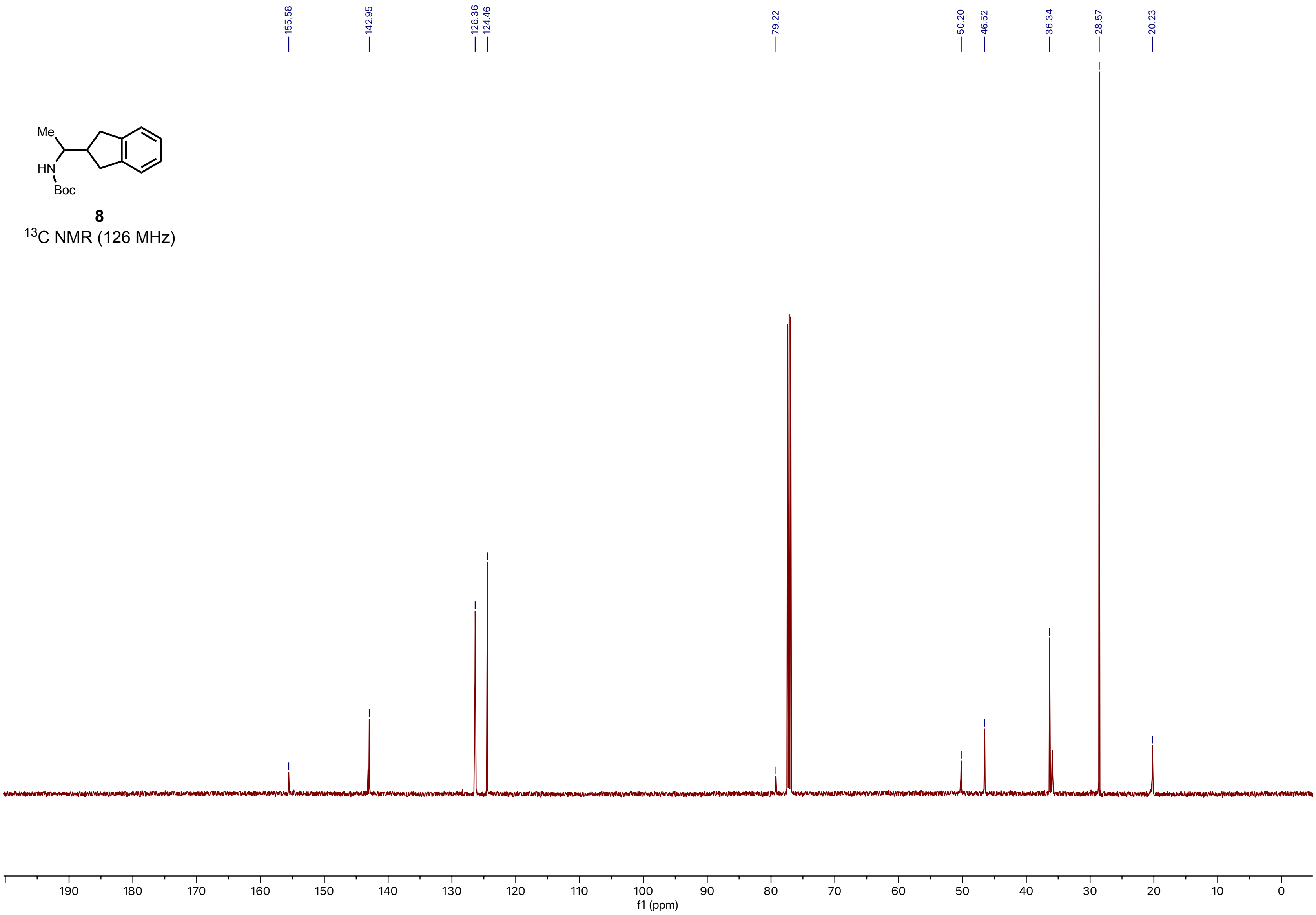

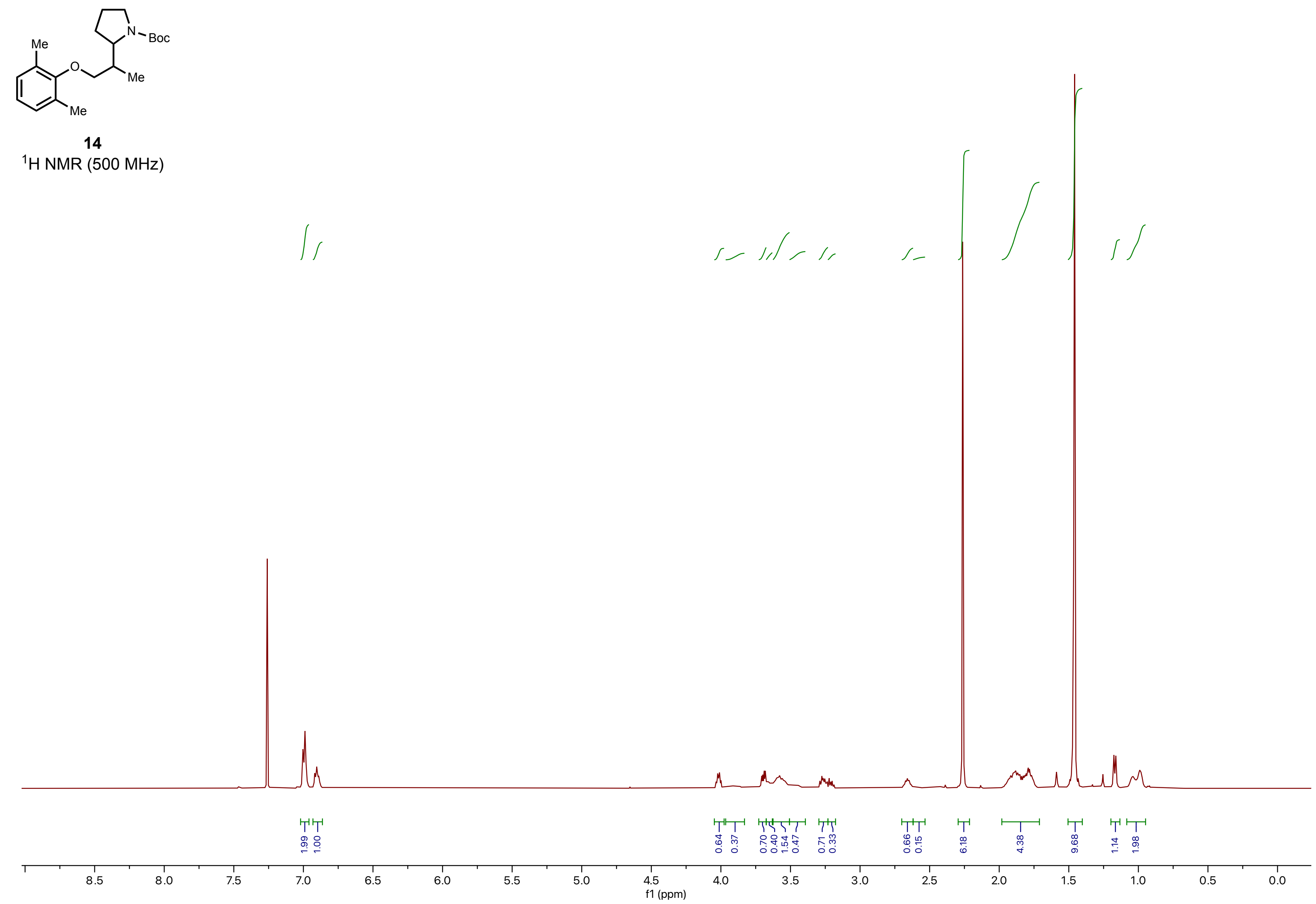

Me
N
Boc
O
Me
Me
14
1H NMR (500 MHz)
1.99
1.00
0.64
0.37
0.70
0.40
1.54
0.47
0.71
0.33
0.66
0.15
6.18
4.38
9.68
1.14
1.98
8.5
8.0
7.5
7.0
6.5
6.0
5.5
5.0
4.5
4.0
3.5
3.0
2.5
2.0
1.5
1.0
0.5
0.0
f1 (ppm)

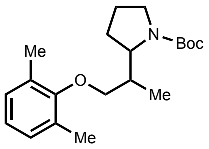

14

^{13}C NMR (126 MHz)

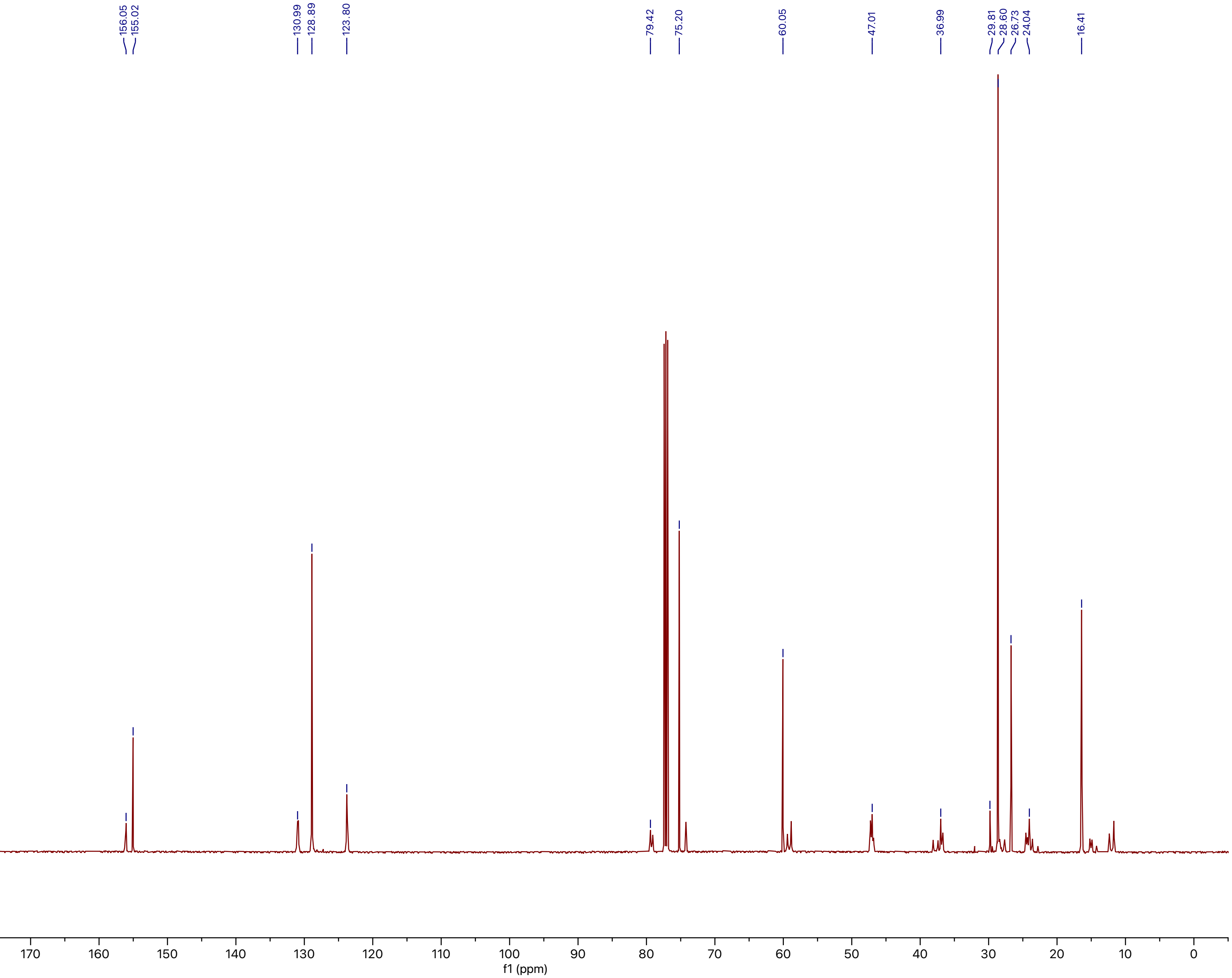

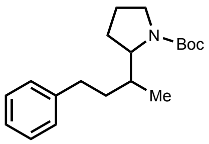

S1

^{1}H NMR (500 MHz)

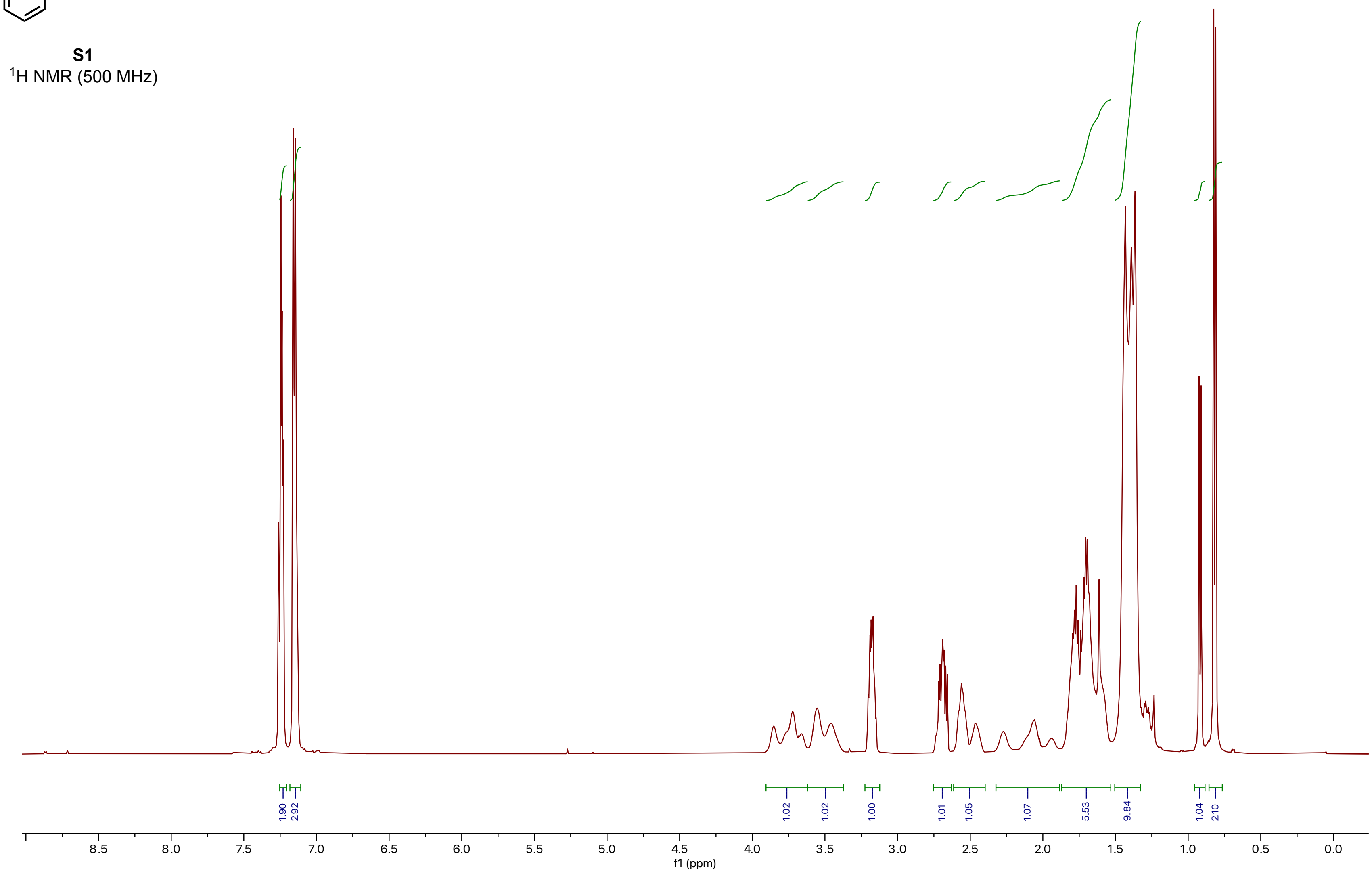

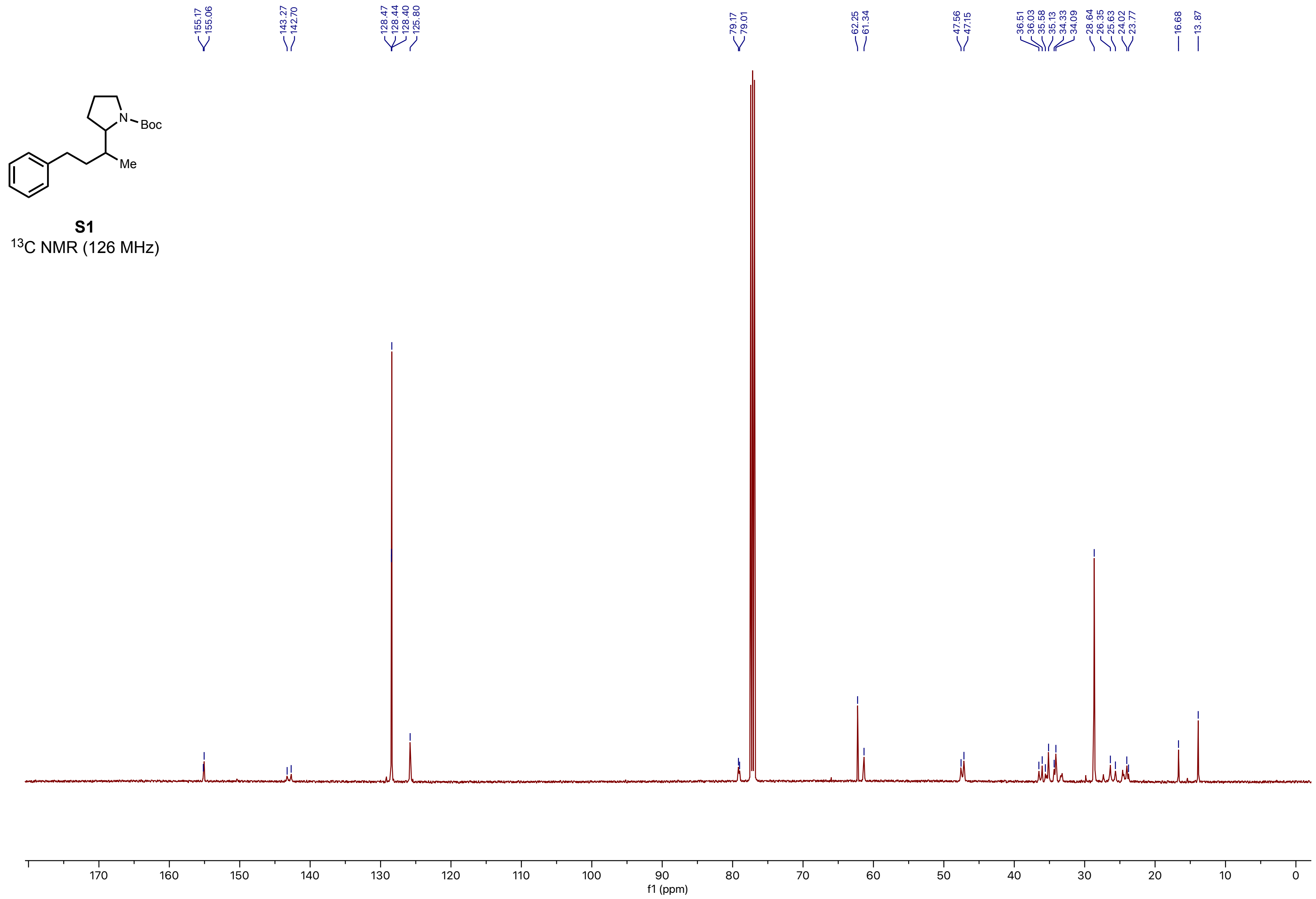
155.17
155.06
143.27
142.70
128.47
128.44
128.40
125.80
79.17
79.01
62.25
61.34
47.56
47.15
36.51
36.03
35.58
35.13
34.33
34.09
28.64
26.35
25.63
24.02
23.77
16.68
13.87
N
Boc
Me
S1
13C NMR (126 MHz)
170
160
150
140
130
120
110
100
90
80
70
60
50
40
30
20
10
0
f1 (ppm)

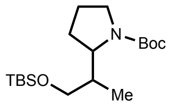

S2

^{1}H NMR (500 MHz)

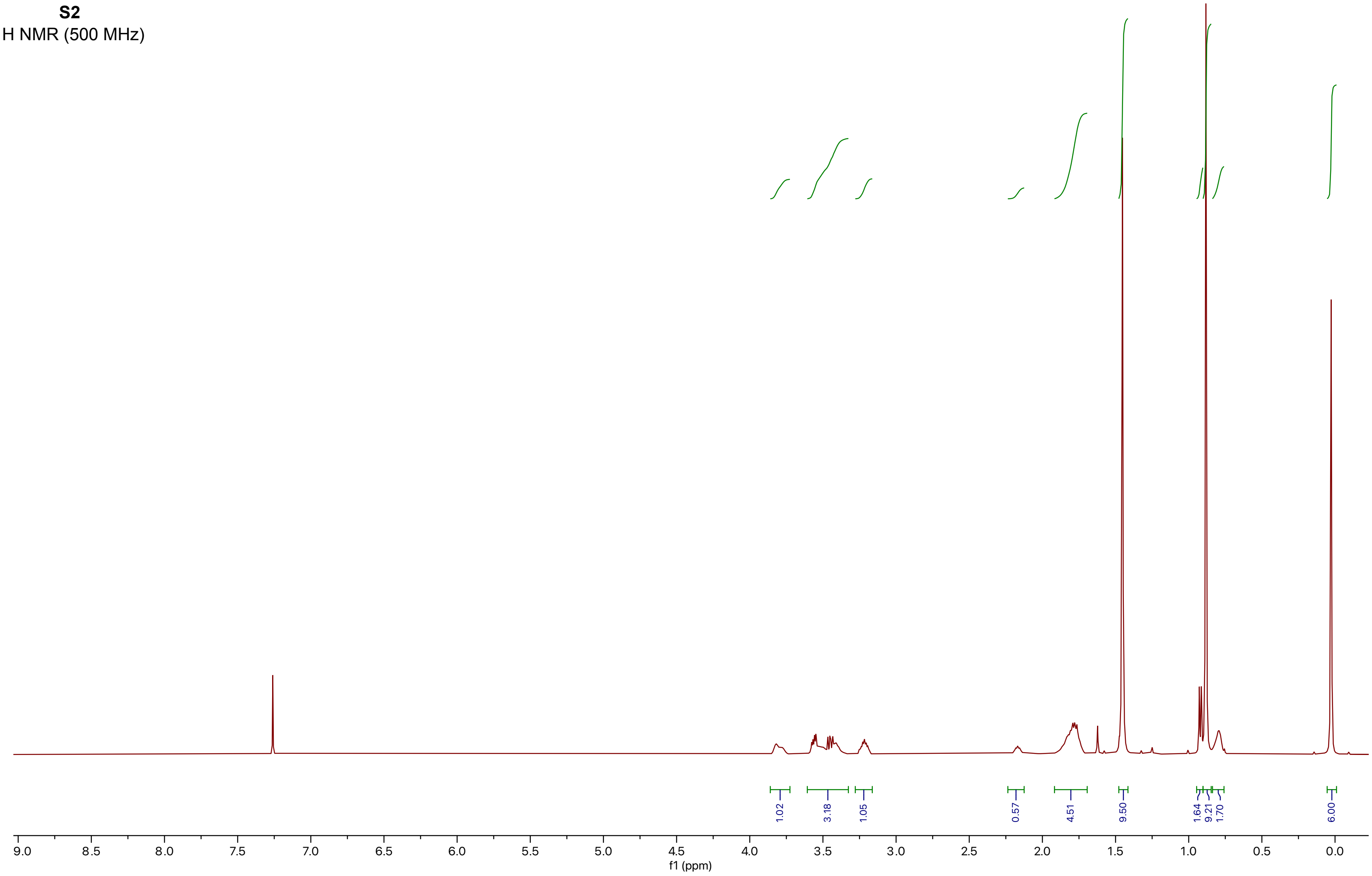

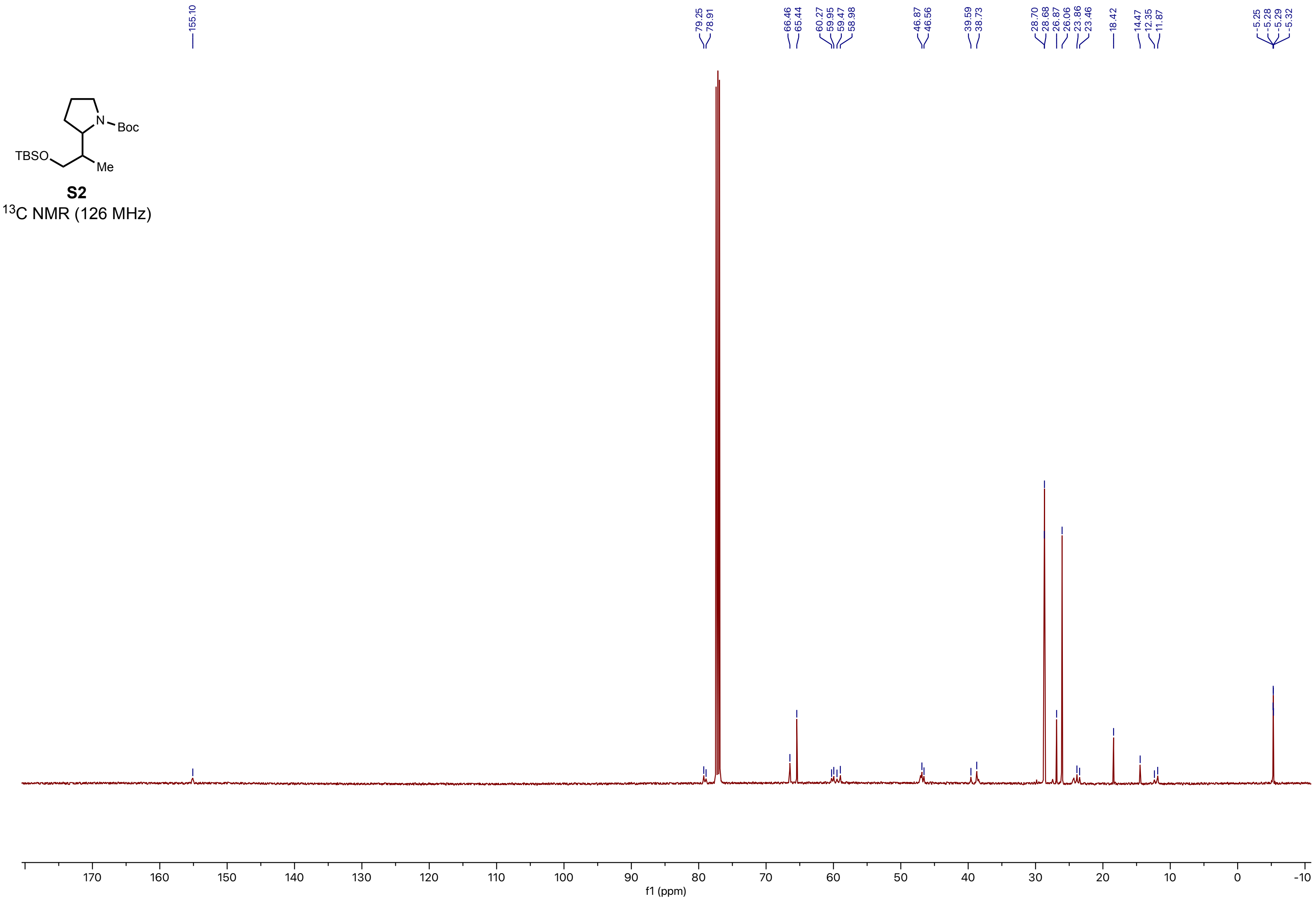

S2

^{13}C NMR (126 MHz)
